# Benzoxazolone-5-Urea Derivatives as Human Soluble Epoxide Hydrolase (sEH) Inhibitors

**DOI:** 10.1021/acsomega.2c06936

**Published:** 2023-01-04

**Authors:** Tugce Gur Maz, Beyzanur Koc, Paul M. Jordan, Kübra İbiş, Burcu Çalışkan, Oliver Werz, Erden Banoglu

**Affiliations:** † Department of Pharmaceutical Chemistry, Faculty of Pharmacy, Gazi University, Taç Sok. No:3 Yenimahalle, 06560 Ankara, Turkey; ‡ Department of Pharmaceutical/Medicinal Chemistry, Institute of Pharmacy, Friedrich Schiller University Jena, Philosophenweg 14, D-7743 Jena, Germany

## Abstract

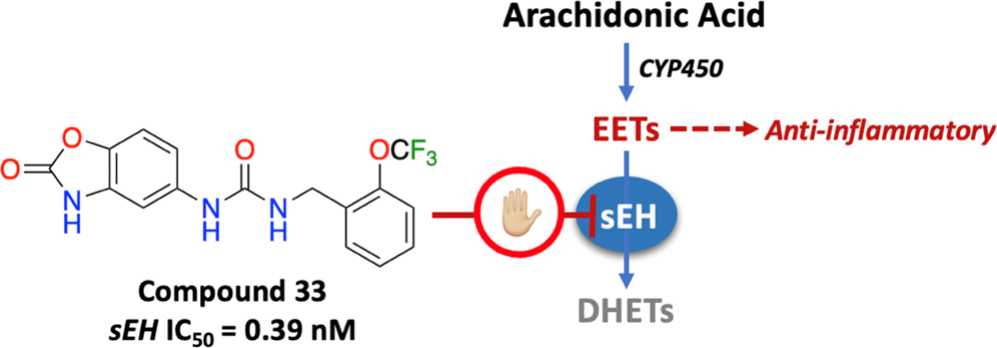

Inhibition of soluble epoxide hydrolase (sEH) is indicated as a new therapeutic modality against a variety of inflammatory diseases, including metabolic, renal, and cardiovascular disorders. In our ongoing research on sEH inhibitors, we synthesized novel benzoxazolone-5-urea analogues with highly potent sEH inhibitory properties inspired by the crystallographic fragment scaffolds incorporating a single H-bond donor/acceptor pair. The tractable SAR results indicated that the aryl or benzyl fragments flanking the benzoxazolone-urea scaffold conferred potent sEH inhibition, and compounds **31–39** inhibited the sEH activity with IC_50_ values in the range of 0.39–570 nM. Docking studies and molecular dynamics simulations with the most potent analogue **33** provided valuable insights into potential binding interactions of the inhibitor in the sEH binding region. In conclusion, benzoxazolone-5-ureas furnished with benzyl groups on the urea function can be regarded as novel lead structures, which allow the development of advanced analogues with enhanced properties against sEH.

## 1 Introduction

**Figure 1:**
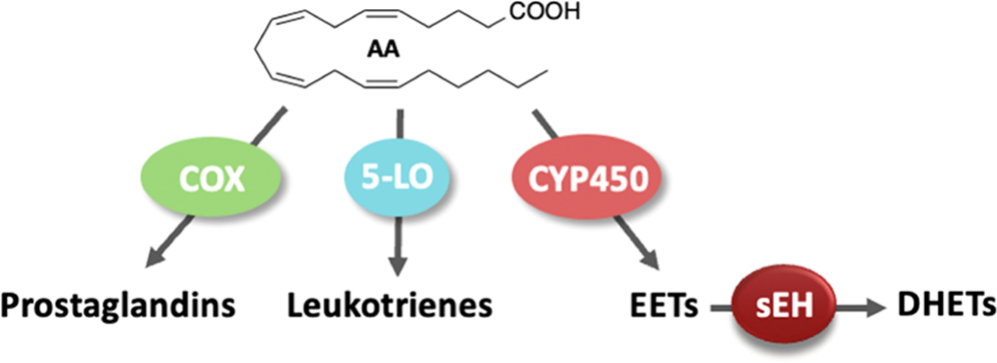
COX-, 5-LO-, and CYP450-mediated AA metabolism.

The arachidonic acid (AA) cascade involves three main enzymatic pathways, which generate endogenous bioactive lipid mediators to regulate numerous physiological and pathophysiological processes such as inflammation, pain, and hypertension (Figure 1).^1^ Cyclooxygenase (COX) and 5-lipoxygenase (5-LO) pathways, which are involved in the biosynthesis of inflammatory prostaglandin (PG)E_2_ and leukotriene (LT)B_4_, have been most studied, leading to the development of classical nonsteroidal anti-inflammatory drugs (NSAIDs) and anti-asthmatics, respectively.^2,3^ However, recently, the cytochrome P(CYP)450 pathway has attracted the attention of researchers as a new molecular point of attack.^4^

CYP450 epoxygenases produce epoxyeicosatrienoic acids (EETs) from AA, which regulate the vascular tone and debilitate the inflammatory signaling in both endothelium and vascular smooth muscles. Thus, EETs demonstrate vasodilatory, anti-inflammatory, anti-apoptotic, and anti-smooth muscle migration activities. Biological actions of EETs are further regulated by the action of soluble epoxide hydrolase (sEH, EC 3.3.2.10) that degrades EETs to their corresponding dihydroxyeicosatrienoic acids (DHETs), whereby the beneficial actions of EETs are diminished or altered.^5,6^ There is ample evidence showing that the increased expression of sEH and reduced levels of EETs have been associated with various pathological responses such as inflammation, hypertension, and neurodegeneration.^5^ Thus, a new therapeutic approach has emerged to inhibit sEH activity to elevate specifically EET levels, thereby prolonging their biological functions to develop efficient therapeutic modalities for related pathologies.^7−9^

**Figure 2:**
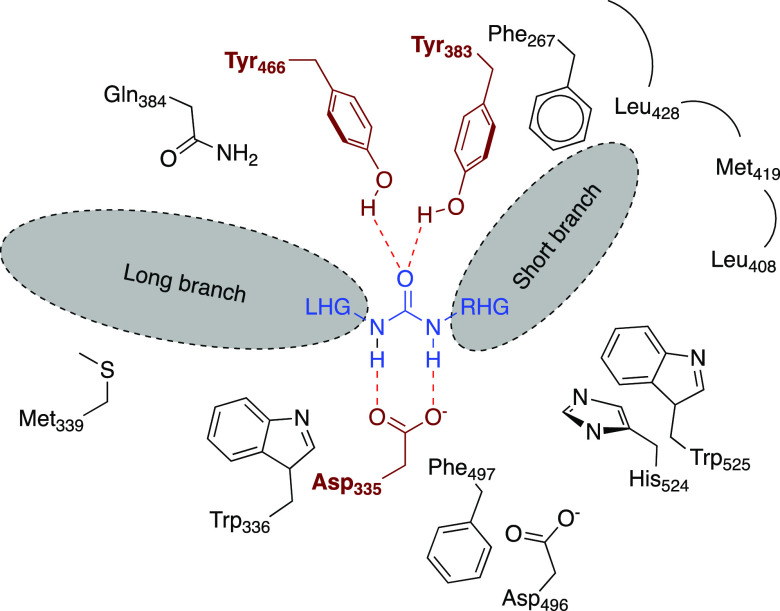
Cartoon representation of the hydrolase domain of human sEH showing the significant amino acids and internal cavities for optimum ligand interactions. LHG, left-hand group; RGH, right-hand group.

sEH is a bifunctional enzyme. In addition to the C-terminal hydrolase, it also possesses an N-terminal phosphatase activity; however, the physiological importance of the N-terminal phosphatase activity remains unclear (Figure 1).^10,11^ The catalytic mechanism of the hydrolase activity of sEH has been well investigated in biochemical studies, and the X-ray crystal structures with sEH inhibitors or chemical fragments revealed the critical structural features for developing sEH inhibitors.^12−15^ For example, urea and amide groups were shown to establish direct interactions in the hydrolase catalytic pocket (Figure 2). More specifically, the amide or urea carbonyl oxygen forms hydrogen bonds with Tyr381 and Tyr465, and the NH of the urea or amide behaves as a hydrogen bond donor to Asp333, thereby acting as primary pharmacophores for ideal binding to the active site. In addition, mainly hydrophobic and bulky fragments (left- and right-hand side groups) flanking the urea or amide pharmacophores can be accommodated in two binding areas, namely, long and short branches, to stabilize the inhibitor ligands at the active site of sEH by space-filling properties.^14^

**Figure 3:**
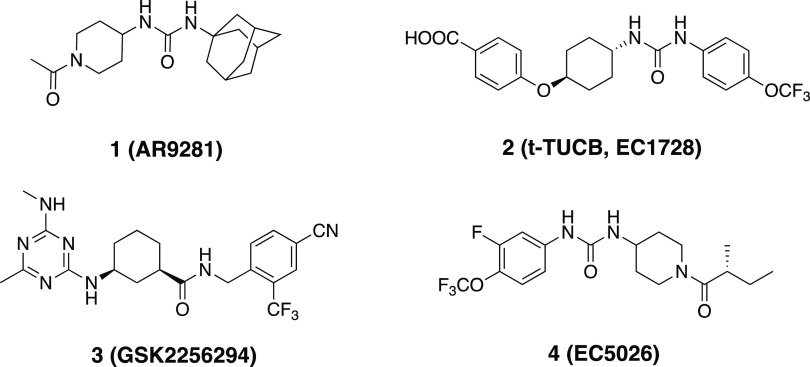
Examples of advanced sEH inhibitors (**1**–**4**).

A detailed understanding of inhibitor binding interactions to the hydrolase domain of sEH has allowed numerous pharmaceutical companies and academic groups to target sEH for developing novel anti-inflammatory and anti-pain agents, of which several benchmark sEH inhibitors (**1**–**4**) are exemplified in Figure 3.^16−19^ Although only a few sEH inhibitors have made it to clinical stages, numerous preclinical studies have unveiled the broad therapeutic potential of sEH inhibitors against various inflammation-related diseases, including neurodegenerative, metabolic, and cardiovascular disorders.^7^

As a continuation of our efforts aiming at interfering with distinct pathways within the AA cascade using various heterocyclic compounds,^20−26^ we here sought to design new lead compounds as sEH inhibitors by taking advantage of the information from sEH-crystallographic fragment screening studies and available SAR data for sEH inhibitor interactions.^12−15^ These inhibitors were constructed by merging an aryl amide or urea pharmacophore, which is common to the medicinal chemistry of known sEH inhibitors, with the benzoxazolone ring that is inspired by the crystallographic fragment screening studies. Thus, we report here our success in identifying new benzoxazolone-urea derivatives that potently inhibit sEH, which is prone to further development as potential anti-inflammatory agents.

## 2 Results and Discussion

### 2.1 Design and Chemistry

Recently, several crystallographic approaches have been employed for fragment screening to identify promising fragment hits to build potential sEH inhibitors.^12,13,15^ Among them, several heterocyclic fragments, such as indolinone (**5**), thiazinone (**6**), quinoxalinone (**7**), pyrimidinedione (**8**), and aminothiazole (**9**) with single H-bond donor/acceptor pair were recognized to establish specific polar interactions, i.e., Gln384, Asp496, and Phe497 in the short and long branches.

**Figure 4:**
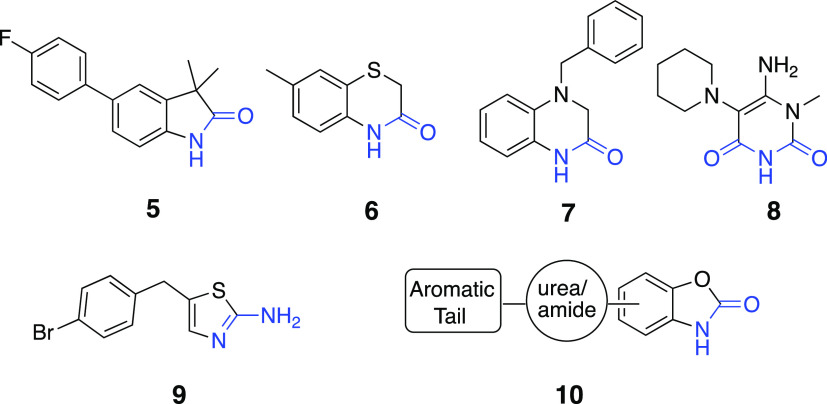
Selected fragment hits (**5**–**9**) from crystallographic screening studies having terminal H-bond donor/acceptor pairs and the general framework of the designed inhibitors (**10**) of the present study.

For our design rationale, we reasoned that the significant polar interactions observed for these fragments could be mimicked by benzoxazolone derivatives to test the hypothesis that new leads can be created. Therefore, the amide/urea group as a primary pharmacophore flanking an aromatic tail is placed along a line with a benzoxazolone core as a secondary pharmacophore to obtain a novel sEH inhibitor scaffold (Figure 4).

**Scheme 1:**
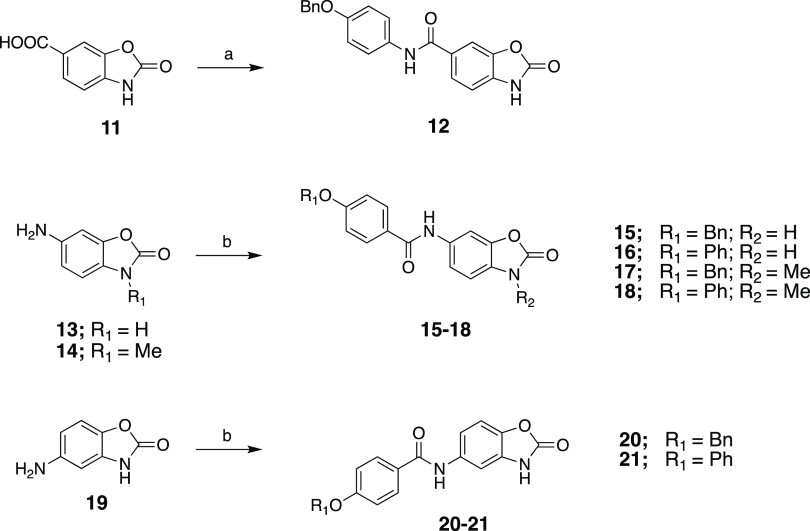
Reaction Conditions and Reagents: (a) EDC·HCl, DMAP, DCM, rt, Overnight; (b) EDC·HCl, HOBt, **13**, and **14** for **15–18**; **19** for **20–21**; DMF, rt, Overnight

**Scheme 2:**
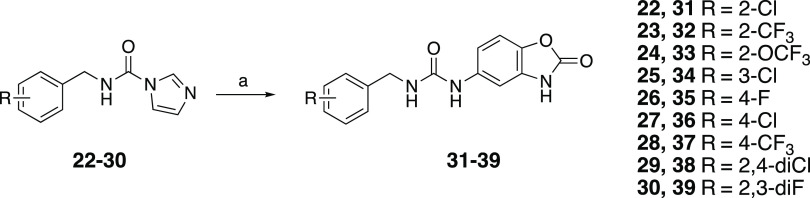
Reaction Conditions and Reagents: (a) Compound **19**, MWI, DIEA, DMF, 1 h

The general synthetic pathways of title compounds are shown in Schemes 1 and 2. For the first amide series (**12**, **15**–**21**), the commercially available starting materials, 6-carboxybenzoxazolone (**11**), 6-aminobenzoxazolone (**13**), 5-aminobenzoxazolone (**19**), or 6-amino-3-methylbenzoxazolone (**14**), which were prepared by us from benzoxazolone (see the Supporting Information), were coupled with appropriate aryl carboxylic acid or amine derivatives under the activation of *N*-ethyl-*N*′-(3-dimethylaminopropyl)carbodiimide hydrochloride (EDC·HCl) in the presence of 4-(dimethylamino)pyridine (DMAP) in CH_2_Cl_2_ or 1-hydroxybenzotriazole (HOBt) in DMF to afford the corresponding amide derivatives (**12**, **15**–**21**) as outlined in Scheme 1. The second series of urea derivatives (**31**–**39**) were synthesized by the reaction of carbamoyl–imidazole intermediates (**22**–**30**), which were prepared from the appropriate benzyl amine HCl salts (see the Supporting Information), with 5-aminobenzoxazolone (**19**) in DMF under microwave heating (Scheme 2). The final purification of the target compounds was done with automated flash chromatography, and purity was confirmed by UPLC (purity was >97%). The compound structures were elucidated by high-resolution mass spectrometry (HRMS) and ^1^H- and ^13^C-NMR spectral data.

### 2.2 Biological Evaluation and SAR

Inspired by the reported crystallographic screening studies,^12,13,15^ a series of aryl amide (**12**, **15**–**21**) or urea (**31**–**39**) derivatives with a benzoxazolone head group were synthesized and assessed for inhibition of human recombinant sEH in a cell-free activity assay^27^ (Tables 1 and 2). We first introduced the aryl amide through its carbonyl to the benzoxazolone ring at 6-position, but the resulting compound **12** was devoid of sEH inhibition (Table 1). However, reversal of the amide group to connect the benzoxazolone ring through NH at the same location was successful, resulting in improved inhibition at 10 μM (**15** and **16** with 70 and 85% inhibition, respectively). Consequently, compounds in which the amide group is attached to the benzoxazolone ring through NH could outperform their direct reversed analogues by more favorable interactions in the central pocket of sEH. In addition, simple methylation of the benzoxazolone NH in **17**–**18** engendered noticeable differences in sEH inhibition relative to the unmethylated counterparts **15**–**16**, implying that a free NH with H-bond donor property at this location is also important for the observed potency. Subsequently, the positioning of the amide group at the 5-position of the benzoxazolone ring also retained the inhibitory activity. This indicates that the amide function can be accommodated at both 5- and 6-positions of the benzoxazolone pendant for sEH inhibition. Also, in pairwise comparisons of the benzyloxy (**15** and **20**) and phenoxy (**16** and **21**) compounds, the phenoxy substituent apparently renders the compounds more potent than the benzyloxy analogues (Table 1).

**Table 1:**
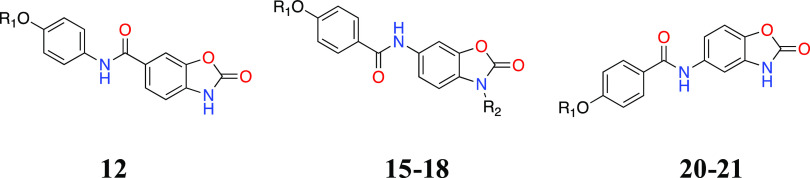
In Vitro sEH Inhibitory Activities of New Benzoxazolone-Amides in a Cell-Free Assay^a^

			sEH activity % of control at		
#	*R*_1_	*R*_2_	1 μM	10 μM	IC_50_ [μM]
**12**	Bn		91.0 ± 6.1	84.4 ± 3.8	>10
**15**	Bn	H	67.8 ± 10.8	28.9 ± 8.1	<10
**16**	Ph	H	49.8 ± 6.3	15.2 ± 5.9	∼1
**17**	Bn	Me	105.8 ± 15.9	115.1 ± 4.9	>10
**18**	Ph	Me	83.7 ± 9.8	91.5 ± 2.8	>10
**20**	Bn		68.2 ± 6.6	30.0 ± 10.5	<10
**21**	Ph		47.4 ± 7.1	14.4 ± 6.8	∼1

a Data (means ± SEM, *n* = 3 determinations) are given as residual activity in percent of control (100%, vehicle, uninhibited control) or IC_50_ values.

**Table 2:**
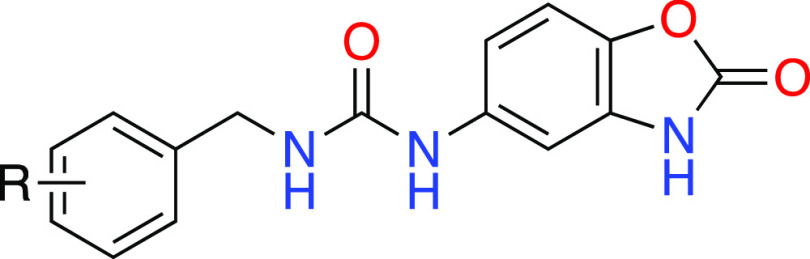
In Vitro sEH Inhibitory Activities of New Benzoxazolone-Urea Derivatives in a Cell-Free Assay^a^

		sEH activity % of control at		
#	*R*	1 μM	10 μM	IC_50_ [nM]
**31**	2-Cl	3.1 ± 6.2	5.8 ± 6.2	9.9 ± 2.9
**32**	2-CF_3_	7.1 ± 5.8	4.7 ± 8.0	2.0 ± 0.42
**33**	2-OCF_3_	1.9 ± 3.2	6.8 ± 5.9	0.39 ± 0.1
**34**	3-Cl	18.6 ± 5.1	7.5 ± 3.4	80 ± 23
**35**	4-F	62.8 ± 3.3	5.2 ± 3.6	570 ± 15
**36**	4-Cl	18.6 ± 2.6	1.9 ± 2.4	120 ± 20
**37**	4-CF_3_	12.3 ± 2.5	1.1 ± 1.8	53 ± 8
**38**	2,4-Cl	0.0 ± 0.0	0.0 ± 0.0	0.7 ± 0.08
**39**	2,3-F	14.2 ± 2.7	0.8 ± 2.0	33 ± 10

a Data (means ± SEM, *n* = 3 determinations) are given as residual activity in percent of control (100%, vehicle, uninhibited control) or IC_50_ values.

The benzyl group appears as a frequently recurring chemical fragment in the architecture of amide or urea-type sEH inhibitors.^4^ Therefore, we extracted this common fragment and combined it with benzoxazolone using a urea backbone with the aim of improving a sEH inhibitory potency in the newly designed analogues (Table 2). Accordingly, we implemented differently substituted benzyl pendants to the urea functionality to produce compounds **31**–**39** with IC_50_ values in the range of 0.39–570 nM (Table 2); however, the inhibition potential was strongly dependent on the substitution pattern of the benzyl group. Briefly, compound **33** with 2-OCF_3_ on the benzyl group was most potent with an IC_50_ of 0.39 nM, while smaller substituents such as Cl (**31**) and CF_3_ (**32**) at this position caused a decrease in the inhibitory potency. This implies that voluminous substituents at this position are better accommodated at the binding site. 3- or 4-substituted benzyl analogues (**34**–**37**) were not pursued further as they showed a marked loss of inhibitory activity (135- to 1460-fold). When the disubstitution pattern was briefly explored, the 2,4-Cl analogue (**38**) was also found to be a superior sEH inhibitor over 2,3-F congener with IC_50_ values of 0.7 nM and 33 nM, respectively. Taken together, 2- or appropriate 2,4-disubstitution patterns govern potent sEH inhibition in this series. Overall, the preliminary SAR in this work indicated a new favorable structural feature at the right- and left-hand side of the compounds to achieve potent sEH inhibition. Another aspect of characterizing the compounds is determining their cytotoxic activity; for this purpose, we used monocytes due to their high expression of sEH. By applying **33** and **38** to monocytes and measuring the metabolic activity after 24 h, we could exclude cytotoxic activity up to concentrations of 1 μM (data not shown).

### 2.3 Molecular Modeling

**Figure 5:**
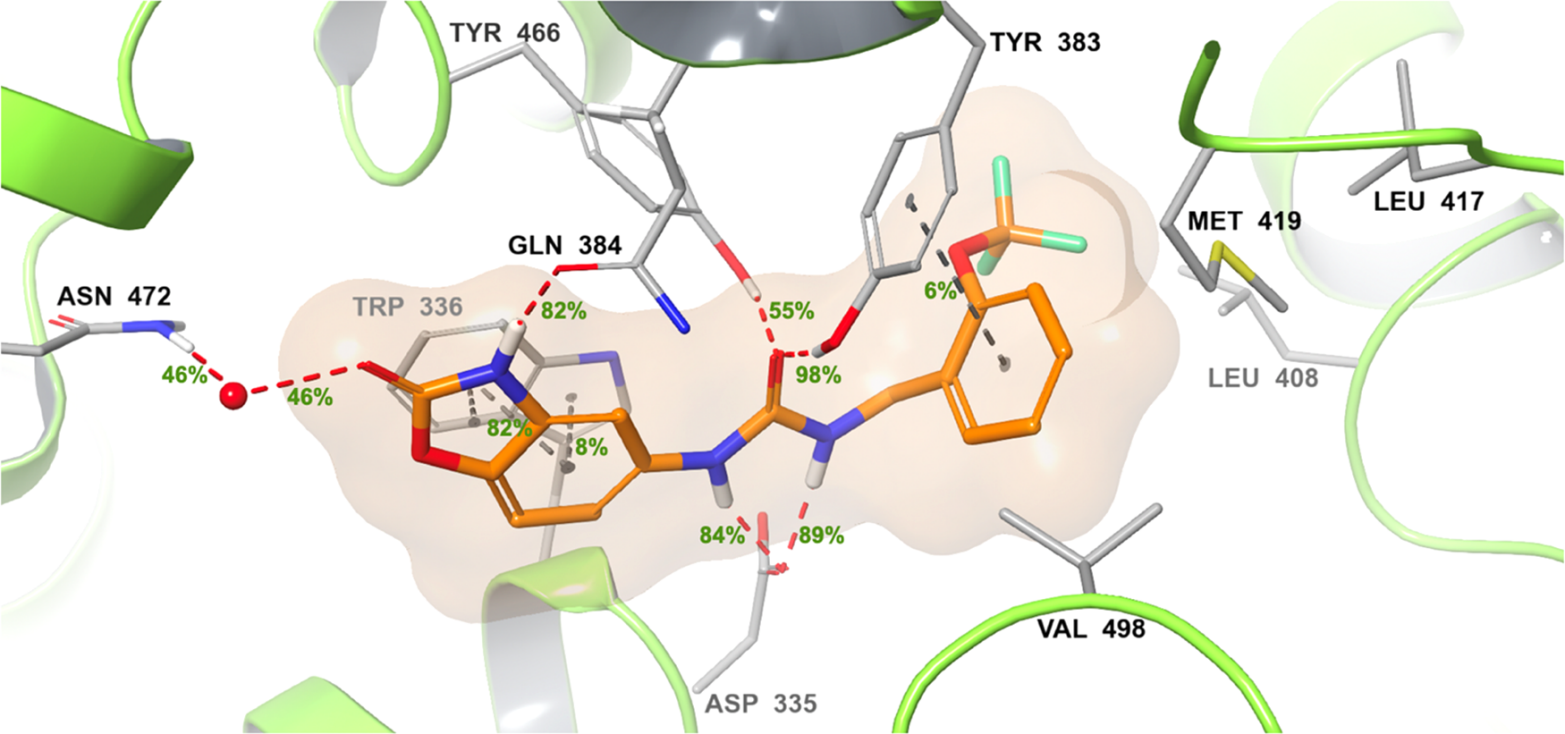
Protein–ligand interactions of compound **33** at sEH active site (PDB code: 4OCZ)^28^ with their occupancy values calculated during the simulation time of 200 ns.

In light of the rich structural information of the sEH active site in numerous co-crystal structures, we employed molecular docking studies along with 200 ns molecular dynamics (MD) simulations to exploit favorable binding interactions of the most potent inhibitor **33** (IC_50_ = 0.39 nM) within the sEH active site (PDB code: 4OCZ) (Figure 5).^28^ As expected, **33** binds to the catalytic triad through its urea moiety in the same manner as indicated for disubstituted urea-type inhibitors.^13^ These interactions between the catalytic triad and the urea group are a highly conserved feature across the reported sEH inhibitors, which anchor the inhibitor in the bottleneck of the L-shaped binding site. Accordingly, the urea oxygen forms strong H-bonds by accepting protons from Tyr383 and Tyr466 (98 and 55%, respectively), while the NH groups donate their hydrogens to establish stable H-bonding interactions with Asp335 (89 and 84%). Furthermore, the trifluoromethoxybenzyl group is located in a hydrophobic interior defined by Met419, Leu408, Leu417, Val418, Phe429, and Phe387 residues, which is often targeted by inhibitors to provide good binding affinity in the short branch.^14^ Moreover, a closer inspection of the MD simulations indicated the flexibility of the benzyl ring in which it may neatly align its π–π stacking orientation to the phenyl ring of Tyr466, also highlighting the significance of an aromatic ring in this region. Furthermore, the benzoxazolone ring of **33** binds close to the bottleneck of the active site toward the long branch, establishing stable π–π interactions with the Trp336 (82%), and the binding is further stabilized by an additional H-bonding interaction between the benzoxazolone NH and the amide oxygen of the nearby Gln384 residue (82%), a distinct polar interaction specific to individual crystal ligands as well as fragments in the long branch.^12,13,15^ In addition, benzoxazolone C=O undertakes a water-bridged H-bond with the side chain NH_2_ of Asn472, which altogether could explain the potent inhibition profile of **33** toward sEH.

## 3 Conclusions

The revealed structural features of diverse sets of sEH inhibitors indicate that they anchor to the active site with their urea or amide groups as primary pharmacophores, which form strong H-bonds with the catalytic residues at the bottleneck of the L-shaped active region. However, pendant groups to the amide/urea pharmacophores seldom establish polar interactions at the long and short internal branches but stabilize the inhibitor binding by mainly hydrophobic and space-filling properties.^4,30^ Nevertheless, X-ray crystallographic fragment screening studies disclosed several heteroaryl fragments with a single donor–acceptor pair functionality that form specific H-bonding interactions at both branch cavities.^12,13,15^ Therefore, we followed this evidence to introduce the benzoxazolone ring incorporating H-bond donor/acceptor features to the urea core to design new inhibitor chemotypes. As a result, the benzoxazolone template produced high efficiency in sEH inhibition by establishing pronounced H-bond and aromatic interactions in the long branch, which mimicked the similar interactions of reported crystal fragments and co-crystal ligands. Moreover, the *o*-substituted benzyl at the right-hand side was also crucial for directing the inhibitory activity and can be further exploited to optimize and strengthen the determined hydrophobic and/or van der Waals interactions.

In conclusion, the benzoxazolone function can be considered as a secondary pharmacophore to the central primary urea, warranting further research to validate its potential application in the design of improved sEH inhibitors. Moreover, **33** can be regarded as a new lead compound for the development of candidate compounds with improved potency and drug-like properties.

## 4 Experimental Section

### 4.1 Chemistry

All starting materials, reagents, and solvents were obtained from Sigma-Aldrich, Merck, and BLDpharm. The reaction was monitored with TLC on silica gel plates (Merck) and visualized under UV. A Biotage initiator + microwave apparatus was used for microwave irradiation reactions. Flash chromatography was performed on Redisep Gold silica gel columns (12 and 24 g) using Reveleris PREP Purification System (Buchi, New Castle, DE). High-resolution mass spectroscopy (HRMS) analysis was carried out using Waters LCT Premier XE mass spectrometer (high-sensitivity orthogonal acceleration time-of-flight) operating in ESI (+) method. The analysis was carried out in accordance with a UPLC-MS method using (A) water + 0.1% formic acid and (B) acetonitrile + 0.1% formic acid at a flow rate of 0.3 mL/min on an Aquity BEH C18 column (2.1 mm × 100 mm, 1.7 mm). Melting points were determined by an SMP50 automated melting point apparatus (Stuart, Staffordshire, ST15 OSA, U.K.). ^1^H and ^13^C-NMR spectra were recorded in DMSO-*d*_6_ on a 400 MHz (Varian Mercury) and a 500 MHz (Bruker Avance Neo) NMR spectrometer, respectively, using tetramethylsilane as an internal standard. All chemical shifts were recorded as δ (ppm), and coupling constants are reported as Hertz. The purity of the final compounds is >97%. All intermediates’ (**14, 22–30**) experimental results are given in the Supporting Information.

#### 4.1.1 *N*-[4-(Benzyloxy)phenyl]-2-oxo-2,3-dihydro-1,3-benzoxazole-6-carboxamide **(12)**

To a solution of 4-benzyloxy aniline (111.2 mg, 0.56 mmol, 1 equiv) in DMF, DMAP (16.3 mg, 0.14 mmol, 0.25 equiv) was added, and the resulting mixture was stirred under an N_2_ atmosphere for 15 min. After that, compound **11** (100 mg, 0.56 mmol, 1 equiv) was added and stirred for another 15 min, which was then added EDC·HCl (128.4 mg, 0.67 mmol, 1.2 equiv) and left to stir overnight. The reaction mixture was poured onto water and partitioned between EtOAc. The collected organic layer was dried, filtered, and evaporated to dryness to give the crude, which was triturated in hexane and EtOAc. Yield: 51%. Mp > 300 °C. ^1^H-NMR (400 MHz, DMSO-*d*_6_): δ 5.06 (2H, s), 6.98 (2H, d, *J* = 8.8 Hz), 7.18 (1H, d, *J* = 8.0 Hz), 7.29–7.44 (5H, m), 7.63 (2H, d, *J* = 8.8 Hz), 7.80 (1H, d, *J* = 8.0 Hz), 7.86 (1H, s), 10.1 (1H, s), 11.9 (1H, bs); ^13^C-NMR (100 MHz, DMSO-*d*_6_): δ 69.3, 108.6, 109.3, 114.7, 121.9, 124.2, 127.7, 127.8, 128.4, 128.6, 132.4, 133.3, 137.2, 143.0, 154.5, 154.6, 164.2. HRMS (*m*/*z*) [M + H]^+^ calculated for C_21_H_17_N_2_O_4_: 361.1188; found: 361.1189.

#### 4.1.2 4-(Benzyloxy)-*N*-(2-oxo-2,3-dihydro-1,3-benzoxazol-6-yl)benzamide **(15)**

In a solution of 4-benzyloxybenzoic acid (108 mg, 0.47 mmol, 1 equiv) in DMF, DIEA (205 μL, 1.18 mmol, 2.5 equiv) was added. The resulting mixture was stirred at rt for 15 min under an N_2_ atmosphere. Then, EDC.HCl (109 mg, 0.56 mmol, 1.1 equiv) and HOBt (77 mg, 0.56 mmol, 1.1 equiv) were added, and the mixture was stirred for another 15 min. Compound **13** (85 mg, 0.56 mmol, 1.1 equiv) was added to the reaction mixture and left to stir overnight. The reaction mixture was poured onto ice water. The precipitated product was filtered and left to dry to give the crude, which was triturated in hexane and EtOAc. Yield: 31%. Mp 256.6–259 °C. ^1^H-NMR (400 MHz, DMSO-*d*_6_): δ 5.18 (2H, s), 7.12 (2H, d, *J* = 8.8 Hz), 7.20 (1H, d, *J* = 8.8 Hz), 7.3–7.40 (4H, m), 7.45 (2H, d, *J* = 7.2 Hz), 7.71 (1H, d, *J* = 2.0 Hz), 7.92 (2H, d, *J* = 8.8 Hz), 10.1 (1H, s), 11.6 (1H, s); ^13^C-NMR (100 MHz, DMSO-*d*_6_): δ 69.3, 102.3, 109.1, 113.6, 114.4, 127.0, 127.6, 127.8, 128.4, 129.4, 130.2, 135.5, 136.6, 139.2, 154.6, 160.9, 164.7. HRMS (*m*/*z*) [M + H]^+^ calculated for C_21_H_17_N_2_O_4_: 361.1188; found: 361.1178.

#### 4.1.3 *N*-(2-Oxo-2,3-dihydro-1,3-benzoxazol-6-yl)-4-phenoxybenzamide **(16)**

Prepared from **13** (84 mg, 0.56 mmol) and 4-phenoxybenzoic acid (100 mg, 0.47 mmol) under the same method that was used in compound **15**. The obtained crude was triturated in methanol and EtOAc. Yield: 63%. Mp 231.8–233 °C. ^1^H-NMR (400 MHz, DMSO-*d*_6_): δ 7.06–7.09 (4H, m), 7.18–7.23 (2H, m), 7.36 (1H, d, *J* = 8.8 Hz), 7.43 (2H, t, *J* = 8.0 Hz), 7.69 (1H, s), 7.96 (2H, d, *J* = 8.4 Hz), 10.2 (1H, s), 11.5 (1H, bs); ^13^C-NMR (100 MHz, DMSO-*d*_6_): δ 102.3, 109.4, 113.7, 117.4, 119.5, 124.4, 129.4, 129.9, 130.3, 135.5, 139.3, 154.7, 155.6, 159.7, 164.7. HRMS (*m*/*z*) [M + H]^+^ calculated for C_20_H_15_N_2_O_4_: 347.1032; found: 347.1029.

#### 4.1.4 4-(Benzyloxy)-*N*-(3-methyl-2-oxo-2,3-dihydro-1,3-benzoxazol-6-yl)benzamide **(17)**

Prepared from **14** (108 mg, 0.65 mmol) and 4-benzyloxybenzoic acid (125 mg, 0.55 mmol) under the same method that was used in compound **15**. The obtained crude was washed with hot methanol and THF. Yield: 33%. Mp 251.8–253.7 °C. ^1^H-NMR (400 MHz, DMSO-*d*_6_): δ 3.35 (3H, s), 5.21 (2H, s), 7.14 (2H, d, *J* = 8.8 Hz), 7.22 (1H, d, *J* = 8.4 Hz), 7.33–7.42 (3H, m), 7.47 (2H, d, *J* = 8.8 Hz), 7.54 (1H, dd, *J* = 8.4, 2.0 Hz), 7.86 (1H, d, *J* = 2.0 Hz), 7.94 (2H, d, *J* = 8.8 Hz), 10.2 (1H, s); ^13^C-NMR (100 MHz, DMSO-*d*_6_): δ 28.0, 69.4, 102.7, 108.6, 114.5, 115.9, 126.9, 127.5, 127.8, 127.9, 128.5, 129.5, 134.4, 136.6, 141.6, 154.2, 160.9, 164.8. HRMS (*m*/*z*) [M + H]^+^ calculated for C_22_H_19_N_2_O_4_: 375.1345; found: 373.1351.

#### 4.1.5 *N*-(3-Methyl-2-oxo-2,3-dihydro-1,3-benzoxazol-6-yl)-4-phenoxybenzamide **(18)**

Prepared from **14** (85 mg, 0.51 mmol) and 4-phenoxybenzoic acid (92 mg, 0.43 mmol) under the same method that was used in compound **15**. The obtained crude was triturated in hexane and EtOAc. Yield: 55%. Mp 229.1–231.1 °C. ^1^H-NMR (500 MHz, DMSO-*d*_6_): δ 3.36 (3H, s), 7.11 (4H, m), 7.22–7.25 (2H, m), 7.45 (2H, t, *J* = 8.0 Hz), 7.56 (1H, dd, *J* = 8.5, 1.5 Hz), 7.86 (1H, d, *J* = 1.5 Hz), 8.00 (2H, d, *J* = 8.5 Hz), 10.3 (1H, s); ^13^C-NMR (125 MHz, DMSO-*d*_6_): δ 28.5, 103.2, 109.1, 116.4, 117.9, 120.0, 124.9, 128.1, 129.8, 130.4, 130.7, 134.7, 142.1, 154.6, 156.0, 160.3, 165.1. HRMS (*m*/*z*) [M + H]^+^ calculated for C_21_H_17_N_2_O_4_: 361.1188; found: 361.1194.

#### 4.1.6 4-(Benzyloxy)-*N*-(2-oxo-2,3-dihydro-1,3-benzoxazol-5-yl)benzamide **(20)**

Prepared from **19** (118 mg, 0.78 mmol) and 4-benzyloxybenzoic acid (150 mg, 0.65 mmol) under the same method that was used in compound **15**. The obtained crude was washed with isopropanol and EtOAc. Yield: 61%. Mp 260.7–262.8 °C. ^1^H-NMR (500 MHz, DMSO-*d*_6_): δ 5.21 (2H, s), 7.15 (2H, d, *J* = 8.8 Hz), 7.24 (1H, d, *J* = 8.7 Hz), 7.33–7.43 (4H, m), 7.47 (2H, d, *J* = 7.2 Hz), 7.73 (1H, d, *J* = 2.0 Hz), 7.94 (2H, d, *J* = 8.8 Hz), 10.1 (1H, s), 11.6 (1H, bs); ^13^C-NMR (125 MHz, DMSO-*d*_6_): δ 69.9, 102.8, 109.7, 114.1, 115.0, 127.6, 128.2, 128.4, 128.9, 130.0, 130.7, 136.1, 137.1, 139.7, 155.2, 161.4, 165.3. HRMS (*m*/*z*) [M + H]^+^ calculated for C_21_H_17_N_2_O_4_: 361.1188; found: 361.1190.

#### 4.1.7 *N*-(2-Oxo-2,3-dihydro-1,3-benzoxazol-5-yl)-4-phenoxybenzamide **(21)**

Prepared from **19** (98 mg, 0.66 mmol) and 4-phenoxybenzoic acid (117 mg, 0.55 mmol) under the same method that was used in compound **15**. The obtained crude was washed with hexane and EtOAc. Yield: 55%. Mp 231–233 °C. ^1^H-NMR (400 MHz, DMSO-*d*_6_): δ 7.06–7.1 (4H, m), 7.18–7.23 (2H, m), 7.35 (1H, dd, *J* = 8.8, 1.6 Hz), 7.43 (2H, t, *J* = 8.0 Hz), 7.70 (1H, d, *J* = 1.6 Hz), 7.96 (2H, d, *J* = 8.8 Hz), 10.2 (1H, s), 11.6 (1H, s); ^13^C-NMR (100 MHz, DMSO-*d*_6_): δ 102.3, 109.4, 113.7, 117.4, 119.5, 124.4, 129.4, 129.9, 130.2, 130.3, 135.5, 139.4, 154.7, 155.6, 159.8, 164.7. HRMS (*m*/*z*) [M + H]^+^ calculated for C_20_H_15_N_2_O_4_: 347.1032; found: 347.1036.

#### 4.1.8 1-[(2-Chlorophenyl)methyl]-3-(2-oxo-2,3-dihydro-1,3-benzoxazol-5-yl)urea **(31)**

In a 2–5 mL microwave vial, compound **22** (300 mg, 1.27 mmol, 1 equiv), compound **19** (229 mg, 1.53 mmol, 1.2 equiv), and DIEA (220 μL, 1.27 mmol, 1 equiv) were combined in DMF. The vial was closed and heated 110 °C for 1 h under microwave irradiation. The reaction solution was diluted with 1N HCl solution and EtOAc. The combined organic layers were dried over anhydrous MgSO_4_ and evaporated under reduced pressure to give the crude, which was purified with flash chromatography using 100% DCM → 90% DCM in MeOH solvent system. Yield: 10%. Mp 242.5 °C (decomp). ^1^H-NMR (500 MHz, DMSO-*d*_6_): δ 4.36 (2H, d, *J* = 5.5 Hz), 6.63 (1H, t, *J* = 5.5 Hz), 6.84 (1H, d, *J* = 8.0 Hz), 7.12 (1H, d, *J* = 8.5 Hz), 7.28–7.47 (5H, m), 8.73 (1H, s), 11.5 (1H, bs); ^13^C-NMR (125 MHz, DMSO-*d*_6_): δ 41.2, 100.5, 109.9, 111.4, 127.7, 129.0, 129.3, 129.6, 130.9, 132.5, 137.2, 137.8, 138.3, 155.3, 155.6. HRMS (*m*/*z*) [M + H]^+^ calculated for C_15_H_13_N_3_O_3_Cl: 318.0645; found: 318.0648.

#### 4.1.9 3-(2-Oxo-2,3-dihydro-1,3-benzoxazol-5-yl)-1-{[2-(trifluoromethyl)phenyl]methyl}urea **(32)**

Prepared from **19** (200 mg, 1.34 mmol) and **23** (300 mg, 1.12 mmol) under the same method that was used in compound **31**. The obtained crude was purified with flash chromatography using 100% DCM → 90% DCM in MeOH solvent system. Yield: 21.3%. Mp 237.8 °C (decomp). ^1^H-NMR (400 MHz, DMSO-*d*_6_): δ 4.49 (2H, d, *J* = 5.6 Hz), 6.68 (1H, t, *J* = 5.6 Hz), 6.85 (1H, dd, *J* = 8.8, 2.0 Hz), 7.13 (1H, d, *J* = 8.8 Hz), 7.46–7.49 (2H, m), 7.59 (1H, d, *J* = 7.6 Hz), 7.67 (1H, d, *J* = 7.6 Hz), 7.72 (1H, d, *J* = 7.6 Hz), 8.81 (1H, s), 11.5 (1H, bs); ^13^C-NMR (100 MHz, DMSO-*d*_6_): δ 100.0, 109.5, 110.9, 124.5 (q, ^1^*J*_C–F_ = 272 Hz), 125.7 (q, ^3^*J*_C–F_ = 5.8 Hz), 126.0 (q, ^2^*J*_C–F_ = 30.1 Hz), 127.3, 128.7, 130.5, 132.7, 136.7, 137.8, 138.7, 154.8, 155.2. HRMS (*m*/*z*) [M + H]^+^ calculated for C_16_H_13_N_3_O_3_F_3_: 352.0909; found: 352.0924.

#### 4.1.10 3-(2-Oxo-2,3-dihydro-1,3-benzoxazol-5-yl)-1-{[2-(trifluoromethoxy)phenyl]methyl}urea **(33)**

Prepared from **19** (189 mg, 1.26 mmol) and **24** (300 mg, 1.05 mmol) under the same method that was used in compound **31**. The obtained crude was purified with flash chromatography using 100% DCM → 90% DCM in MeOH solvent system. Yield: 16.6%. Mp >300 °C. ^1^H-NMR (400 MHz, DMSO-*d*_6_): δ 4.34 (2H, d, *J* = 6.0 Hz), 6.59 (1H, t, *J* = 6.0 Hz), 6.80 (1H, dd, *J* = 8.4, 2.4 Hz), 7.10 (1H, d, *J* = 8.4 Hz), 7.34–7.45 (5H, m), 8.72 (1H, s), 11.4 (1H, bs); ^13^C-NMR (100 MHz, DMSO-*d*_6_): δ 37.8, 100.0, 109.4, 110.9, 120.3 (q, ^1^*J*_C–F_ = 255 Hz), 120.6, 127.5, 128.6, 129.3, 130.5, 132.8, 136.7, 137.8, 146.1, 154.8, 155.2. HRMS (*m*/*z*) [M + H]^+^ calculated for C_16_H_13_N_3_O_4_F_3_: 368.0858; found: 368.0869.

#### 4.1.11 1-[(3-Chlorophenyl)methyl]-3-(2-oxo-2,3-dihydro-1,3-benzoxazol-5-yl)urea (**34**)

Prepared from **19** (229 mg, 1.52 mmol) and 25 (300 mg, 1.27 mmol) under the same method that was used in compound **31**. The obtained crude was purified with flash chromatography using 100% DCM → 90% DCM in MeOH solvent system. Yield: 8.5%. Mp 244.5 °C (decomp). ^1^H-NMR (500 MHz, DMSO-*d*_6_ + D_2_O): δ 4.23 (2H, s), 6.59 (1H, dd, *J* = 8.2, 2.2 Hz), 6.68 (1H, d, *J* = 8.2 Hz), 6.88 (1H, d, *J* = 2.2 Hz), 7.22–7.24 (2H, m), 7.30–7.33 (2H, m); ^13^C-NMR (125 MHz, DMSO-*d*_6_ + D_2_O): δ 42.5, 104.7, 105.8, 108.4, 126.2, 126.9, 127.2, 130.6, 133.4, 134.6, 143.1, 143.9, 146.3, 156.6, 166.4. HRMS (*m*/*z*) [M + H]^+^ calculated for C_15_H_13_N_3_O_3_Cl: 318.0645; found: 318.0657.

#### 4.1.12 1-[(4-Fluorophenyl)methyl]-3-(2-oxo-2,3-dihydro-1,3-benzoxazol-5-yl)urea **(35)**

Prepared from **19** (102 mg, 0.67 mmol) and **26** (128 mg, 0.58 mmol) under the same method that was used in compound **31**. The obtained crude was purified with flash chromatography using 100% DCM → 90% DCM in MeOH solvent system. Yield: 17%. Mp 249.3 °C (decomp). ^1^H-NMR (400 MHz, DMSO-*d*_6_): δ 4.24 (2H, d, *J* = 6.0 Hz), 6.58 (1H, t, *J* = 6.0 Hz), 6.81 (1H, dd, *J* = 8.4, 2.0 Hz), 7.08–7.15 (3H, m), 7.29–7.33 (2H, m), 7.45 (1H, d, *J* = 2.0 Hz), 8.61 (1H, s), 11.5 (1H, bs); ^13^C-NMR (100 MHz, DMSO-*d*_6_): δ 42.0, 100.0, 109.4, 110.9, 115.0 (d, ^2^*J*_C–F_ = 21.1 Hz), 129.1 (d, ^3^*J*_C–F_ = 7.7 Hz), 130.4, 136.6 (d, ^4^*J*_C–F_ = 3.2 Hz), 136.8, 137.8, 154.8, 155.3, 161.1 (d, ^1^*J*_C–F_ = 340.5 Hz). HRMS (*m*/*z*) [M + H]^+^ calculated for C_15_H_13_N_3_O_3_F 302.0941; found: 302.0928.

#### 4.1.13 1-[(4-Chlorophenyl)methyl]-3-(2-oxo-2,3-dihydro-1,3-benzoxazol-5-yl)urea **(36)**

Prepared from **19** (153 mg, 1.02 mmol) and **27** (200 mg, 0.85 mmol) under the same method that was used in compound **31**. The obtained crude was purified with flash chromatography using 100% DCM → 90% DCM in MeOH solvent system. Yield: 13.4%. Mp 251 °C (decomp). ^1^H-NMR (400 MHz, DMSO-*d*_6_): δ 4.28 (2H, d, *J* = 6.4 Hz), 6.64 (1H, t, *J* = 6.0 Hz), 6.84 (1H, dd, *J* = 8.8, 2.0 Hz), 7.12 (1H, d, *J* = 8.8 Hz), 7.32 (2H, d, *J* = 8.4 Hz), 7.40 (2H, d, *J* = 8.4 Hz), 7.48 (1H, d, *J* = 2.0 Hz), 8.68 (1H, s), 11.5 (1H, bs); ^13^C-NMR (100 MHz, DMSO-*d*_6_): δ 42.1, 100.0, 109.4, 110.9, 128.2, 128.9, 130.5, 131.2, 136.8, 137.8, 139.6, 154.8, 155.3. HRMS (*m*/*z*) [M + H]^+^ calculated for C_15_H_13_N_3_O_3_Cl 318.0645; found: 318.0641.

#### 4.1.14 3-(2-Oxo-2,3-dihydro-1,3-benzoxazol-5-yl)-1-{[4-(trifluoromethyl)phenyl]methyl}urea **(37)**

Prepared from **19** (111 mg, 0.73 mmol) and **28** (165 mg, 0.61 mmol) under the same method that was used in compound **31**. The obtained crude was purified with flash chromatography using 100% DCM → 90% DCM in MeOH solvent system. Yield: 14%. Mp 213.6 °C (decomp). ^1^H-NMR (400 MHz, DMSO-*d*_6_): δ 4.37 (2H, d, *J* = 6.0 Hz), 6.73 (1H, t, *J* = 6.0 Hz), 6.85 (1H, dd, *J* = 8.4, 2.0 Hz), 7.12 (1H, d, *J* = 8.4 Hz), 7.46 (1H, d, *J* = 2.0 Hz), 7.50 (2H, d, *J* = 8.0 Hz), 7.69 (2H, d, *J* = 8.0 Hz), 8.74 (1H, s), 11.5 (1H, bs); ^13^C-NMR (100 MHz, DMSO-*d*_6_): δ 42.4, 100.1, 109.4, 111.0, 124.4 (q, ^1^*J*_C–F_ = 270 Hz), 125.2 (q, ^3^*J*_C–F_ = 3.8 Hz), 127.3 (q, ^2^*J*_C–F_ = 31.4 Hz), 127.7, 130.5, 136.7, 137.8, 145.5, 154.8, 155.4. HRMS (*m*/*z*) [M + H]^+^ calculated for C_16_H_13_N_3_O_3_F_3_ 352.0909; found: 352.0917.

#### 4.1.15 1-[(2,4-Dichlorophenyl)methyl]-3-(2-oxo-2,3-dihydro-1,3-benzoxazol-5-yl)urea **(38)**

Prepared from **19** (200 mg, 1.33 mmol) and **29** (300 mg, 1.11 mmol) under the same method that was used in compound **31**. The obtained crude was purified with flash chromatography using 100% DCM → 90% DCM in MeOH solvent system. Yield: 17.3%. Mp 260.9 °C (decomp). ^1^H-NMR (400 MHz, DMSO-*d*_6_): δ 4.37 (2H, d, *J* = 6.0 Hz), 6.65 (1H, t, *J* = 6.0 Hz), 6.81 (1H, dd, *J* = 8.4, 2.0 Hz), 7.10 (1H, d, *J* = 8.4 Hz), 7.35–7.44 (3H, m), 7.58 (1H, d, *J* = 2.0 Hz), 8.76 (1H, s), 11.5 (1H, bs); ^13^C-NMR (100 MHz, DMSO-*d*_6_): δ 40.4, 100.0, 109.4, 110.9, 127.4, 128.5, 130.1, 130.5, 132.1, 132.8, 136.6, 136.7, 137.8, 154.8, 155.2. HRMS (*m*/*z*) [M + H]^+^ calculated for C_15_H_12_N_3_O_3_Cl_2_ 352.0256; found: 352.0259.

#### 4.1.16 1-[(2,3-Difluorophenyl)methyl]-3-(2-oxo-2,3-dihydro-1,3-benzoxazol-5-yl)urea **(39)**

Prepared from **19** (228 mg, 1.52 mmol) and **30** (300 mg, 1.26 mmol) under the same conditions that were used in compound **31**. The obtained crude was purified with flash chromatography using 100% DCM → 90% DCM in MeOH solvent system. Yield: 5.5%. Mp 259.9 °C (decomp). ^1^H-NMR (400 MHz, DMSO-*d*_6_): δ 4.35 (2H, d, *J* = 6.0 Hz), 6.65 (1H, t, *J* = 6.0 Hz), 6.81 (1H, dd, *J* = 8.4, 2.4 Hz), 7.1 (1H, d, *J* = 8.4 Hz), 7.15–7.17 (2H, m), 7.28–7.32 (1H, m), 7.43 (1H, d, *J* = 2.4 Hz), 8.69 (1H, s), 11.5 (1H, bs); ^13^C-NMR (100 MHz, DMSO-*d*_6_): δ 36.5, 100.1, 109.4, 110.9, 115.8 (d, ^3^*J*_C–F_ = 17.3 Hz), 124.5 (dd, ^2^*J*_C–F_ = *3.1 Hz*, ^3^*J*_C–F_ = 3.0 Hz), 124.6 (dd, ^2^*J*_C–F_ = 7.0 Hz, ^3^*J*_C–F_ = 4.5 Hz), 129.8 (d, ^3^*J*_C–F_ = 11.6 Hz), 130.4, 136.7, 137.8, 147.7 (dd, ^1^*J*_C–F_ = 243.6, ^2^*J*_C–F_ = 12.9), 149.9 (dd, ^1^*J*_C–F_ = 243.7, ^2^*J*_C–F_ = 12.1), 154.8, 155.2. HRMS (*m*/*z*) [M + H]^+^ calculated for C_15_H_12_N_3_O_3_F_2_ 320.0847; found: 320.0847.

### 4.2 Biology

#### 4.2.1 Determination of Soluble Epoxide Hydrolase (sEH) Activity

Human recombinant sEH was expressed and purified as published before.^31^ Briefly, Sf9 cells were infected with a recombinant baculovirus (obtained from Dr. Bruce Hammock, University of California, Davis, CA). Following 72 h of transfection, the cells were centrifuged and then sonicated (3 × 10 s at 4 °C) in lysis buffer comprising glycerol (10%), NaHPO_4_ (50 mM, pH 8), leupeptin (10 μg/mL), NaCl (300 mM), phenylmethanesulfonyl fluoride (1 mM), EDTA (1 mM), and soybean trypsin inhibitor (60 μg/mL). After centrifugation at 100,000*g* (60 min, 4 °C), the obtained supernatants were subjected to a benzylthio-sepharose-affinity chromatography and eluted with 4-fluorochalcone oxide in PBS containing 1 mM DTT and 1 mM EDTA for purification of sEH. The enzyme was dialyzed and concentrated (Millipore Amicon-Ultra-15 centrifugal filter), and the solution was further assayed for total protein content by a protein detection kit (Bio-Rad Laboratories, Munich, Germany). The epoxide hydrolase activity was measured using a fluorescence-based assay, as reported before.^27^ In brief, sEH was diluted in Tris buffer (25 mM, pH 7) containing BSA (0.1 mg/mL) to a suitable enzyme concentration and pretreated with test compounds or 0.1% DMSO as vehicle for 10 min at room temperature. To start the reaction, 50 μM of the PHOME substrate (3-phenyl-cyano(6-methoxy-2-naphthalenyl)methyl ester-2-oxiraneacetic acid), a nonfluorescent compound that is enzymatically converted by sEH into fluorescent 6-methoxy-naphtaldehyde, was added at RT. The reaction was stopped by addition of ZnSO_4_ (200 mM) after 60 min, and fluorescence was measured (λ_em_ 465 nm, λ_ex_ 330 nm) by employing a NOVOstar microplate reader (BMG LABTECH GmbH, Ortenberg, Germany). If required, the potential fluorescence of test compounds was subtracted from the readout.

#### 4.2.2 Determination of Cytotoxic Activity

The cytotoxic activities of compounds were assessed as previously described.^29^ Briefly, monocytes were obtained from leukocyte concentrates procured from fresh peripheral blood of healthy adult male and female donors, which were provided by the Institute of Transfusion Medicine, University Hospital Jena, Germany. The experimental protocol was authorized by the University Hospital Jena Ethical Committee, and all methods were executed according to the applicable regulations and guidelines. Erythrocytes were sedimented by mixing the leukocyte concentrates with dextran (from Leuconostoc spp., MW ∼ 40,000, Sigma-Aldrich), and then the supernatant was centrifuged on lymphocyte separation medium (Histopaque-1077, Sigma-Aldrich) to isolate different cell fractions. Then, the obtained peripheral blood mononuclear cell (PBMC) fraction was seeded in RPMI 1640 (Sigma-Aldrich) containing 10% (v/v) heat-inactivated fetal calf serum (FCS), 100 U/mL penicillin, and 100 μg/mL streptomycin in cell culture flasks (Greiner Bio-one, Frickenhausen, Germany) and incubated for 1.5 h at 37 °C and 5% CO_2_ for adherence of monocytes. Isolated monocytes (2 × 10^5^), which were obtained by scraping, were treated in a 96-well plate in RPMI 1640 containing 10% (v/v) heat-inactivated FCS, 100 U/mL penicillin, and 100 μg/mL streptomycin with different concentrations of **33** and **38** or 0.1% vehicle (DMSO) for 24 h. To determine cytotoxic activities of test compounds, after incubation of cells with 3-(4,5-dimethylthiazol-2-yl)-2,5-diphenyltetrazolium bromide (MTT, 5 mg/mL, 20 μL; Sigma-Aldrich, Munich, Germany) in the dark for 2–3 h at 37 °C and 5% CO_2_, the formazan product was solubilized in sodium dodecyl sulfate (SDS, 10% in 20 mM HCl). The absorbance was determined at 570 nm using a Multiskan Spectrum microplate reader (Thermo Fisher Scientific, Schwerte, Germany).

### 4.3 Molecular Modeling

A docking method was used to find potential binding positions for compound 33 inside the sEH active site’s hydrolase domain (PDB code 4OCZ).^28^ The recently published crystal structure was chosen since it had a good resolution (2.94 Å). The ligand was removed from the binding site and re-docked with great similarity to the same region. By contrasting the co-crystallized and docked poses, the RMSD value on heavy ligand atoms of the co-crystallized ligand of the chosen crystal structure was discovered to be 0.25. Maestro was used to draw the structure.^32^ Using the OPLS4 forcefield, the atom types and protonation states of ligands and proteins were assigned at pH 7.0 ± 2.0. The ligands were prepared using the LigPrep^33^ routine, and the enzyme and the expected sites of its missing side chains were added using Protein Preparation Wizard.^34^ Both the partial charge cutoff value and the van der Waals radius scaling factor were utilized with their default values of 1.0 and 0.25. Only the poses with the best scores were kept for visualization and future research after the Glide^35,36^ simulations were completed. Top-scoring poses were given to molecular dynamics throughout 200 ns simulations with four copies to observe time-dependent patterns of interaction of the molecules. System Builder was used to set up the simulation systems, and Desmond^37^ was used to conduct the simulations. For waters, the SPC model was utilized. 6 Na+ ions were used to neutralize the systems. OPLS4 forcefield was used to prepare the simulation system.

#### 4.3.1 Molecular Dynamics Simulations

The simulations were relaxed with five stages for the simulations carried out using sEH. (i) NVT ensemble and Brownian dynamics were used in the first stage. The temperature was 10 K for 12 ps, the relaxation process included 2000 minimization steps, and the presence of harmonic constraints on the solute atoms was verified by applying a force constant of 50 kcal/mol^–2^. (ii) The NVT ensemble and Langevin technique were used in the second stage to continue with the same parameters, and (iii) the NPT ensemble and Langevin method were used in the third stage. (iv) The temperature of the preceding system was raised to the simulated temperature at the fourth stage (300 K). (v) Restraints were not used throughout the system’s last and fifth relaxation step, which lasted for 24 ps. The recording interval was then set to 200 ps to begin the MD simulation, which would last for 200 ns. The Desmond Trajectory Clustering panel chose the trajectory with the most populated on it. The occupancy values from the second simulation are reported since the molecular dynamics of each complex demonstrated consistent binding patterns across all simulation copies.

## Supporting Information Available

NMR spectra of the compounds, RMSD plots, and cytotoxicity data (PDF)
