# Supplementary material for: Benzoxazolone-5-Urea Derivatives as Human Soluble Epoxide Hydrolase (sEH) Inhibitors
Source: ACS Omega. 2023 Jan 4;8(2):2445–54. doi: 10.1021/acsomega.2c06936 (PMC9850727; doi:10.1021/acsomega.2c06936)
Supplement: Supplementary file 1 — ao2c06936_si_001.pdf [file ao2c06936_si_001.pdf]

Supplementary Material

Benzoxazolone-5-urea Derivatives as Human Soluble Epoxide Hydrolase (sEH) Inhibitors

Tugce Gur Maz,[¥] Beyzanur Koc,[¥] Paul M. Jordan,[†] Kübra İbiş,[¥] Burcu Çalışkan,[¥] Oliver Werz,[†] Erden Banoglu[¥,*]

[¥]Department of Pharmaceutical Chemistry, Faculty of Pharmacy, Gazi University, Taç Sok. No:3 Yenimahalle, 06560 Ankara, Turkey
[†]Department of Pharmaceutical/Medicinal Chemistry, Institute of Pharmacy, Friedrich Schiller University Jena, Philosophenweg 14, D-7743 Jena, Germany

Corresponding author: Erden Banoglu

banoglu@gazi.edu.tr

Department of Pharmaceutical Chemistry
Faculty of Pharmacy, Gazi University
Taç Sok. No:3 Yenimahalle, 06560 Ankara, Turkey

Contents

Synthesis of Intermediate 14 3
Synthesis of Intermediate 22-30 3
Figure S1. ^{1}H-NMR Spectra of 12 5
Figure S2. ^{13}C-NMR Spectra of 12 6
Figure S3. HRMS Spectra of 12 7
Figure S4. ^{1}H-NMR Spectra of 15 8
Figure S5. ^{13}C-NMR Spectra of 15 9
Figure S6. HRMS Spectra of 15 10
Figure S7. ^{1}H-NMR Spectra of 16 11
Figure S8. ^{13}C-NMR Spectra of 16 12
Figure S9. HRMS Spectra of 16 13
Figure S10. ^{1}H-NMR Spectra of 17 14
Figure S11. ^{13}C-NMR Spectra of 17 15
Figure S12. HRMS Spectra of 17 16
Figure S13. ^{1}H-NMR Spectra of 18 17
Figure S14. ^{13}C-NMR Spectra of 18 18

Figure S15. HRMS Spectra of 18 19
Figure S16. ^{1}H-NMR Spectra of 20 20
Figure S17. ^{13}C-NMR Spectra of 20 21
Figure S18. HRMS Spectra of 20 22
Figure S19. ^{1}H-NMR Spectra of 21 23
Figure S20. ^{13}C-NMR Spectra of 21 24
Figure S21. HRMS Spectra of 21 25
Figure S22. ^{1}H-NMR Spectra of 31 26
Figure S23. ^{13}C-NMR Spectra of 31 27
Figure S24. HRMS Spectra of 31 28
Figure S25. ^{1}H-NMR Spectra of 32 29
Figure S26. ^{13}C-NMR Spectra of 32 30
Figure S27. HRMS Spectra of 32 31
Figure S28. ^{1}H-NMR Spectra of 33 32
Figure S29. ^{13}C-NMR Spectra of 33 33
Figure S30. HRMS Spectra of 33 34
Figure S31. ^{1}H-NMR Spectra of 34 35
Figure S32. ^{13}C-NMR Spectra of 34 36
Figure S33. HRMS Spectra of 34 37
Figure S34. ^{1}H-NMR Spectra of 35 38
Figure S35. ^{13}C-NMR Spectra of 35 39
Figure S36. HRMS Spectra of 35 40
Figure S37. ^{1}H-NMR Spectra of 36 41
Figure S38. ^{13}C-NMR Spectra of 36 42
Figure S39. HRMS Spectra of 36 43
Figure S40. ^{1}H-NMR Spectra of 37 44
Figure S41. ^{13}C-NMR Spectra of 37 45
Figure S42. HRMS Spectra of 37 46
Figure S43. ^{1}H-NMR Spectra of 38 47
Figure S44. ^{13}C-NMR Spectra of 38 48
Figure S45. HRMS Spectra of 38 49
Figure S46. ^{1}H-NMR Spectra of 39 50
Figure S47. ^{13}C-NMR Spectra of 39 51
Figure S48. HRMS Spectra of 39 52
Figure S49: The interactions observed between compound 33 and protein 53
Figure S50: The 2D schematic representation of compound 33 with color-coded rotatable bonds 53
Figure S51: RMSD plots of compound 33 simulated with sEH enzyme (PDB ID:4OCZ) for four MD simulations. 54
Figure S52: Effects of compound 33 and 38 on cell viability of human monocytes. 54

Synthesis of Intermediate 14

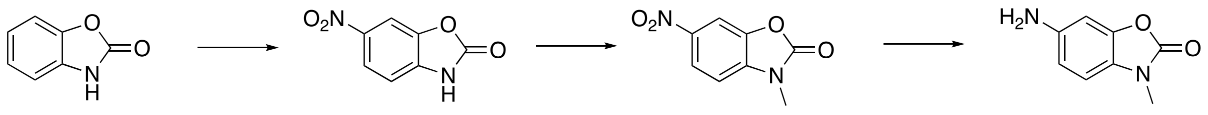

SI 1 SI 2 14

6-nitro-2,3-dihydro-1,3-benzoxazol-2-one (SI 1): To a solution of nitric acid (65%, 4 mL), 2,3-dihydro-1,3-benzoxazol-2-one (408 mg, 3.02 mmol) was added in an ice bath and left to stirr at 0 °C for an hour. Then it was taken to rt, and left to stirr for 3 hours. The mixture was poured onto ice-water and the precipitated product was filtered and dried to give the crude which was used without further purification for next step. CAS: 4694-91-1. Yield: 60%. Mp: 240.8-243 °C, 243-245 °C [1].

3-methyl-6-nitro-2,3-dihydro-1,3-benzoxazol-2-one (SI 2): In a solution of NaH (48.5 mg, 1.21 mmol, 1.1 eq) in THF, **SI 1** (200 mg, 1.1 mmol, 1 eq) was added on an ice bath. Then, dimethylsulfate (125 µL, 1.32 mmol, 1.2 eq) was added dropwise and stirred for 30 minutes at 0 °C. The resulting mixture was left to stirr overnight at rt. The next day, the mixture was poured onto ice and filtered to give the crude which was used without further purification for next step. CAS: 101084-61-1. Yield: 80%. Mp: 179.8-181.3 °C, 175-180 °C [2].

6-amino-3-methyl-2,3-dihydro-1,3-benzoxazol-2-one (14). To a solution of **SI 2** (353 mg, 1.81 mmol) in EtOAc, Pd/C (35 mg) was added. The resulting mixture was stirred overnight at rt under H_2 atmosphere. Next day, it was filtered from a pack of Celite. The filtrate was evaporated in vacuo to give the crude which was washed with EtOAc and hexane, then filtered. CAS: 99584-10-8. Yield: Quant. Mp: 148.2-150 °C, 151-153 °C [3].

Synthesis of Intermediate 22-30

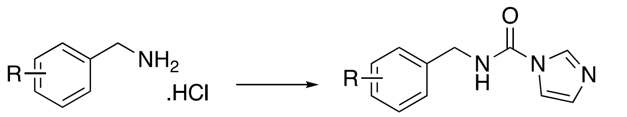

22-30

22 2-Cl
23 2-CF_3
24 2-OCF_3
25 3-Cl
26 4-F
27 4-Cl
28 4-CF_3
29 2,4-diCl
30 2,3-diF

To a solution of HCl salt of appropriate benzylamines (1 eq) in DMF, CDI (1.2 eq) was added on an ice bath, and left to stirr for 15 minutes. Then, it was taken to rt, and stirred for another 30 minutes. The reaction mixture was poured onto water, partitioned between EtOAc. The collected organic layer was dried, filtered and evaporated to dryness to give the crude CDI adducts of benzylamines in quantitative yield which was used without further purification for next step [4].

N-[(2-chlorophenyl)methyl]-1H-imidazole-1-carboxamide (22). HRMS (m/z) $[M+H]^+$ calculated for $C_{11}H_{11}N_3OCl$: 236.0591; found: 236.0596.

N-{[2-(trifluoromethyl)phenyl]methyl}-1H-imidazole-1-carboxamide (23). HRMS (m/z) $[M+H]^+$ calculated for $C_{12}H_{11}N_3O_2F_3$: 286.0803; found: 286.0807.

N-{[2-(trifluoromethoxy)phenyl]methyl}-1H-imidazole-1-carboxamide (24). HRMS (m/z) $[M+H]^+$ calculated for $C_{21}H_{17}N_2O_4$: 361.1188; found: 361.1189.

N-[(3-chlorophenyl)methyl]-1H-imidazole-1-carboxamide (25). HRMS (m/z) $[M+H]^+$ calculated for $C_{11}H_{11}N_3OCl$: 236.0591; found: 236.0600.

N-[(4-fluorophenyl)methyl]-1H-imidazole-1-carboxamide (26). HRMS (m/z) $[M+H]^+$ calculated for $C_{11}H_{11}N_3OF$: 220.0886; found: 220.0882.

N-[(4-chlorophenyl)methyl]-1H-imidazole-1-carboxamide (27). HRMS (m/z) $[M+H]^+$ calculated for $C_{11}H_{11}N_3OCl$: 236.0591; found: 236.0600.

N-{[4-(trifluoromethyl)phenyl]methyl}-1H-imidazole-1-carboxamide (28). HRMS (m/z) $[M+H]^+$ calculated for $C_{12}H_{11}N_3O_2F_3$: 286.0803; found: 286.0811.

N-[(2,4-dichlorophenyl)methyl]-1H-imidazole-1-carboxamide (29). HRMS (m/z) $[M+H]^+$ calculated for $C_{11}H_{10}N_3OCl_2$: 270.0201; found: 270.0208.

N-[(2,3-difluorophenyl)methyl]-1H-imidazole-1-carboxamide (30). HRMS (m/z) $[M+H]^+$ calculated for $C_{11}H_{10}N_3OF_2$: 236.0591; found: 236.0600.

Figure S1. ^{1}H-NMR Spectra of 12

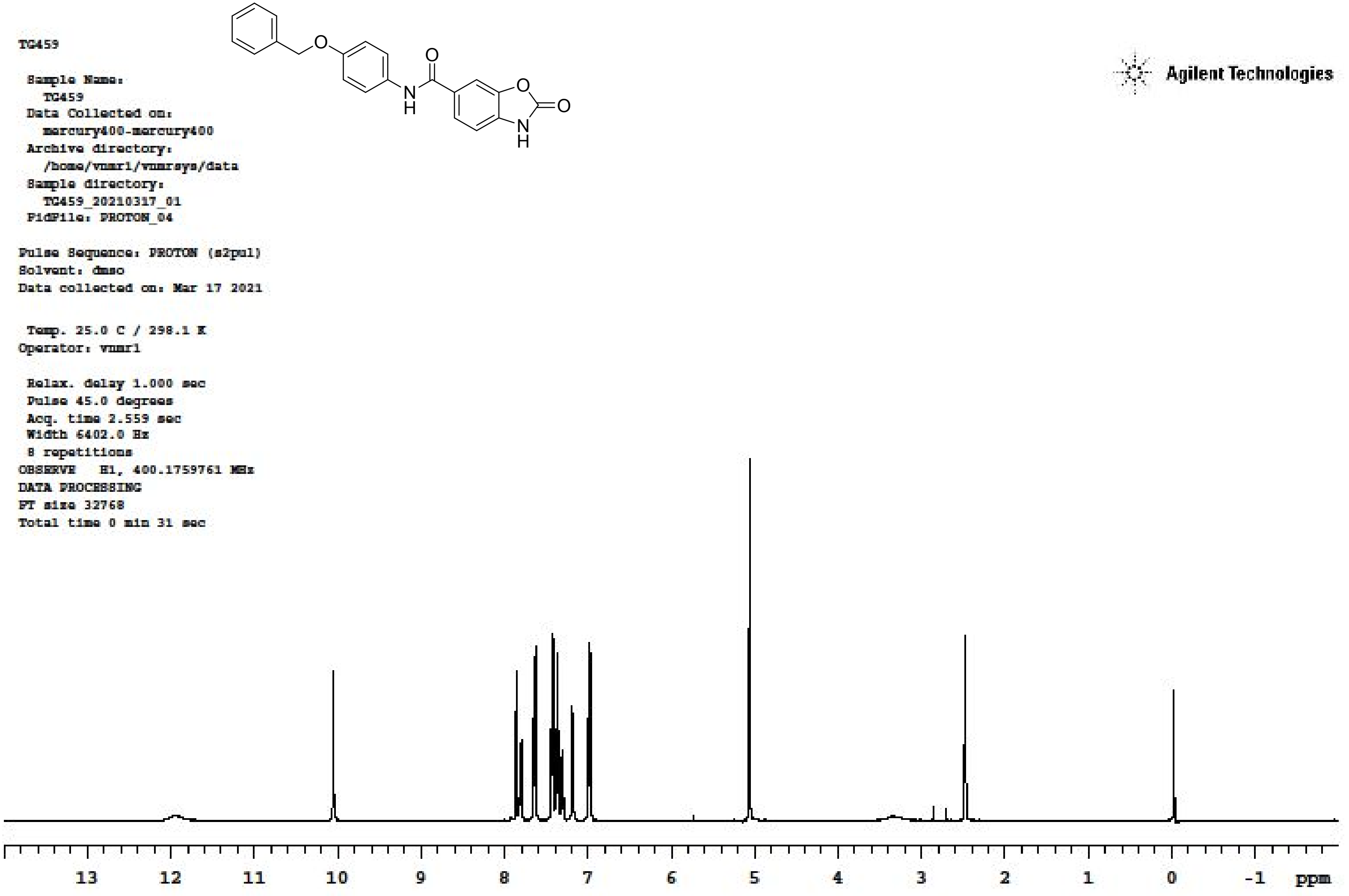

Figure S2. ^{13}C-NMR Spectra of 12

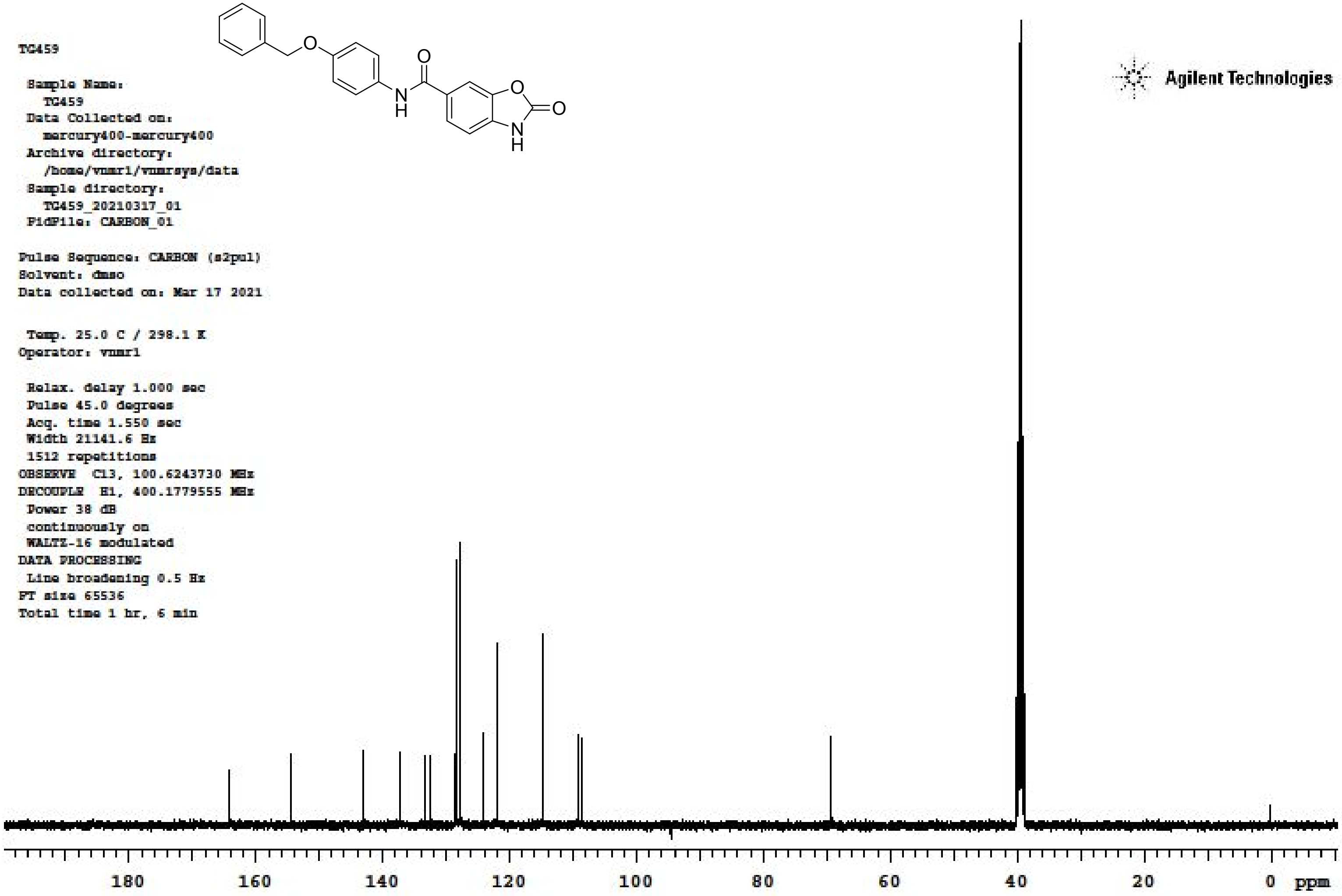

Figure S3. HRMS Spectra of 12

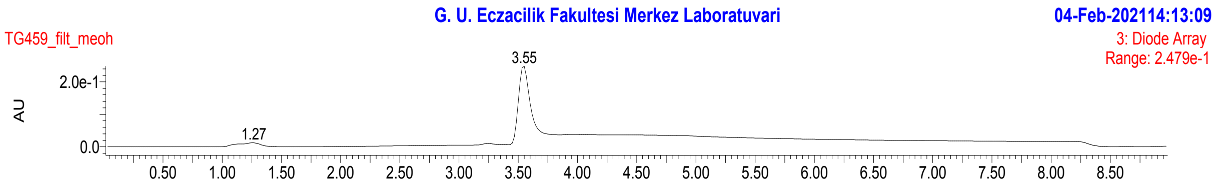

Single Mass Analysis
Tolerance = 5.0 PPM / DBE: min = -1.5, max = 50.0
Element prediction: Off
Number of isotope peaks used for i-FIT = 3
Monoisotopic Mass, Even Electron Ions
8 formula(e) evaluated with 1 results within limits (up to 50 closest results for each mass)
Elements Used:

Mass	Calc. Mass	mDa	PPM	DBE	Formula	i-FIT	i-FIT (Norm)	C	H	N	O
361.1189	361.1188	0.1	0.3	14.5	C21 H17 N2 O4	71.4	0.0	21	17	2	4

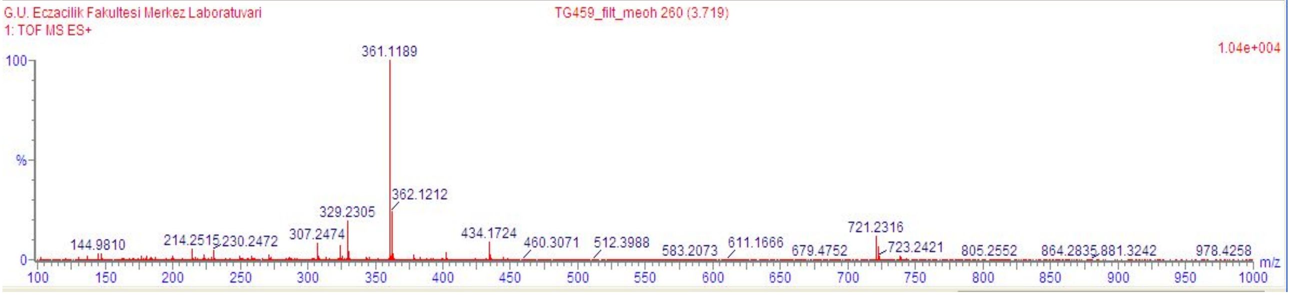

Figure S4. ¹H-NMR Spectra of 15

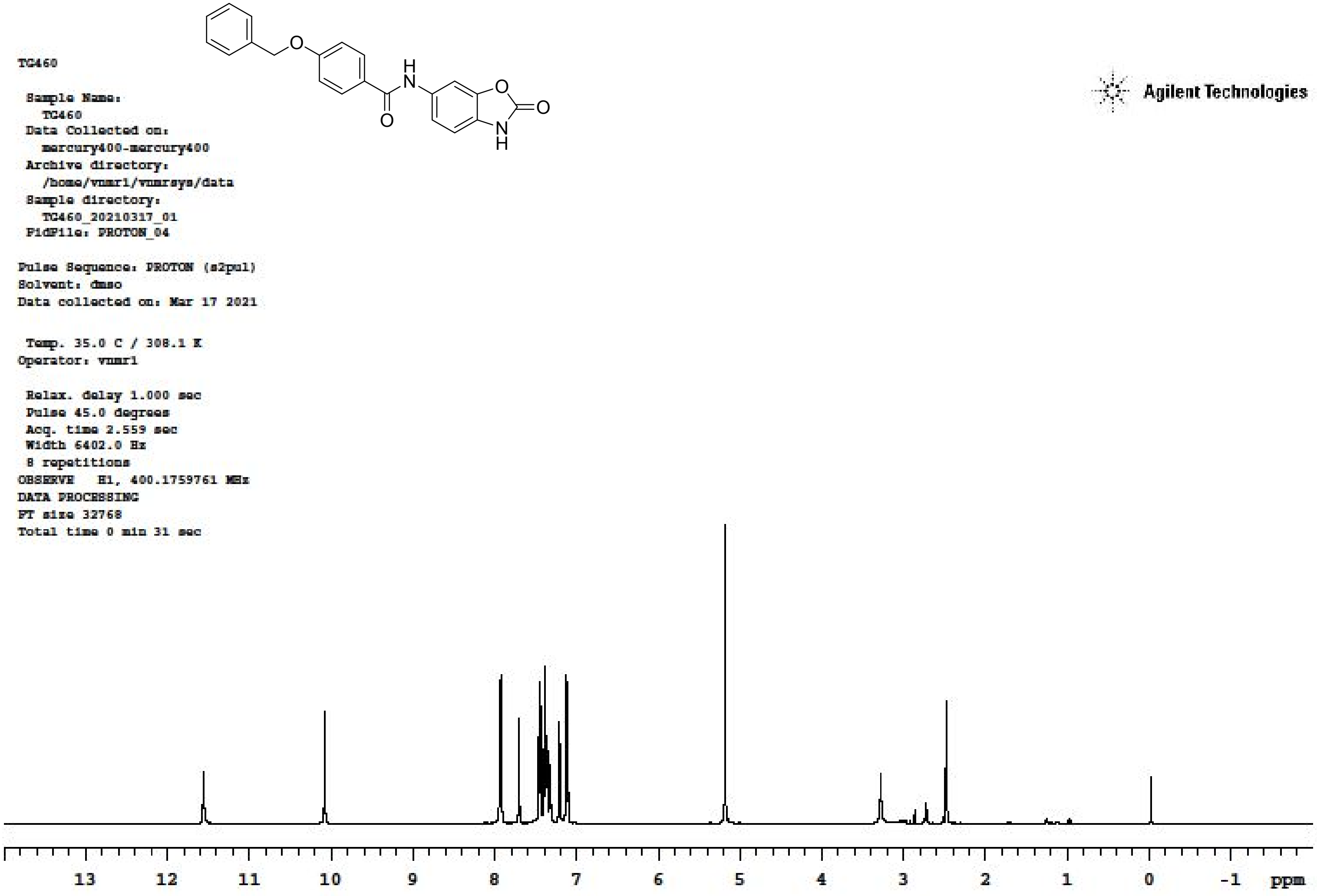

Figure S5. ^{13}C-NMR Spectra of 15

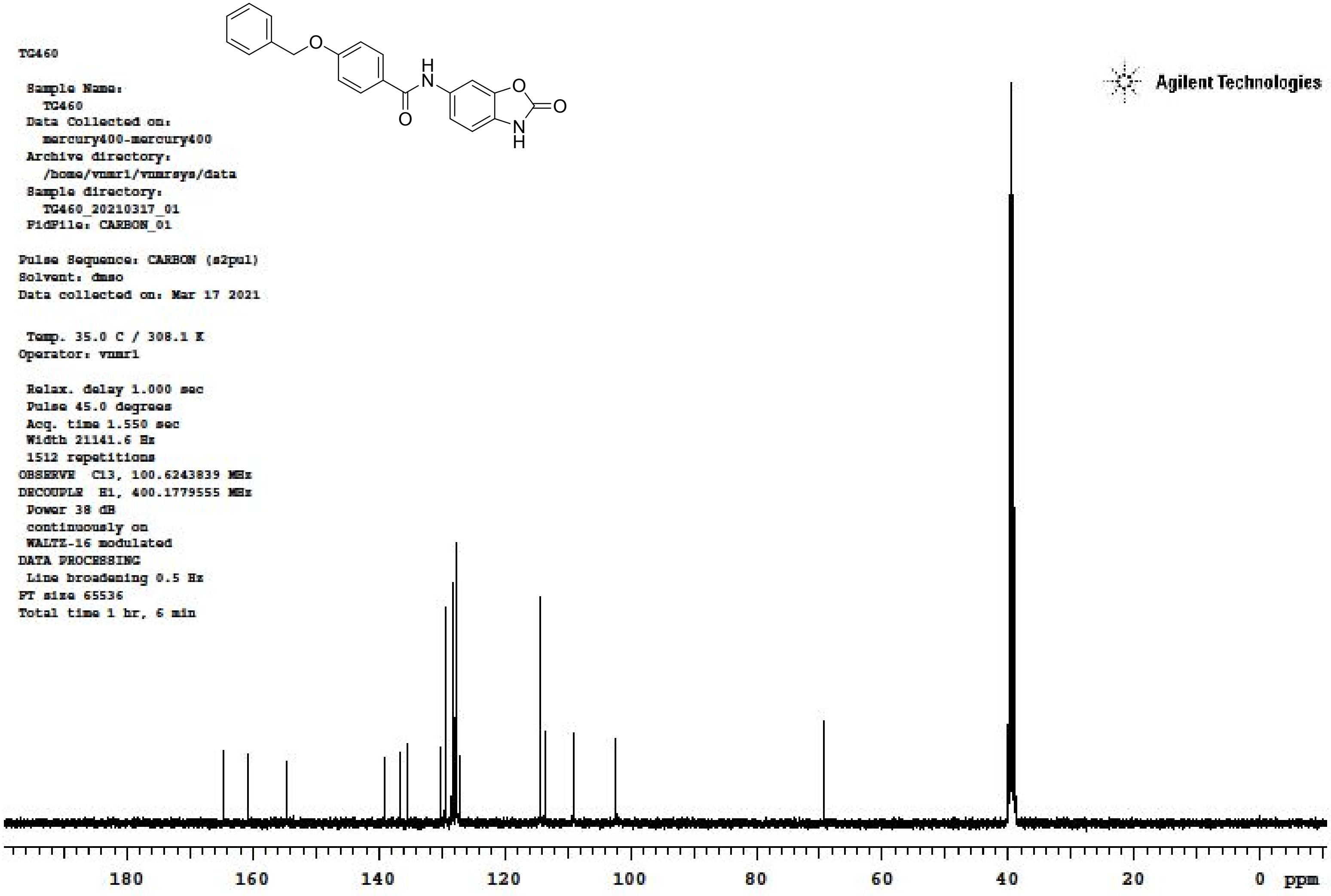

Figure S6. HRMS Spectra of 15

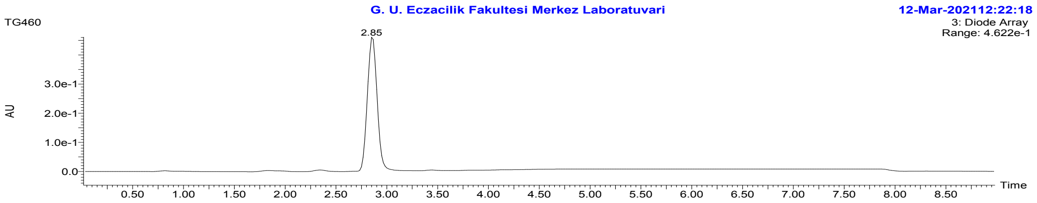

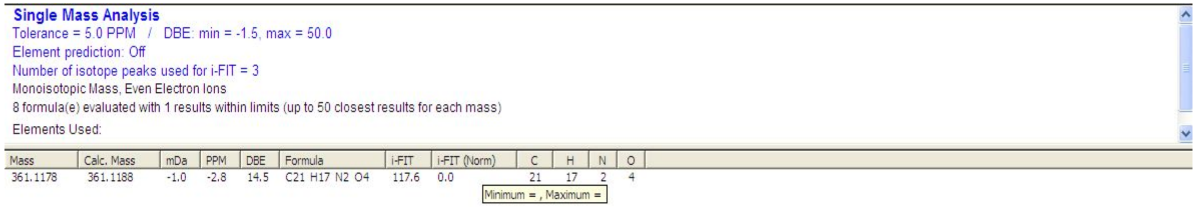

Single Mass Analysis

Tolerance = 5.0 PPM / DBE: min = -1.5, max = 50.0

Element prediction: Off

Number of isotope peaks used for i-FIT = 3

Monoisotopic Mass, Even Electron Ions

8 formula(e) evaluated with 1 results within limits (up to 50 closest results for each mass)

Elements Used:

Mass	Calc. Mass	mDa	PPM	DBE	Formula	i-FIT	i-FIT (Norm)	C	H	N	O
361.1178	361.1188	-1.0	-2.8	14.5	C21 H17 N2 O4	117.6	0.0	21	17	2	4

Minimum = , Maximum =

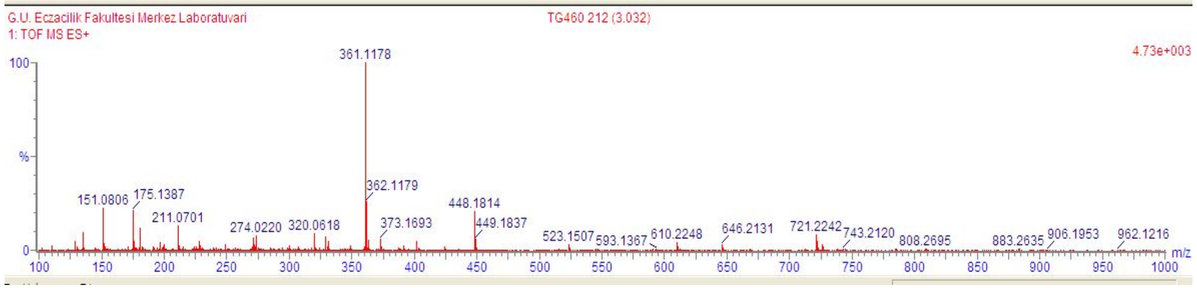

Figure S7. ¹H-NMR Spectra of 16

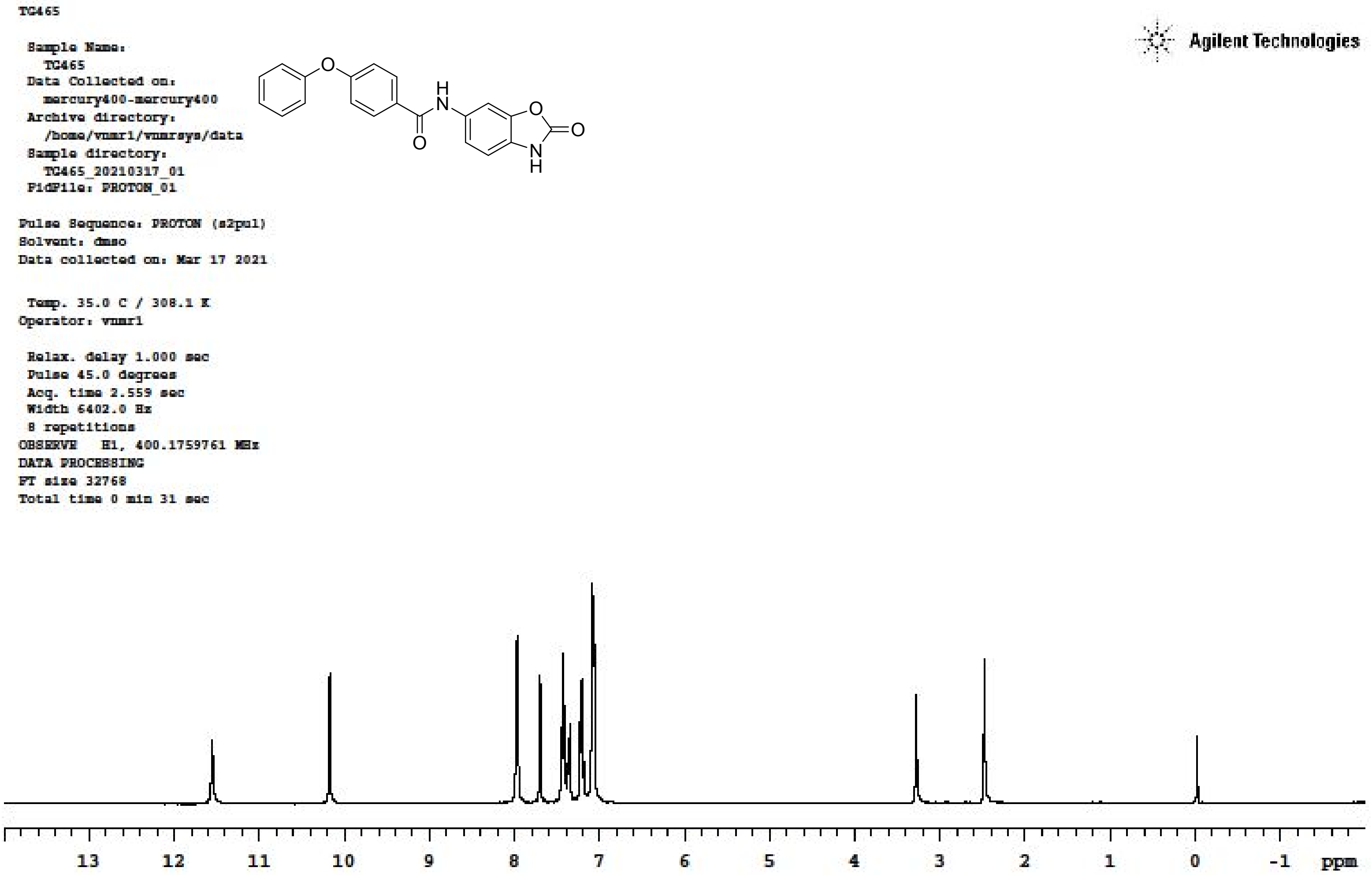

Figure S8. ^{13}C-NMR Spectra of 16

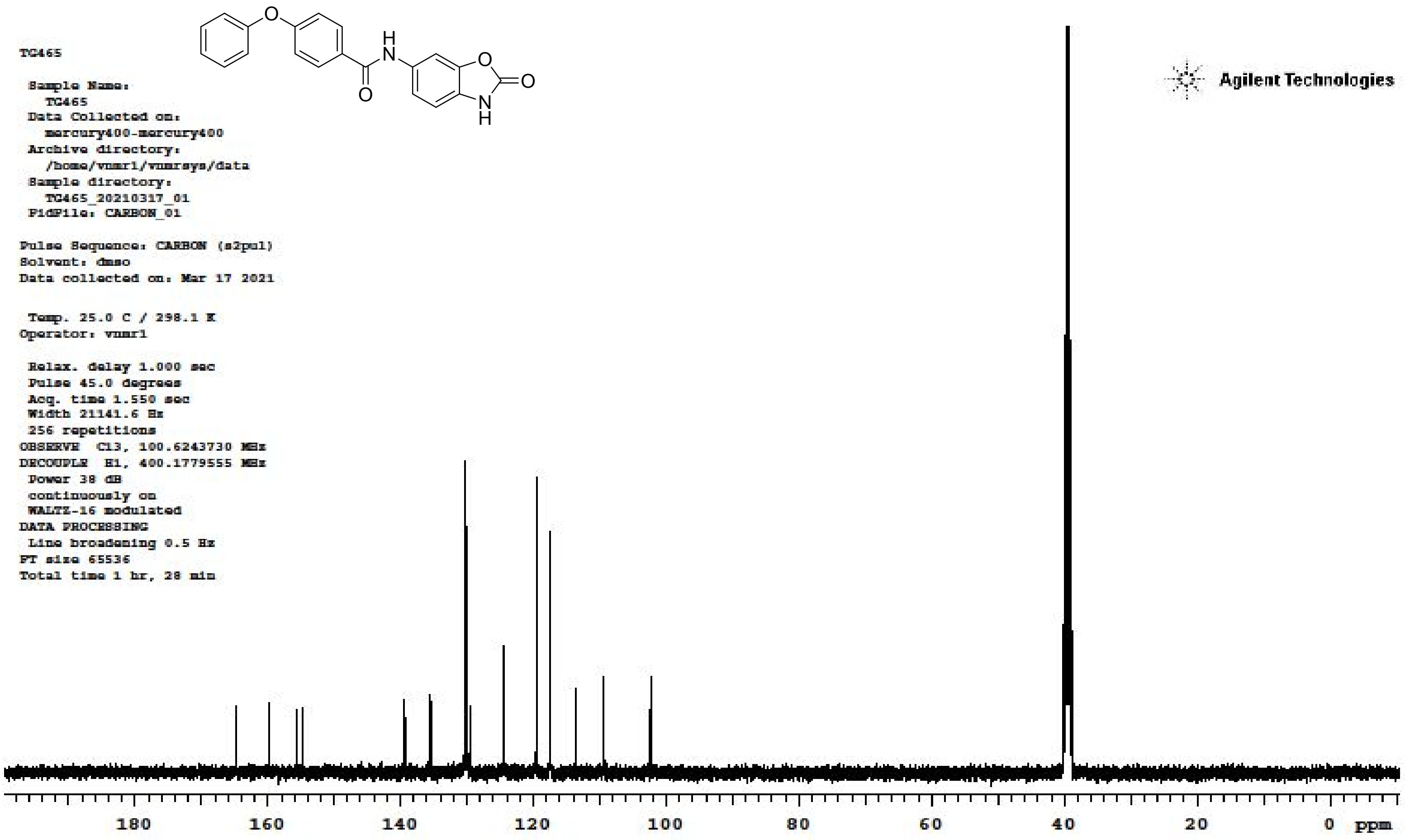

Figure S9. HRMS Spectra of 16

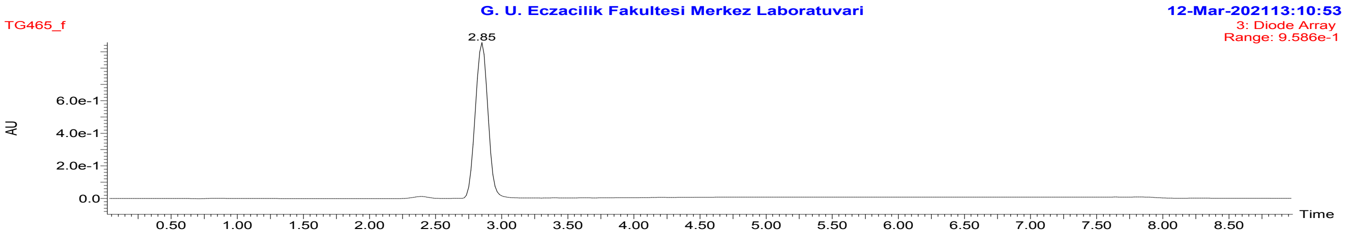

Single Mass Analysis

Tolerance = 5.0 PPM / DBE: min = -1.5, max = 50.0

Element prediction: Off

Number of isotope peaks used for i-FIT = 3

Monoisotopic Mass, Even Electron Ions

17 formula(e) evaluated with 1 results within limits (up to 50 closest results for each mass)

Elements Used:

Mass	Calc. Mass	mDa	PPM	DBE	Formula	i-FIT	i-FIT (Norm)	C	H	N	O
347.1029	347.1032	-0.3	-0.9	14.5	C20 H15 N2 O4	262.1	0.0	20	15	2	4

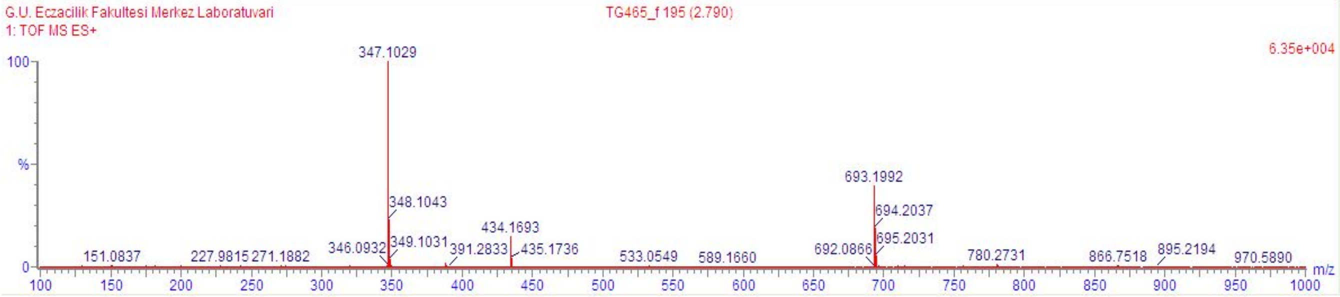

Figure S10. ^{1}H-NMR Spectra of 17

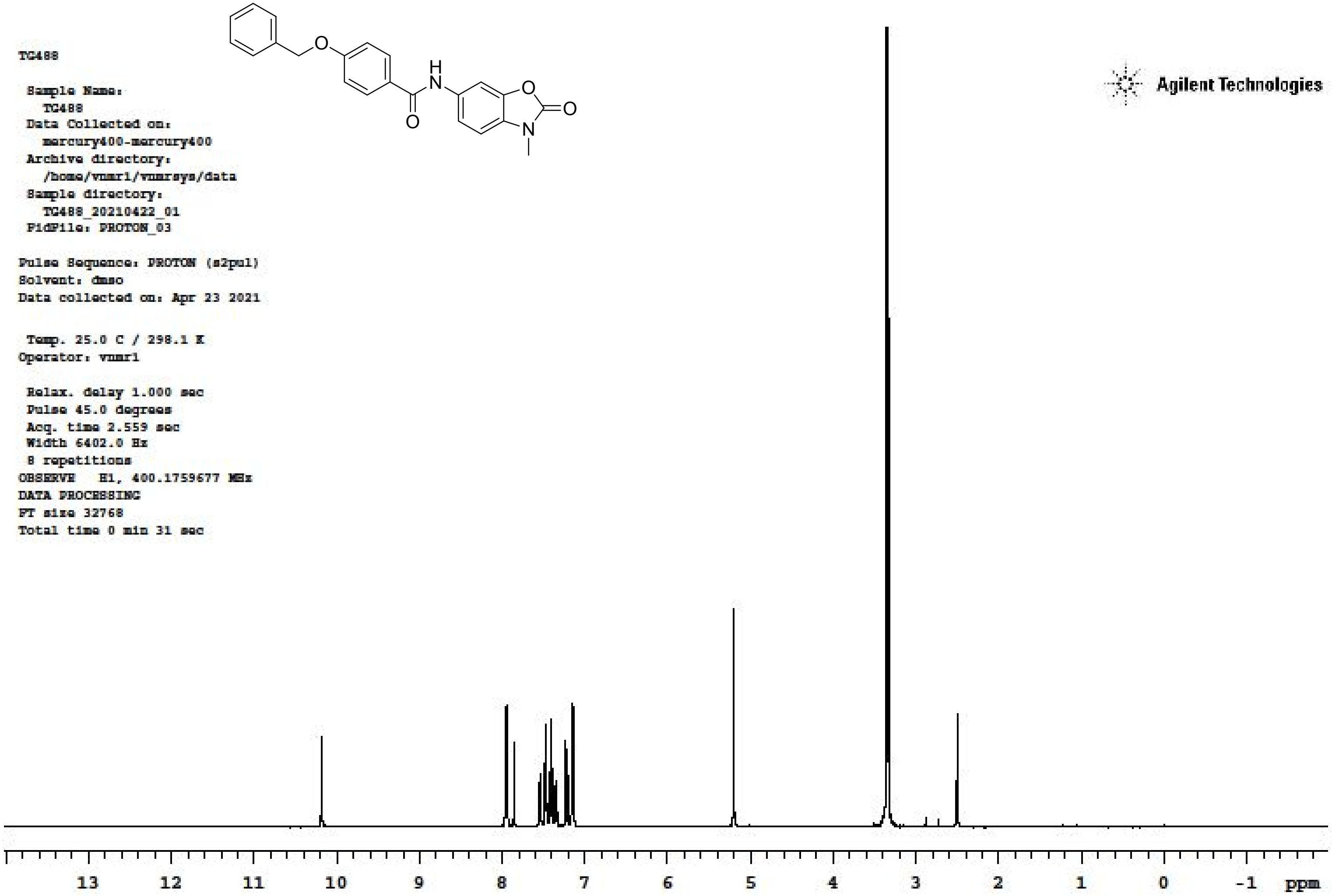

Figure S11. ^{13}C-NMR Spectra of 17

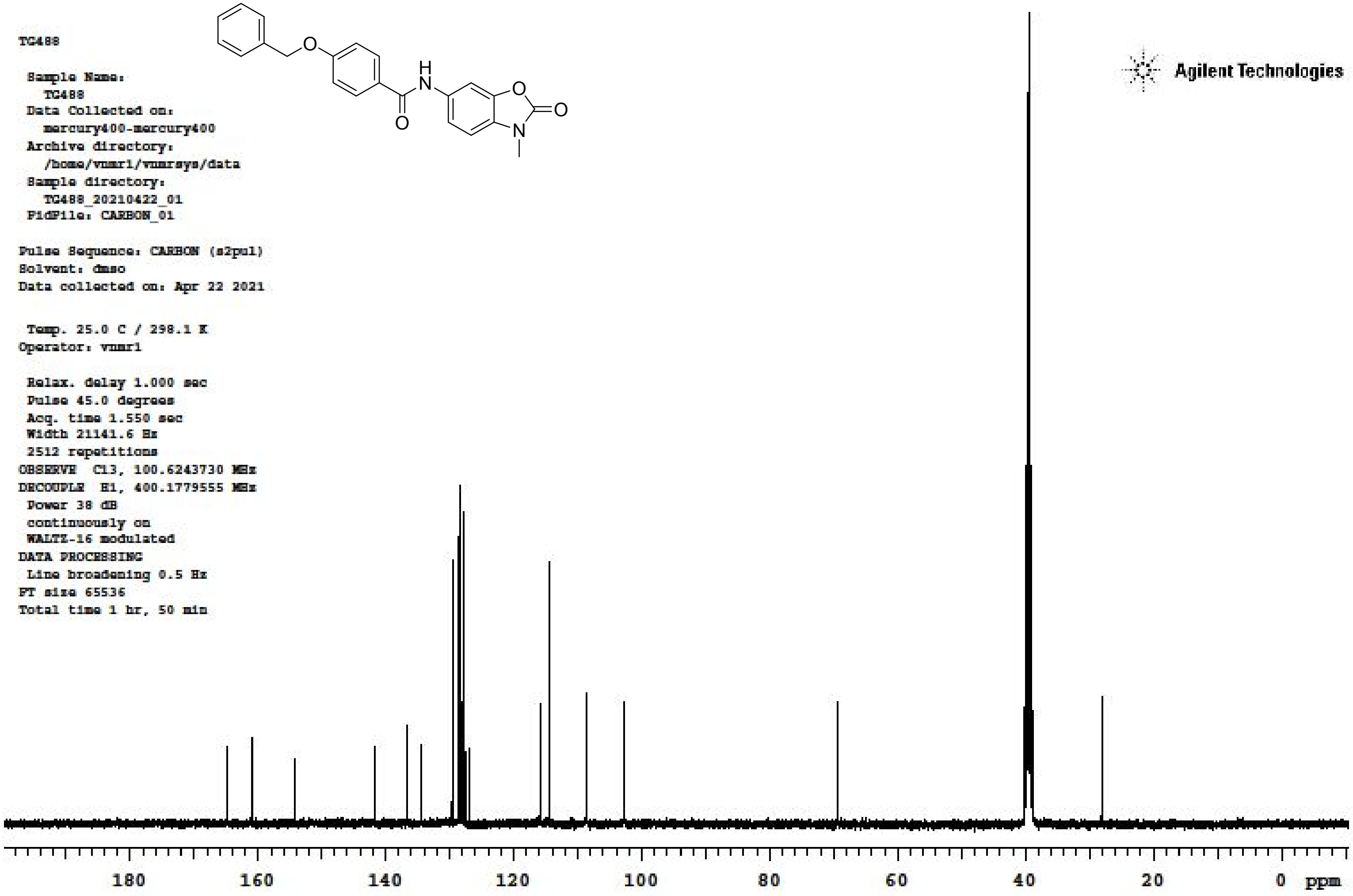

Figure S12. HRMS Spectra of 17

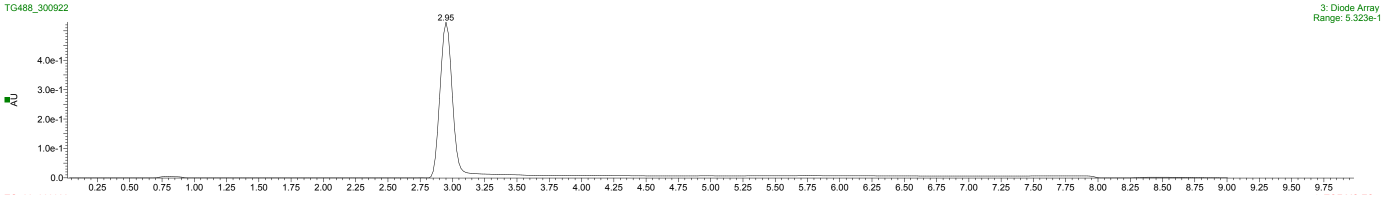

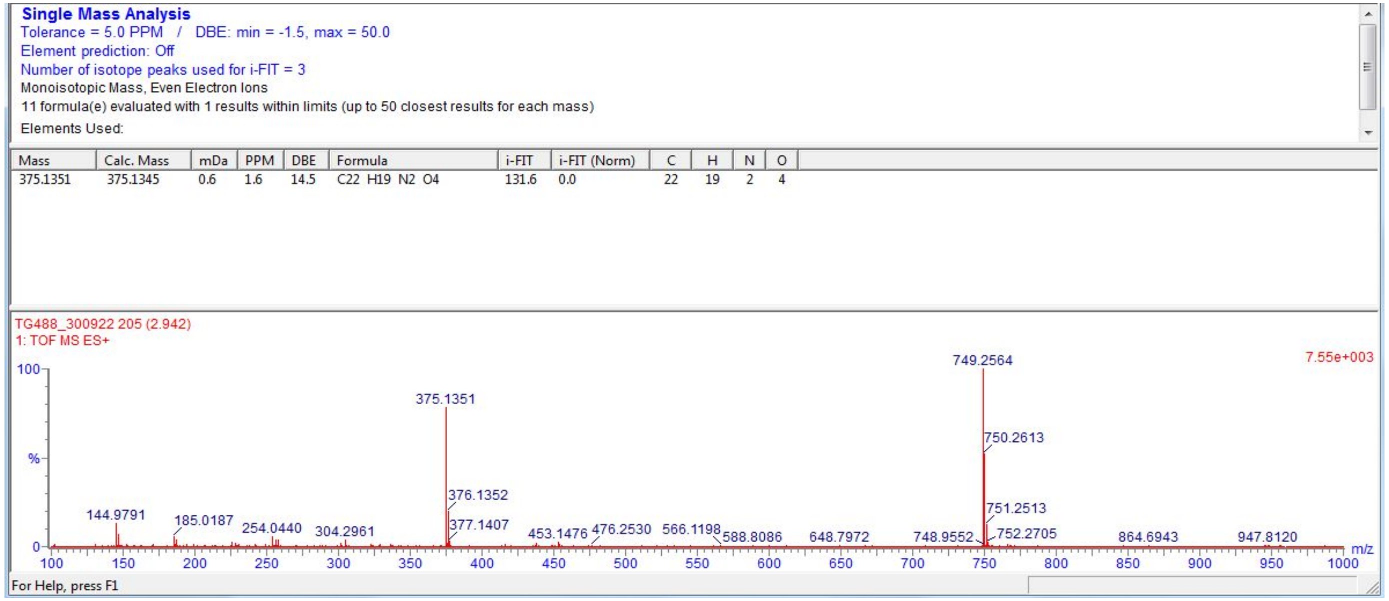
Single Mass Analysis
Tolerance = 5.0 PPM / DBE: min = -1.5, max = 50.0
Element prediction: Off
Number of isotope peaks used for i-FIT = 3
Monoisotopic Mass, Even Electron Ions
11 formula(e) evaluated with 1 results within limits (up to 50 closest results for each mass)
Elements Used:

Mass	Calc. Mass	mDa	PPM	DBE	Formula	i-FIT	i-FIT (Norm)	C	H	N	O
375.1351	375.1345	0.6	1.6	14.5	C22 H19 N2 O4	131.6	0.0	22	19	2	4

Figure S13. ^{1}H-NMR Spectra of 18

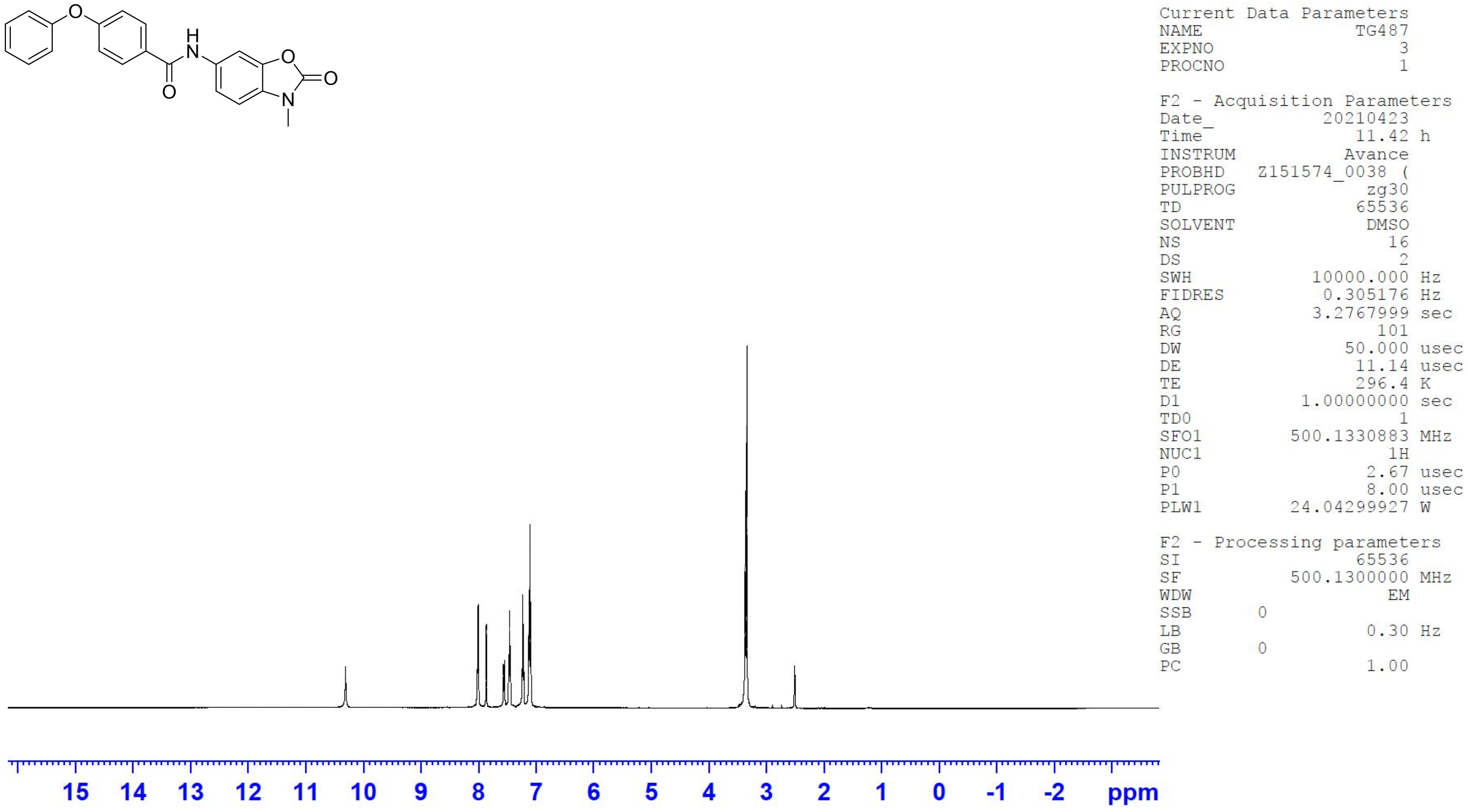

Figure S14. ^{13}C-NMR Spectra of 18

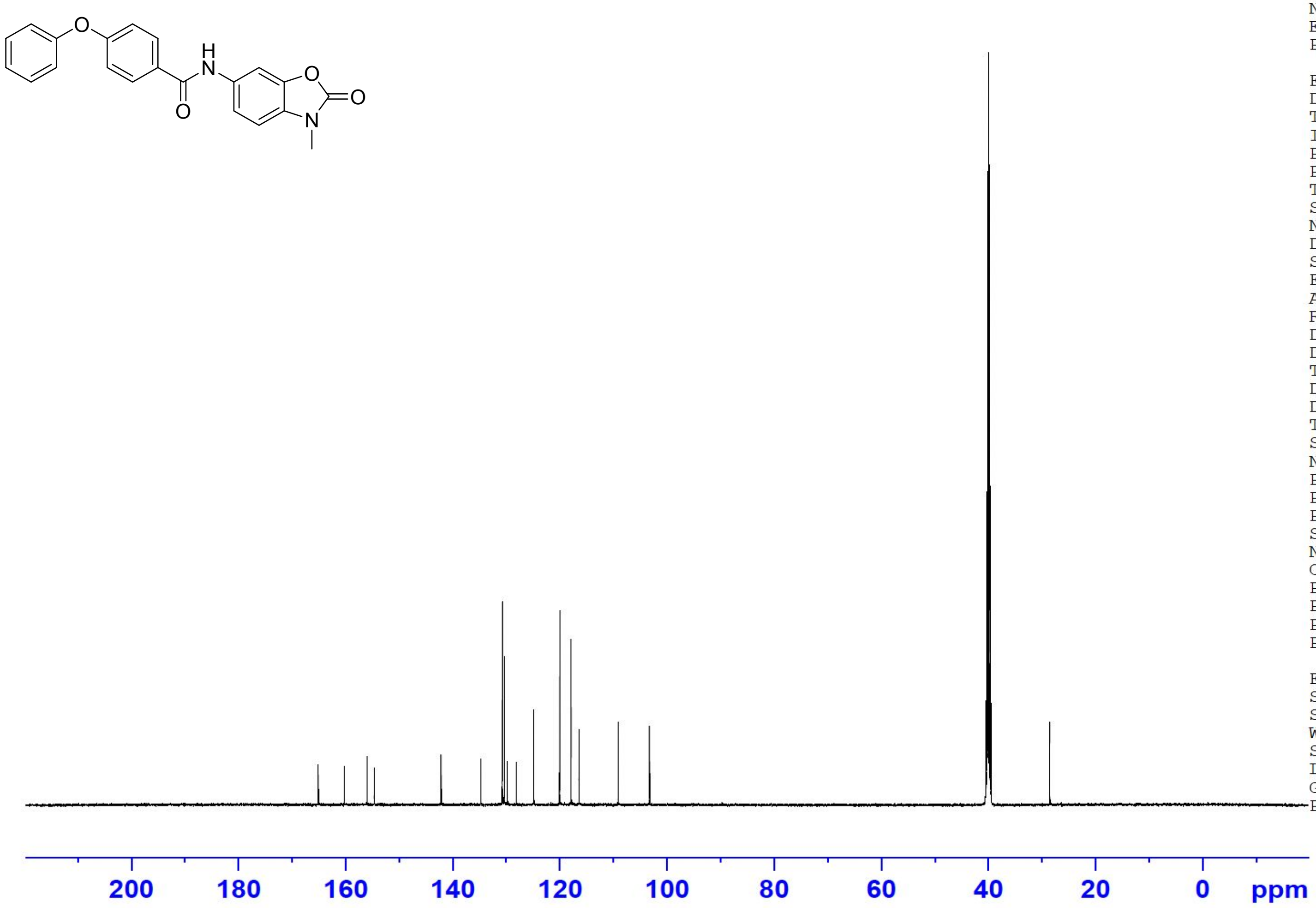

Current Data Parameters	
NAME	TG487
EXPNO	2
PROCNO	1

F2 - Acquisition Parameters	
Date_	20210423
Time	11.30 h
INSTRUM	Avance
PROBHD	Z151574_0038 (
PULPROG	zgpg30
TD	65536
SOLVENT	DMSO
NS	1200
DS	4
SWH	30120.482 Hz
FIDRES	0.919204 Hz
AQ	1.0878977 sec
RG	101
DW	16.600 usec
DE	6.50 usec
TE	297.3 K
D1	2.00000000 sec
D11	0.03000000 sec
TD0	1
SFO1	125.7703643 MHz
NUC1	13C
P0	3.33 usec
P1	10.00 usec
PLW1	85.18099976 W
SFO2	500.1320005 MHz
NUC2	1H
CPDPRG[2	waltz65
PCPD2	80.00 usec
PLW2	24.04299927 W
PLW12	0.24043000 W
PLW13	0.12093000 W

F2 - Processing parameters	
SI	32768
SF	125.7577885 MHz
WDW	EM
SSB	0
LB	1.00 Hz
GB	0
PC	1.40

Figure S15. HRMS Spectra of 18

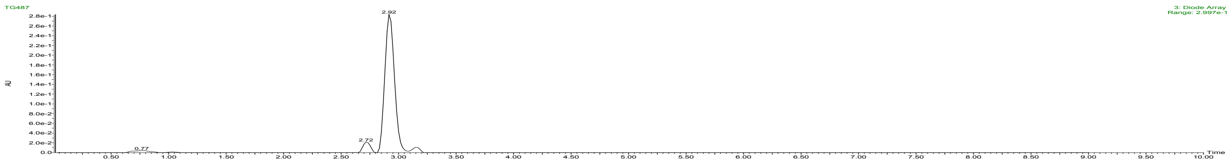

Single Mass Analysis
Tolerance = 5.0 PPM / DBE: min = -1.5, max = 50.0
Element prediction: Off
Number of isotope peaks used for i-FIT = 3
Monoisotopic Mass, Even Electron Ions
11 formula(e) evaluated with 1 results within limits (up to 50 closest results for each mass)
Elements Used:

Mass	Calc. Mass	mDa	PPM	DBE	Formula	i-FIT	i-FIT (Norm)	C	H	N	O
361.1194	361.1188	0.6	1.7	14.5	C21 H17 N2 O4	320.6	0.0	21	17	2	4

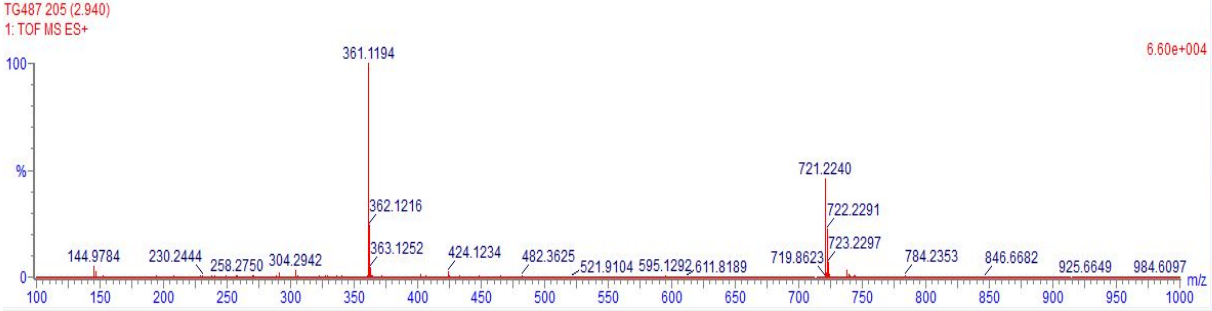

Figure S16. ^{1}H-NMR Spectra of 20

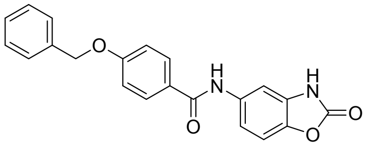

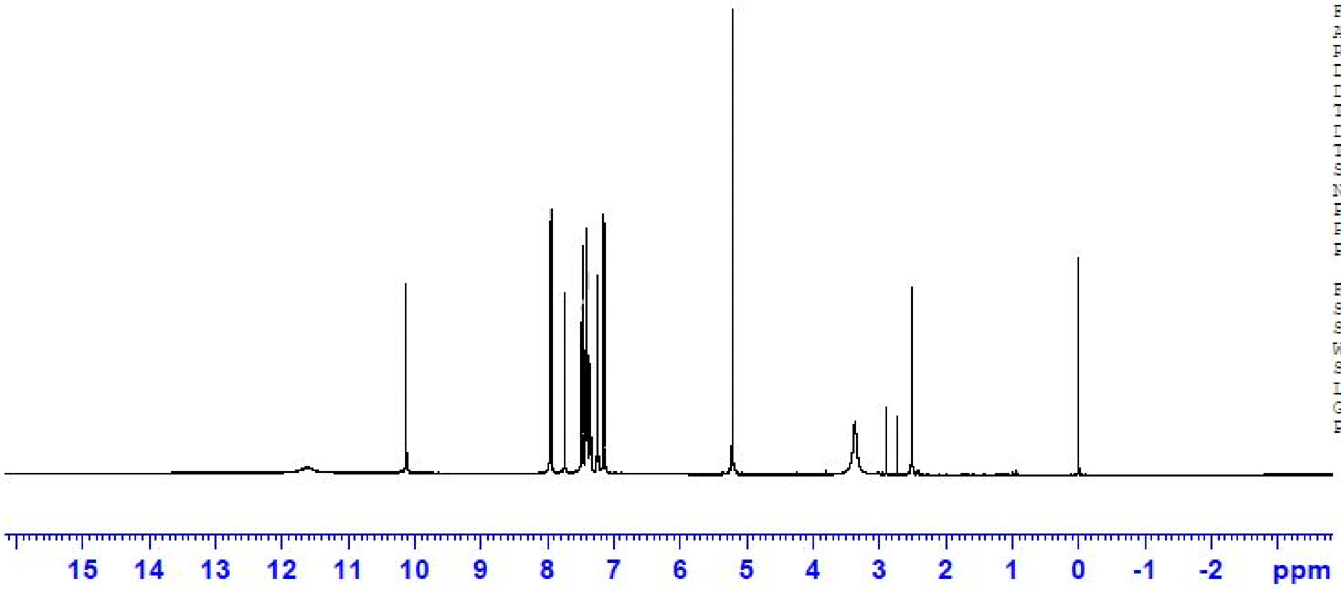

Figure S17. ^{13}C-NMR Spectra of 20

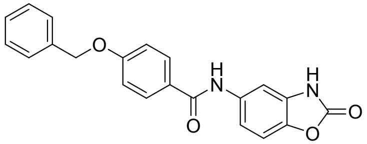

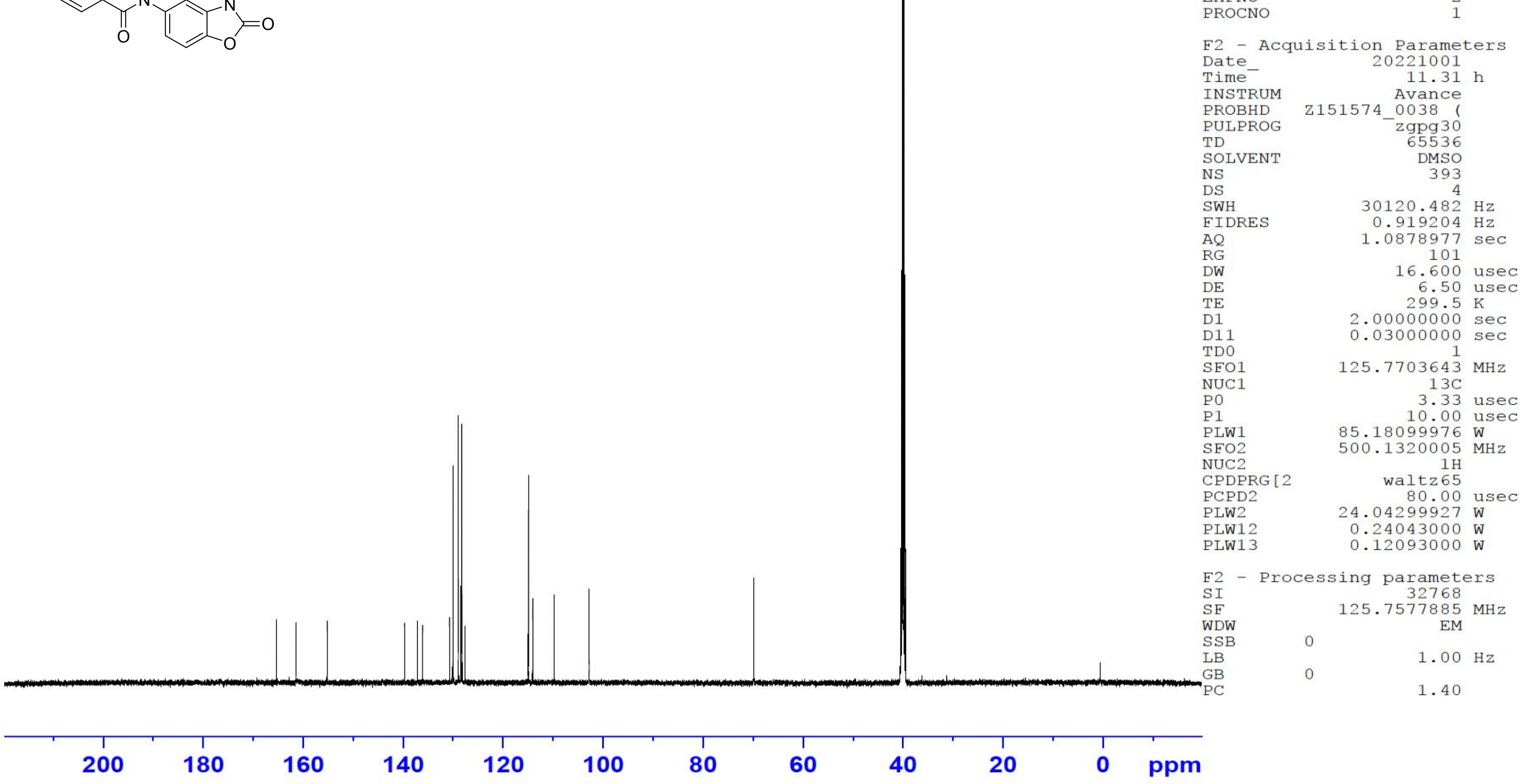

Figure S18. HRMS Spectra of 20

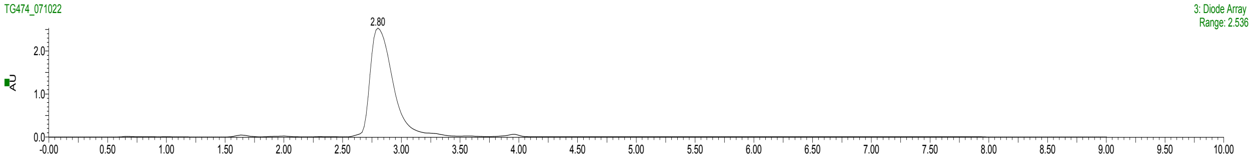

Single Mass Analysis
Tolerance = 5.0 PPM / DBE: min = -1.5, max = 50.0
Element prediction: Off
Number of isotope peaks used for i-FIT = 3
Monoisotopic Mass, Even Electron Ions
20 formula(e) evaluated with 1 results within limits (up to 50 closest results for each mass)
Elements Used:

Mass	Calc. Mass	mDa	PPM	DBE	Formula	i-FIT	i-FIT (Norm)	C	H	N	O
361.1190	361.1188	0.2	0.6	14.5	C21 H17 N2 O4	259.3	0.0	21	17	2	4

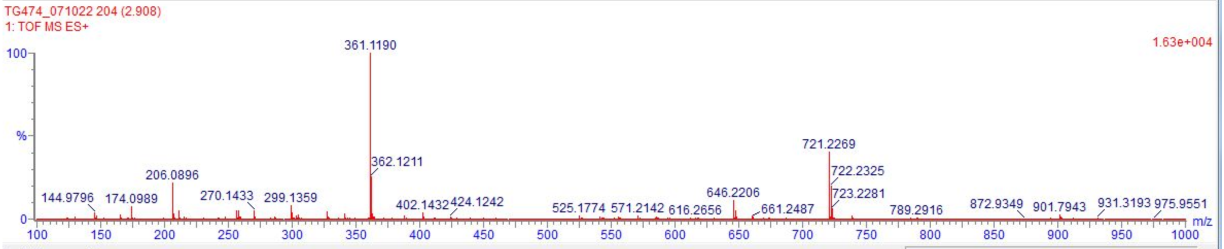

For Help, press F1

Figure S19. ^{1}H-NMR Spectra of 21

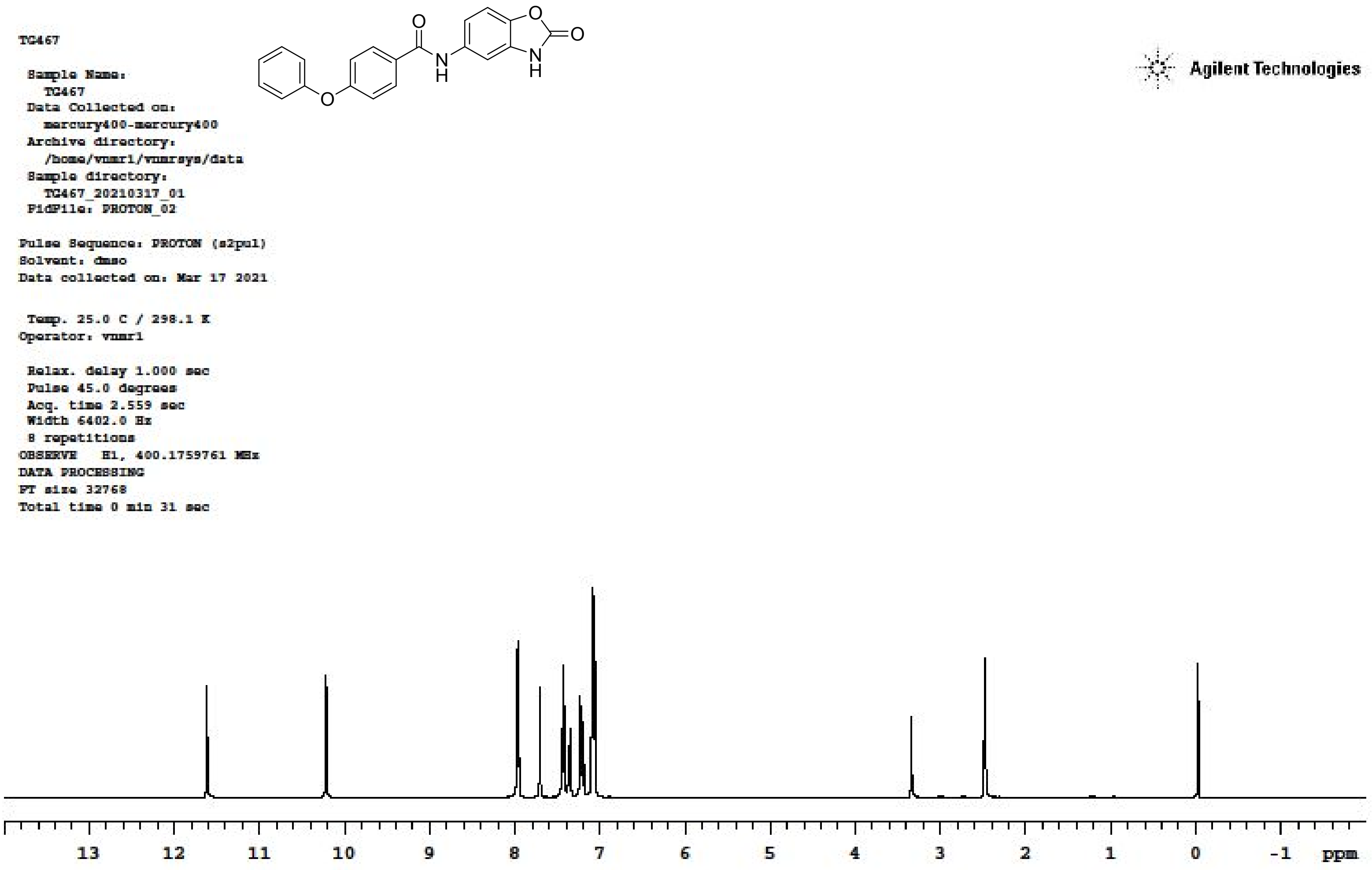

Figure S20. ^{13}C-NMR Spectra of 21

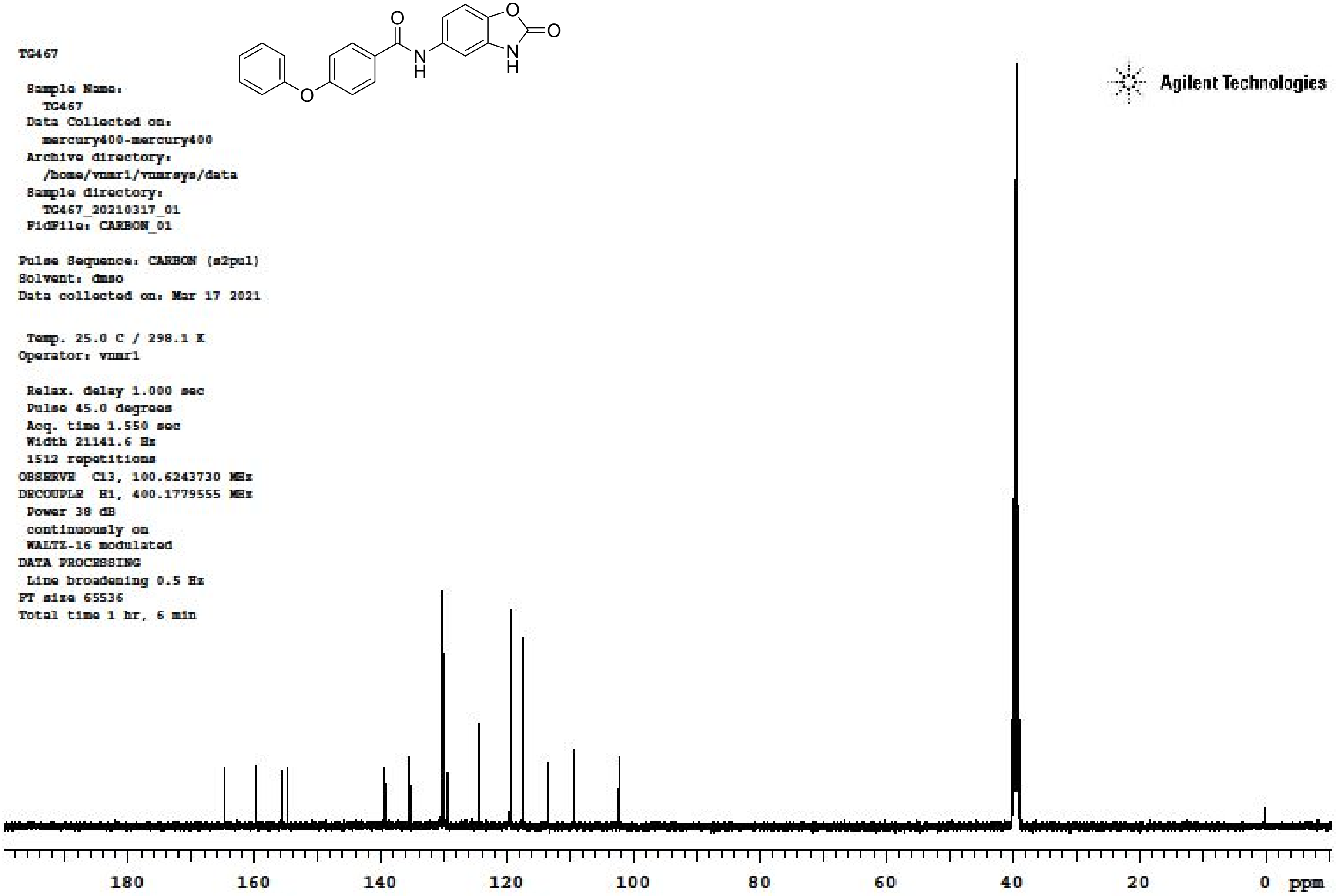

Figure S21. HRMS Spectra of 21

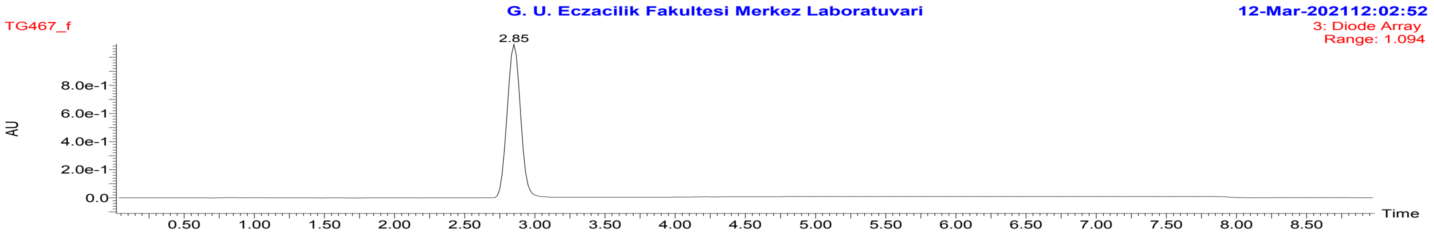

Single Mass Analysis

Tolerance = 5.0 PPM / DBE: min = -1.5, max = 50.0

Element prediction: Off

Number of isotope peaks used for i-FIT = 3

Monoisotopic Mass, Even Electron Ions

17 formula(e) evaluated with 1 results within limits (up to 50 closest results for each mass)

Elements Used:

Mass	Calc. Mass	mDa	PPM	DBE	Formula	i-FIT	i-FIT (Norm)	C	H	N	O
347.1036	347.1032	0.4	1.2	14.5	C20 H15 N2 O4	208.2	0.0	20	15	2	4

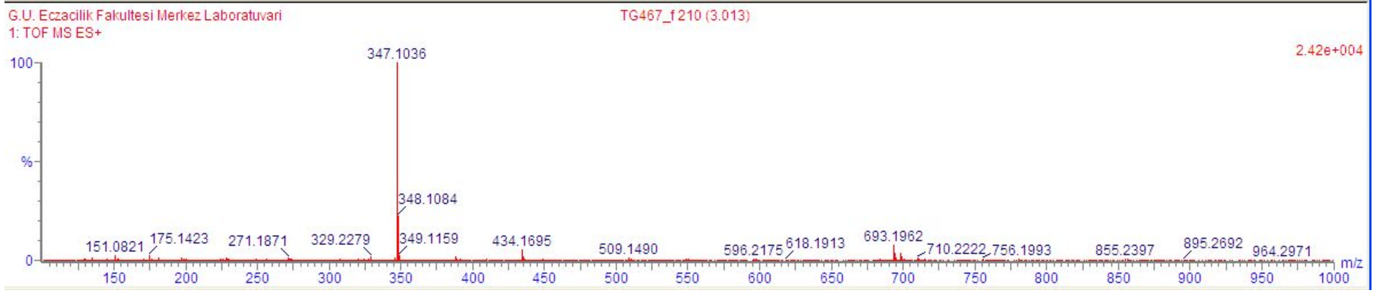

Figure S22. ^{1}H-NMR Spectra of 31

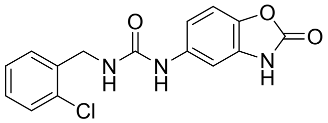

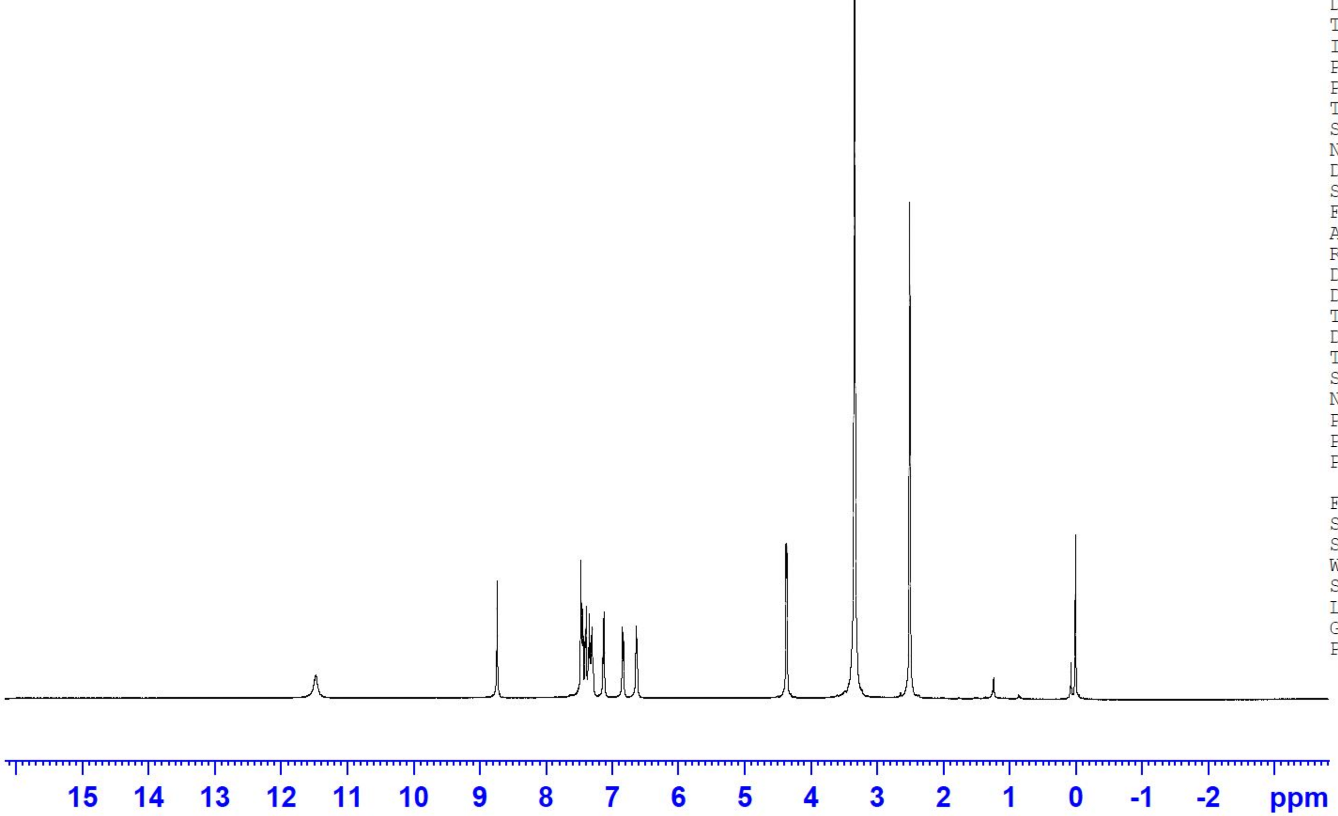


```
Current Data Parameters
NAME             TG598-02
EXPNO                   2
PROCNO                  1

F2 - Acquisition Parameters
Date_            20221001
Time                13.54 h
INSTRUM            Avance
PROBHD   Z151574_0038 (
PULPROG              zg30
TD                  65536
SOLVENT              DMSO
NS                     16
DS                      2
SWH             10000.000 Hz
FIDRES           0.305176 Hz
AQ              3.2767999 sec
RG                    101
DW                 50.000 usec
DE                  11.14 usec
TE                  298.8 K
D1             1.00000000 sec
TD0                     1
SFO1          500.1330883 MHz
NUC1                   1H
P0                   2.67 usec
P1                   8.00 usec
PLW1          24.04299927 W

F2 - Processing parameters
SI                  65536
SF            500.1300000 MHz
WDW                    EM
SSB      0
LB                   0.30 Hz
GB       0
PC                   1.00
```

Figure S23. ^{13}C-NMR Spectra of 31

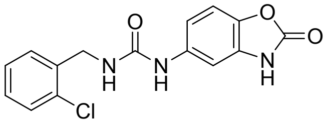

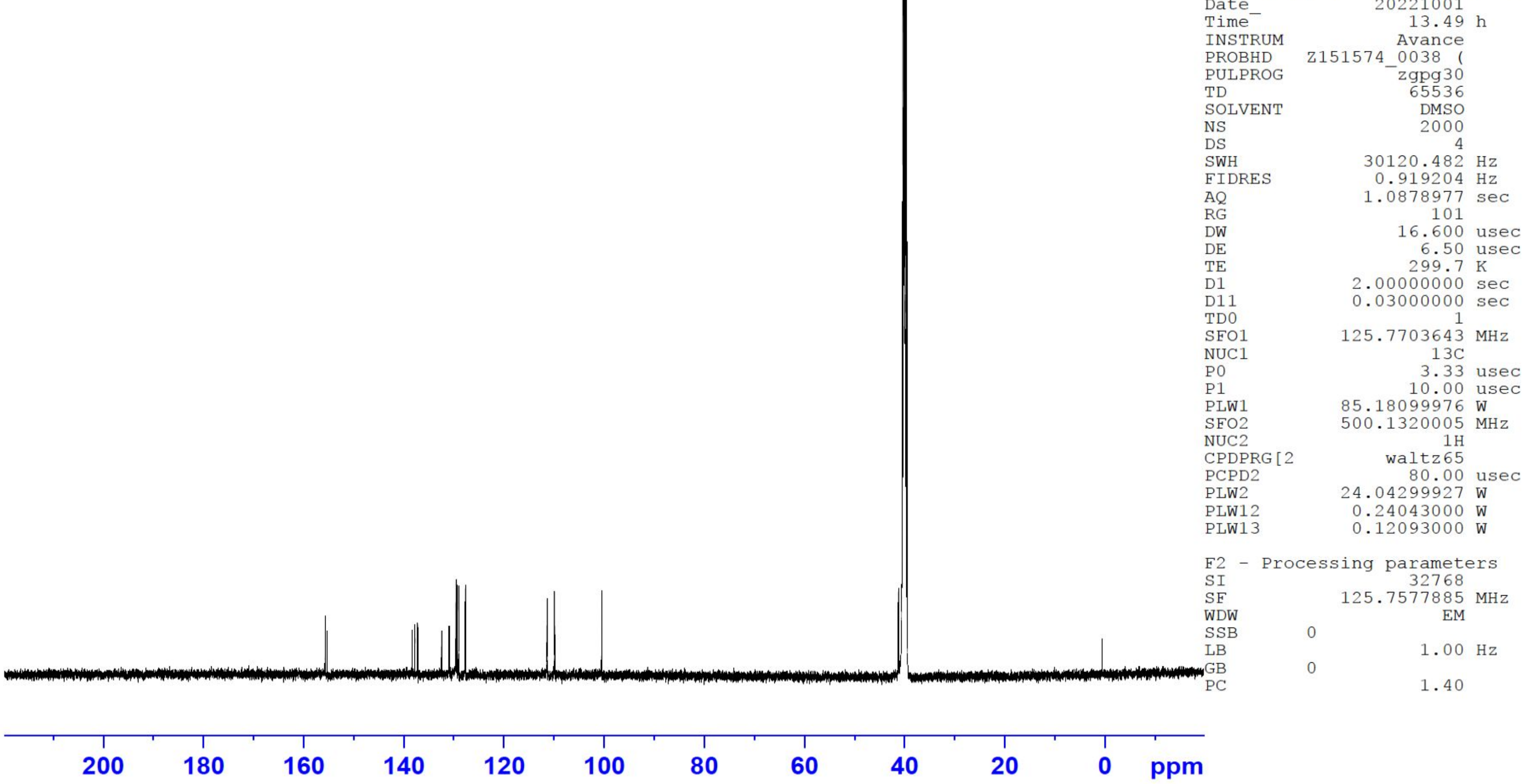

Figure S24. HRMS Spectra of 31

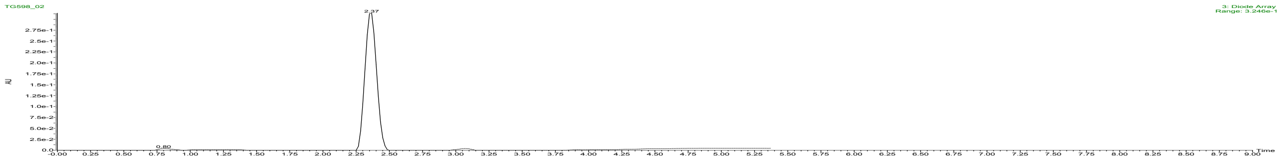

Single Mass Analysis
Tolerance = 5.0 PPM / DBE: min = -1.5, max = 50.0
Element prediction: Off
Number of isotope peaks used for i-FIT = 3
Monoisotopic Mass, Even Electron Ions
33 formula(e) evaluated with 1 results within limits (up to 50 closest results for each mass)
Elements Used:

Mass	Calc. Mass	mDa	PPM	DBE	Formula	i-FIT	i-FIT (Norm)	C	H	N	O	Cl
318.0648	318.0645	0.3	0.9	10.5	C15 H13 N3 O3 Cl	317.4	0.0	15	13	3	3	1

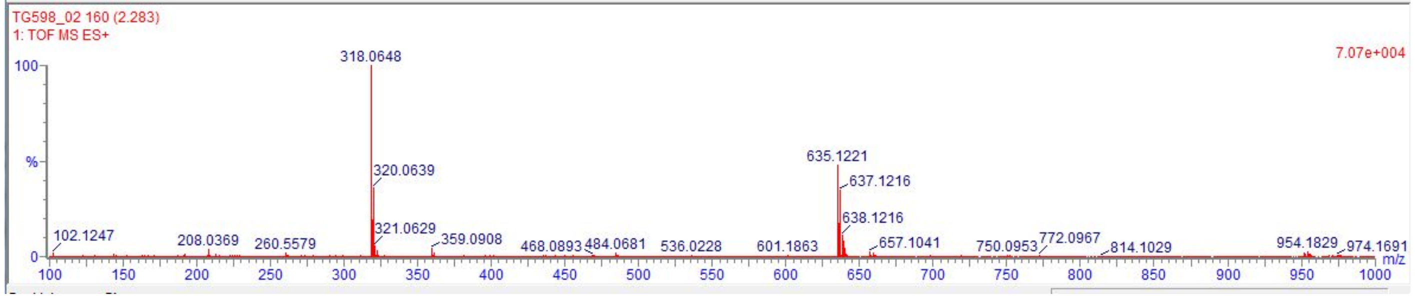

Figure S25. ^{1}H-NMR Spectra of 32

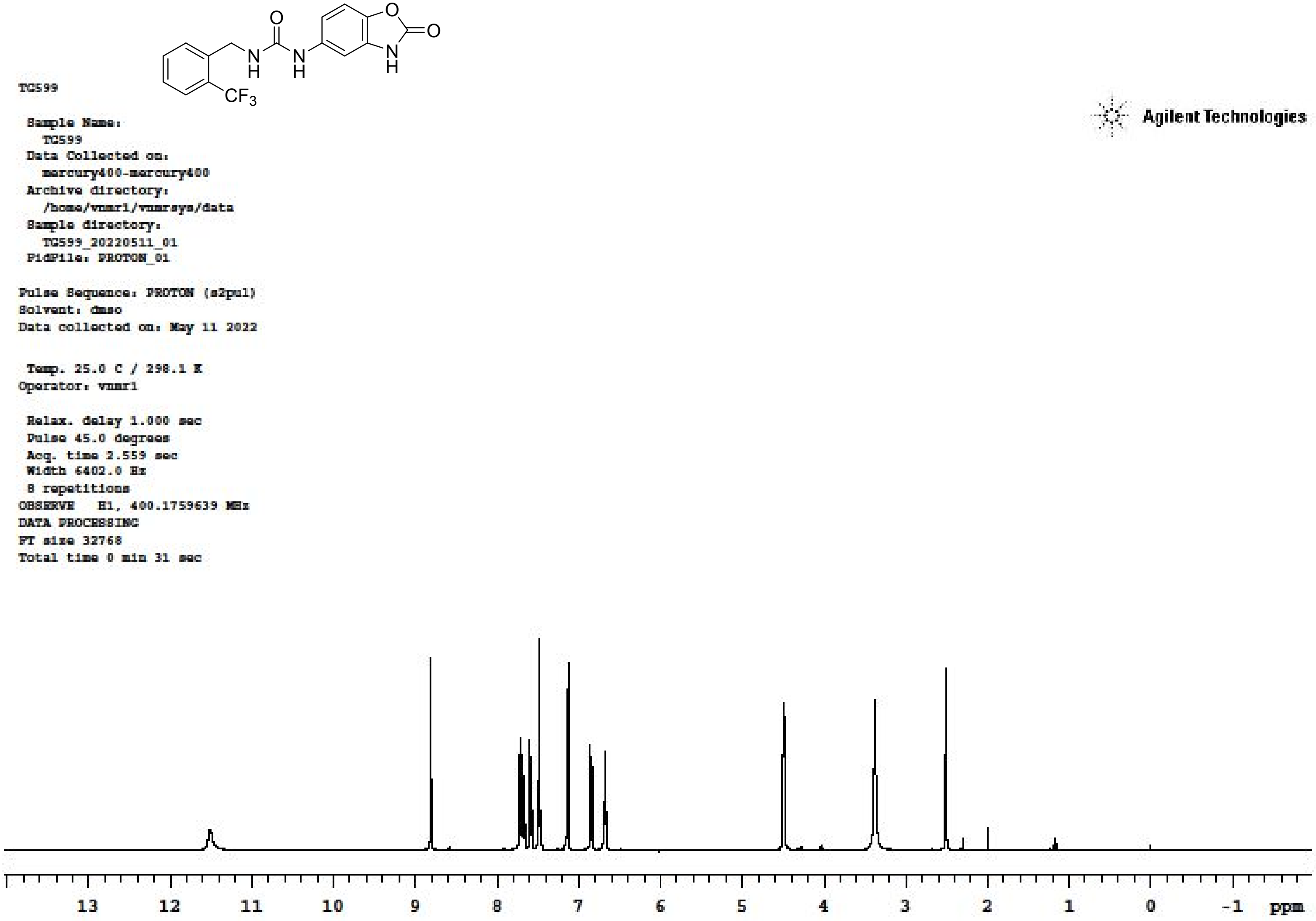

Figure S26. ^{13}C-NMR Spectra of 32

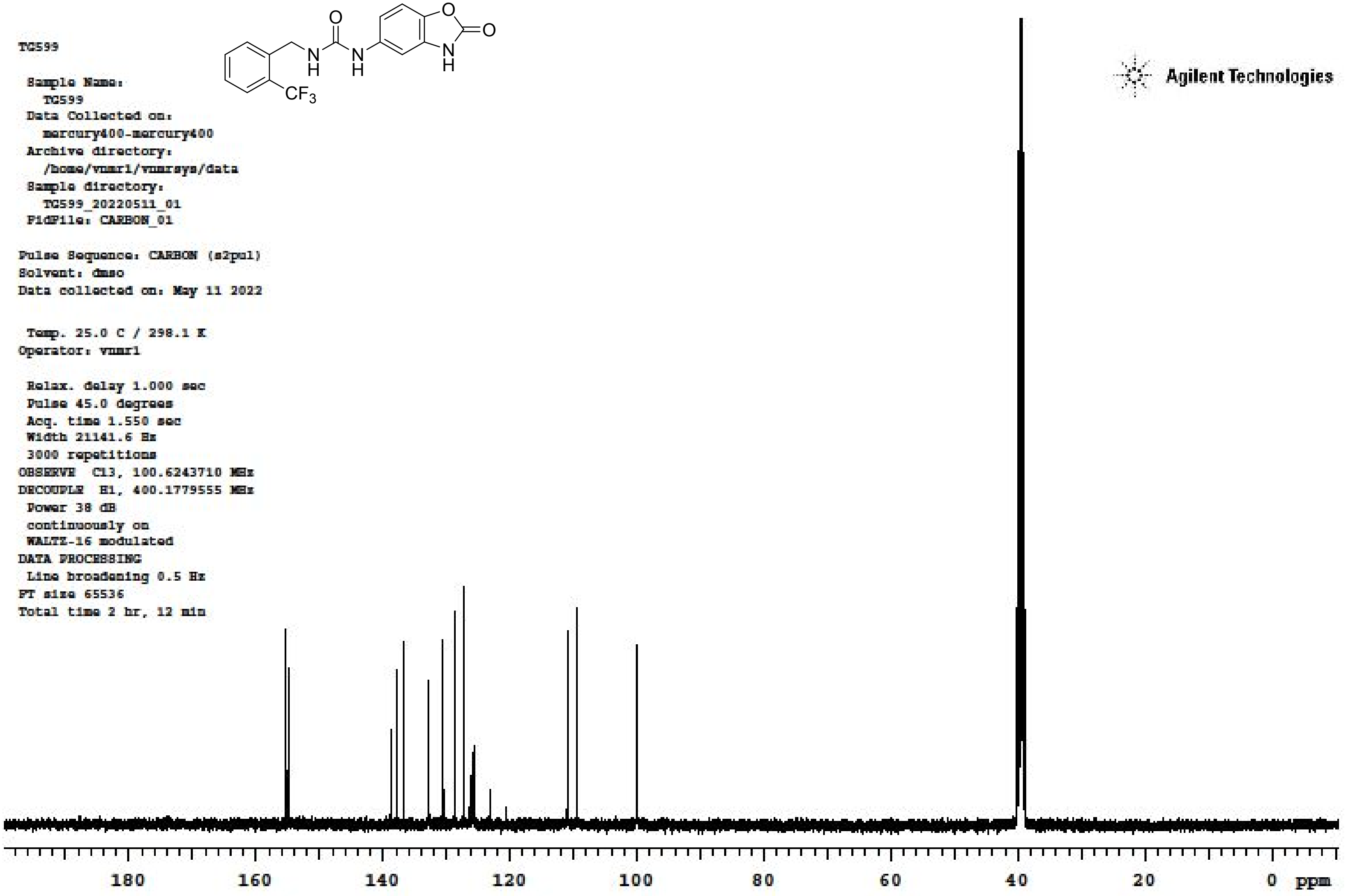

Figure S27. HRMS Spectra of 32

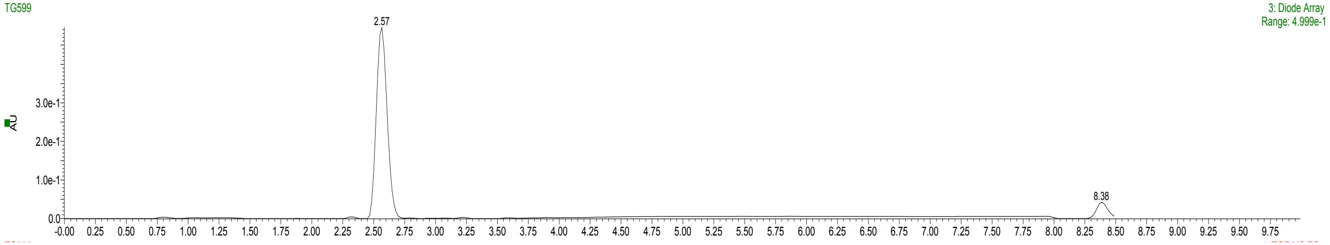

Single Mass Analysis
Tolerance = 5.0 PPM / DBE: min = -1.5, max = 50.0
Element prediction: Off
Number of isotope peaks used for i-FIT = 3
Monoisotopic Mass, Even Electron Ions
35 formula(e) evaluated with 1 results within limits (up to 50 closest results for each mass)
Elements Used:

Mass	Calc. Mass	mDa	PPM	DBE	Formula	i-FIT	i-FIT (Norm)	C	H	N	O	F
352.0924	352.0909	1.5	4.3	10.5	C16 H13 N3 O3 F3	268.4	0.0	16	13	3	3	3

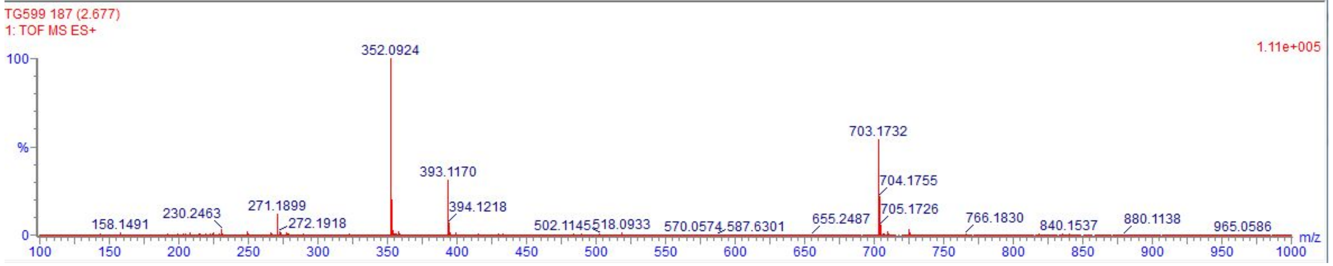

Figure S28. ^{1}H-NMR Spectra of 33

BYZ23

Sample Name:
BYZ23
Data Collected on:
mercury400-mercury400
Archive directory:
/home/vnmr1/vnmrsys/data
Sample directory:
BYZ23_20220510_01
FidFile: PROTON_01

Pulse Sequence: PROTON (s2pul)
Solvent: dmso
Data collected on: May 10 2022

Temp. 25.0 C / 298.1 K
Operator: vnmr1

Relax. delay 1.000 sec
Pulse 45.0 degrees
Acq. time 2.559 sec
Width 6402.0 Hz
8 repetitions
OBSERVE H1, 400.1759761 MHz
DATA PROCESSING
FT size 32768
Total time 0 min 31 sec

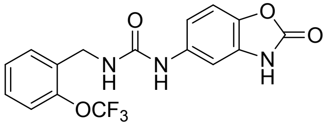

Agilent Technologies

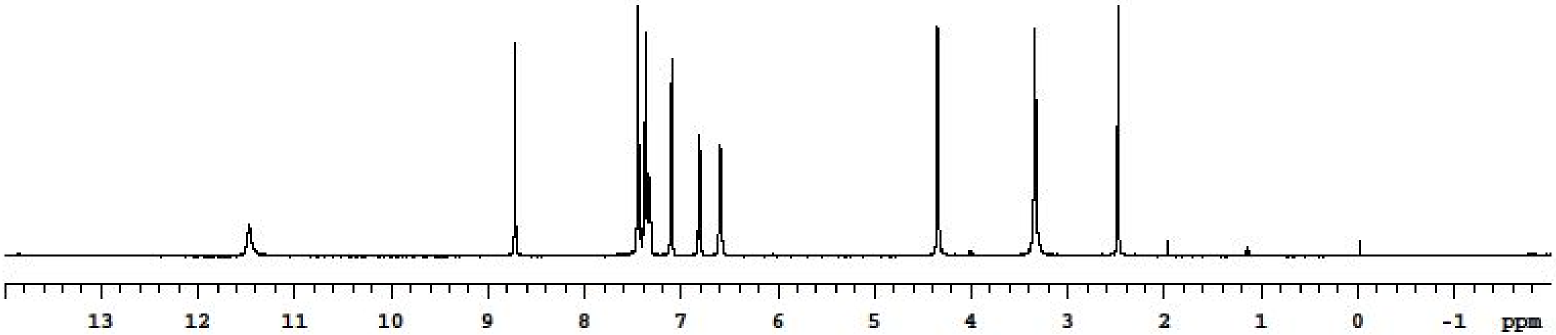

Figure S29. ^{13}C-NMR Spectra of 33

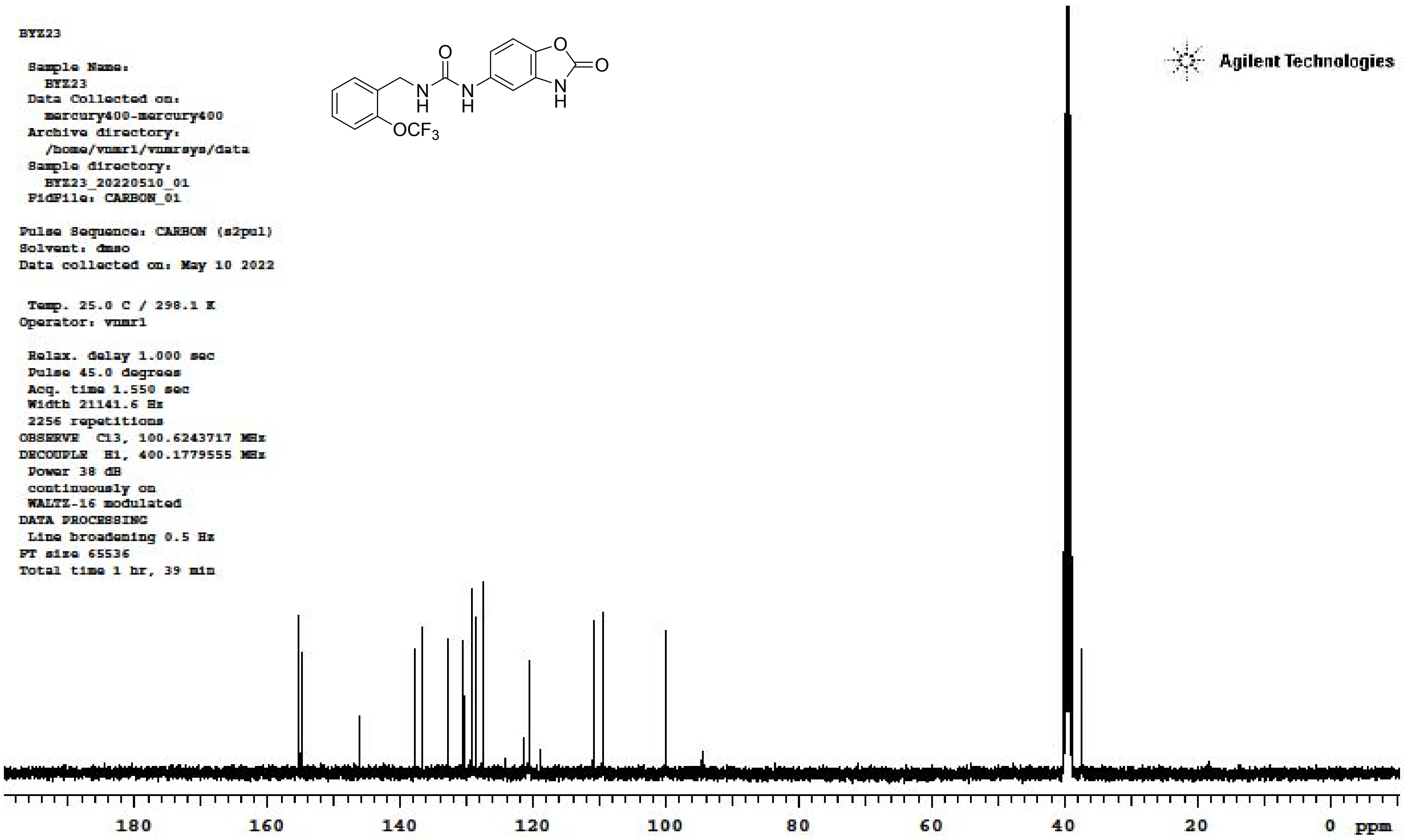

Figure S30. HRMS Spectra of 33

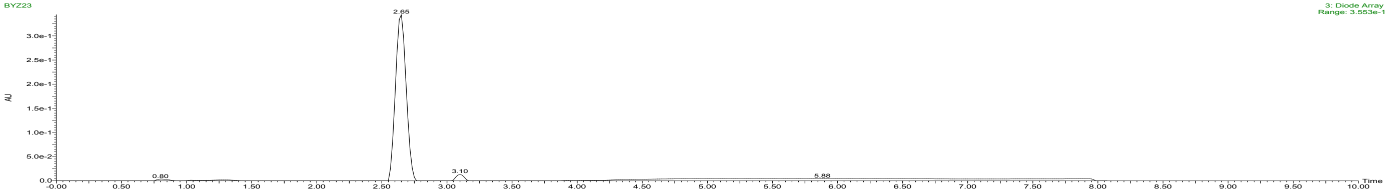

Single Mass Analysis
Tolerance = 5.0 PPM / DBE: min = -1.5, max = 50.0
Element prediction: Off
Number of isotope peaks used for i-FIT = 3
Monoisotopic Mass, Even Electron Ions
35 formula(e) evaluated with 1 results within limits (up to 50 closest results for each mass)
Elements Used:

Mass	Calc. Mass	mDa	PPM	DBE	Formula	i-FIT	i-FIT (Norm)	C	H	N	O	F
368.0869	368.0858	1.1	3.0	10.5	C16 H13 N3 O4 F3	233.2	0.0	16	13	3	4	3

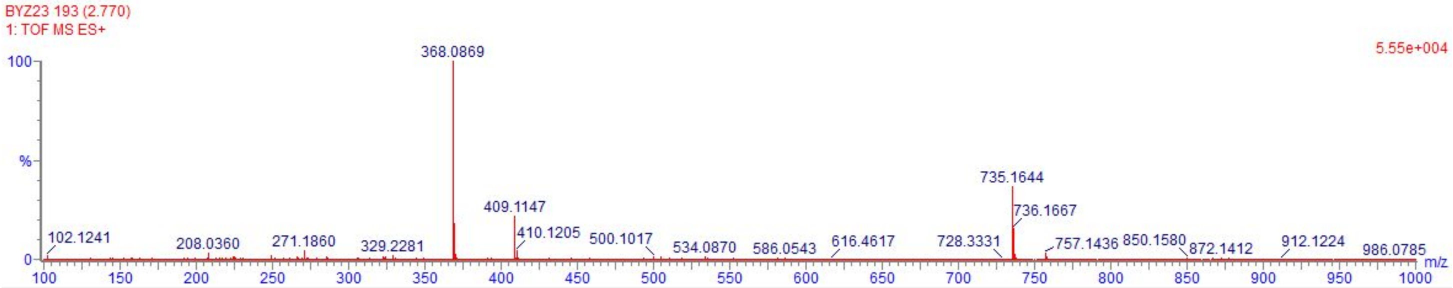

Figure S31. ¹H-NMR Spectra of 34

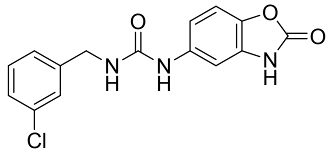

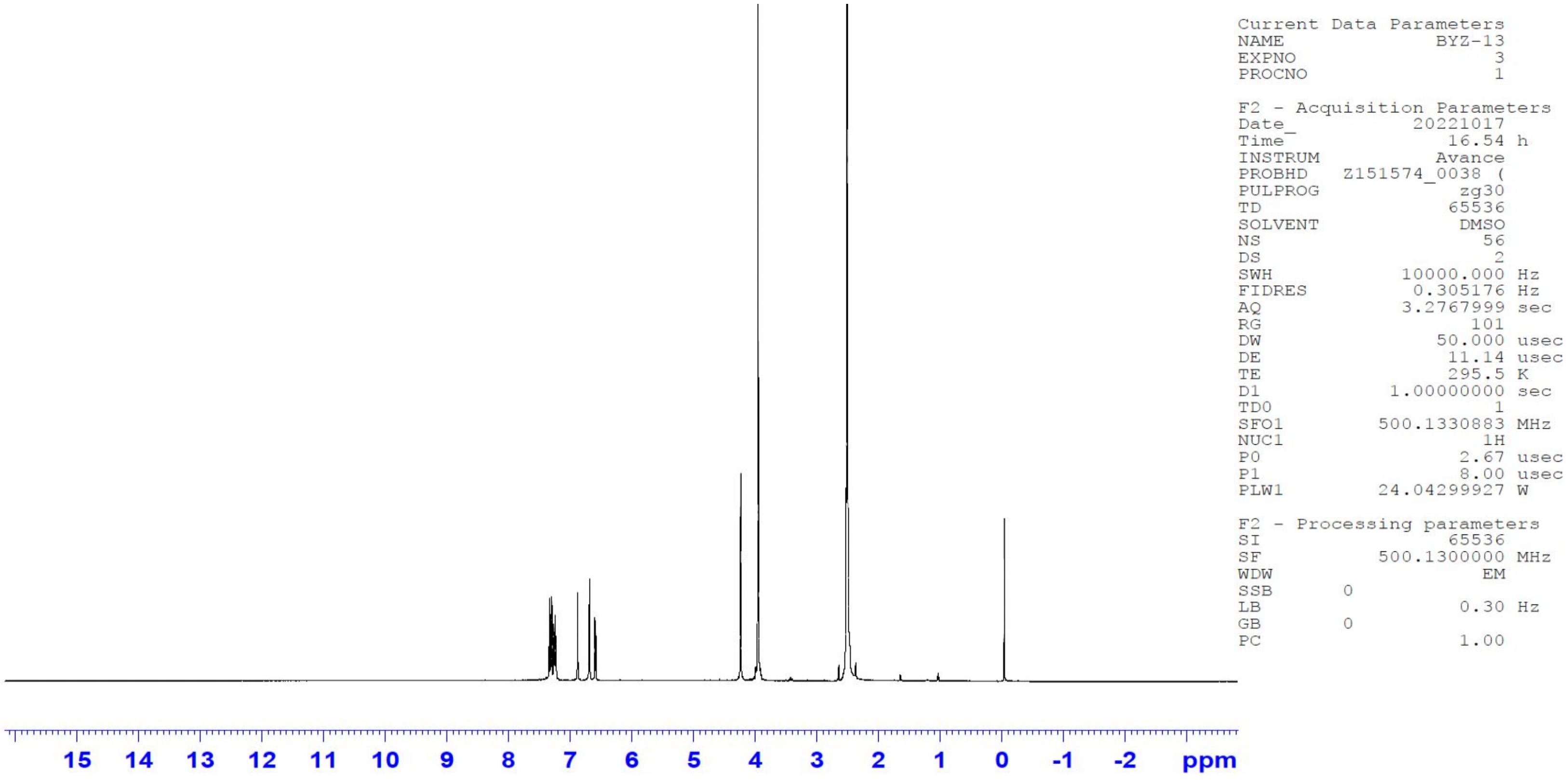

Figure S32. ^{13}C-NMR Spectra of 34

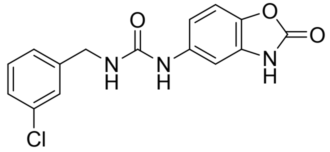

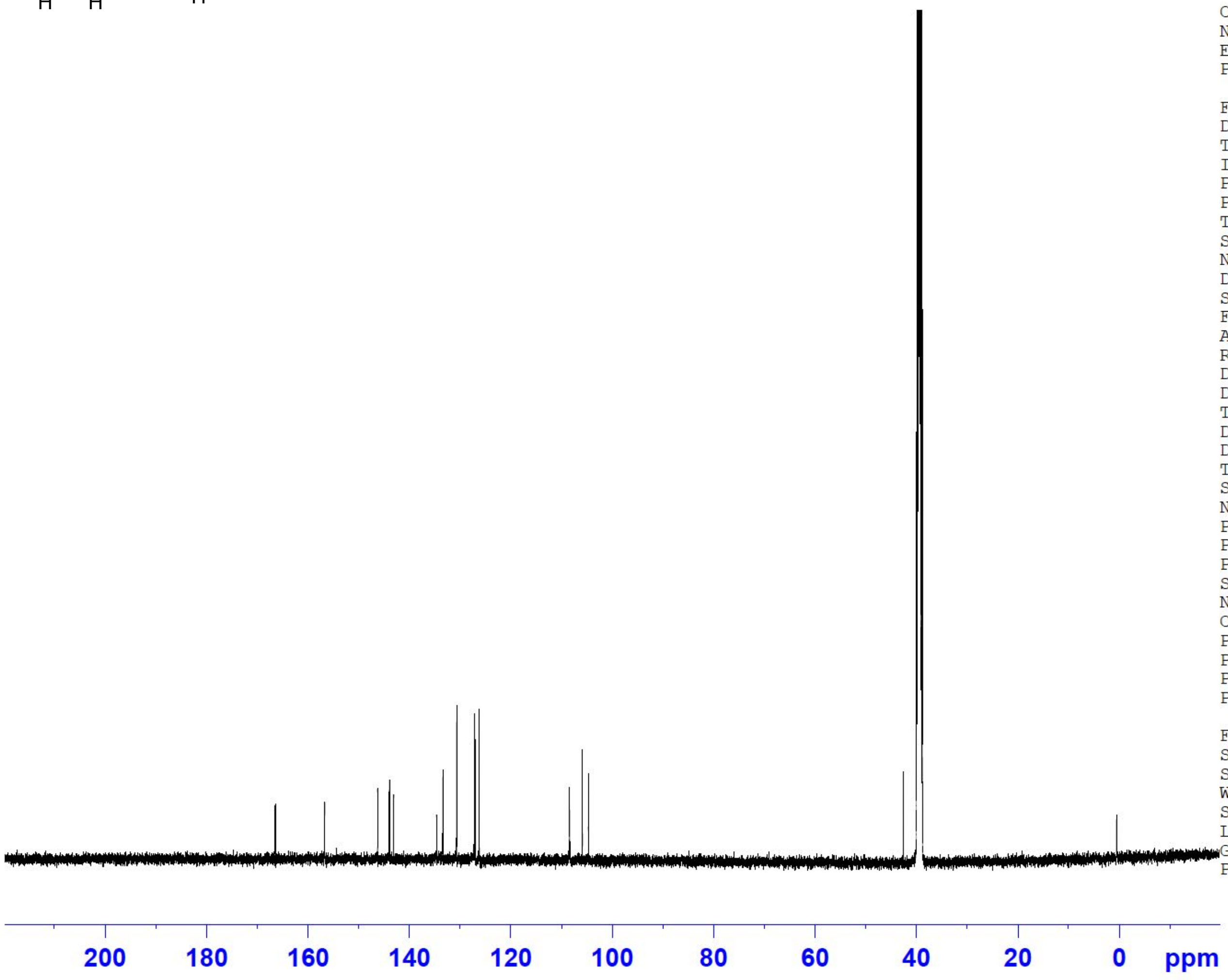


```
Current Data Parameters
NAME                BYZ-13
EXPNO                    2
PROCNO                   1

F2 - Acquisition Parameters
Date_             20221017
Time                 16.45 h
INSTRUM             Avance
PROBHD    Z151574_0038 (
PULPROG            zgpg30
TD                   65536
SOLVENT               DMSO
NS                    2000
DS                       4
SWH              30120.482 Hz
FIDRES            0.919204 Hz
AQ               1.0878977 sec
RG                     101
DW                  16.600 usec
DE                    6.50 usec
TE                   296.5 K
D1              2.00000000 sec
D11             0.03000000 sec
TD0                      1
SFO1           125.7703643 MHz
NUC1                   13C
P0                    3.33 usec
P1                   10.00 usec
PLW1           85.18099976 W
SFO2           500.1320005 MHz
NUC2                    1H
CPDPRG[2          waltz65
PCPD2                80.00 usec
PLW2           24.04299927 W
PLW12           0.24043000 W
PLW13           0.12093000 W

F2 - Processing parameters
SI                   32768
SF             125.7577885 MHz
WDW                     EM
SSB      0
LB                    1.00 Hz
GB       0
PC                    1.40
```

Figure S33. HRMS Spectra of 34

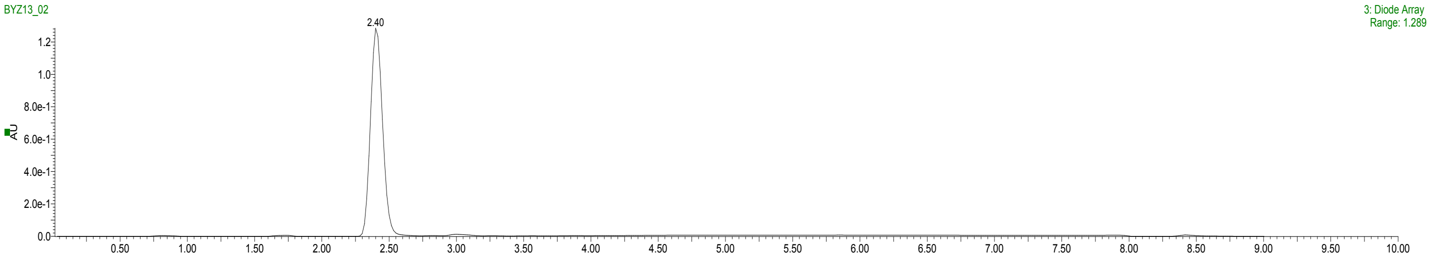

Single Mass Analysis
Tolerance = 5.0 PPM / DBE: min = -1.5, max = 50.0
Element prediction: Off
Number of isotope peaks used for i-FIT = 3
Monoisotopic Mass, Even Electron Ions
8 formula(e) evaluated with 1 results within limits (up to 50 closest results for each mass)
Elements Used:

Mass	Calc. Mass	mDa	PPM	DBE	Formula	i-FIT	i-FIT (Norm)	C	H	N	O	Cl
318.0657	318.0645	1.2	3.8	10.5	C15 H13 N3 O3 Cl	357.9	0.0	15	13	3	3	1

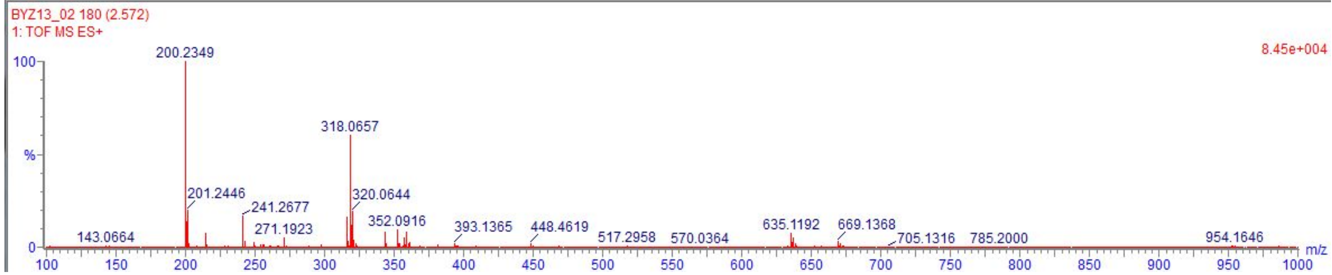

Figure S34. ^{1}H-NMR Spectra of 35

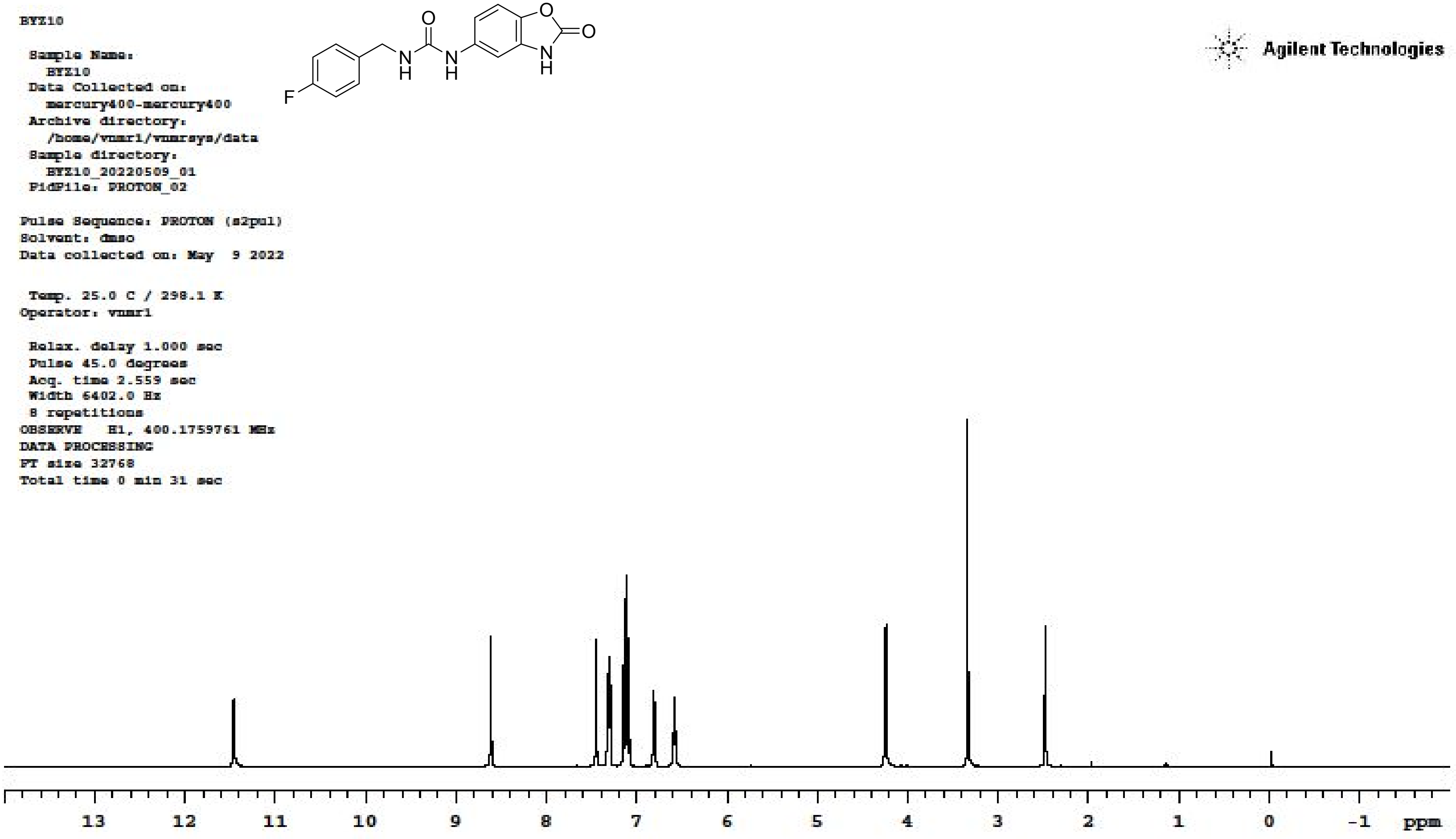

Figure S35. ^{13}C-NMR Spectra of 35

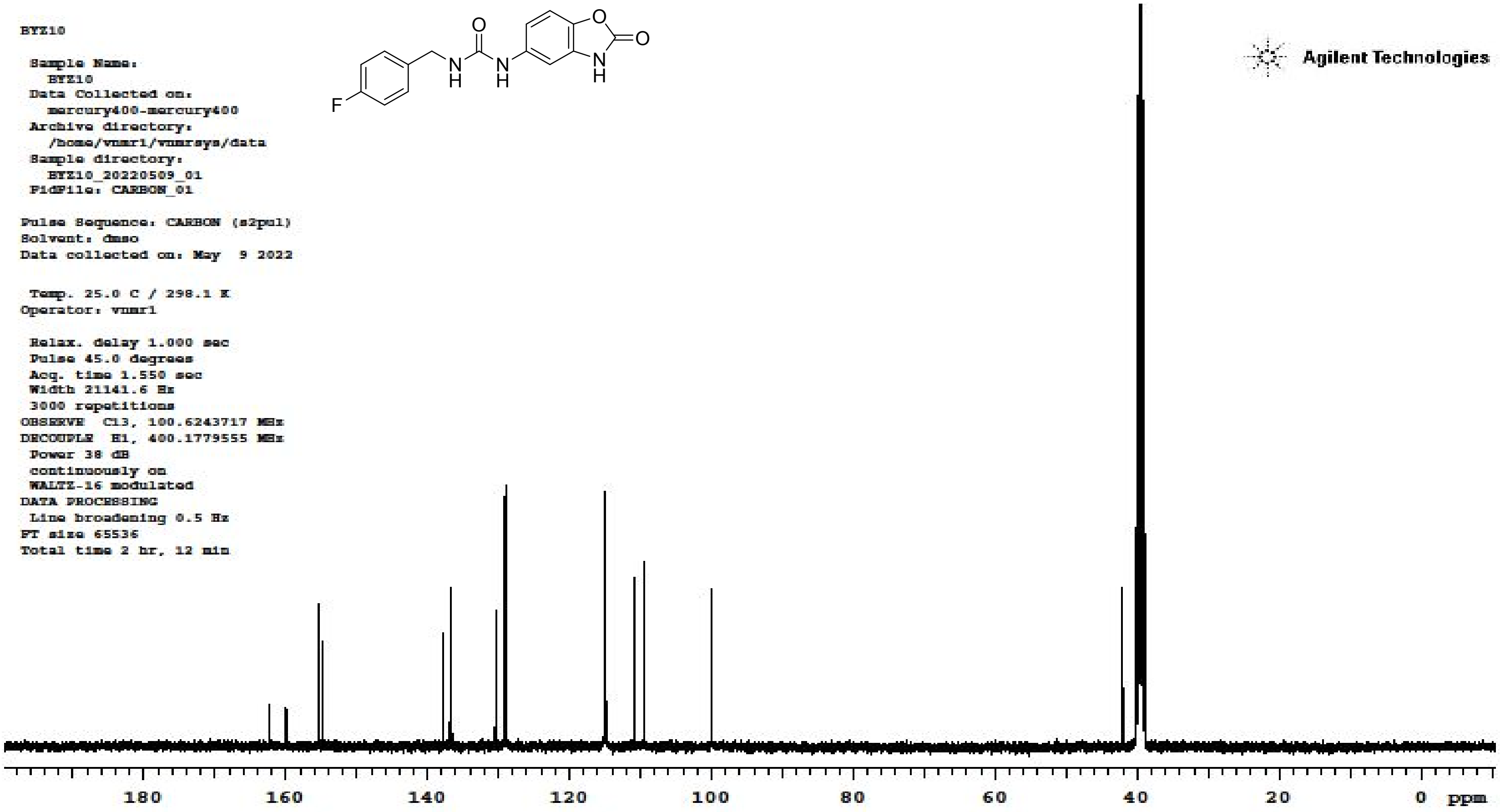

Figure S36. HRMS Spectra of 35

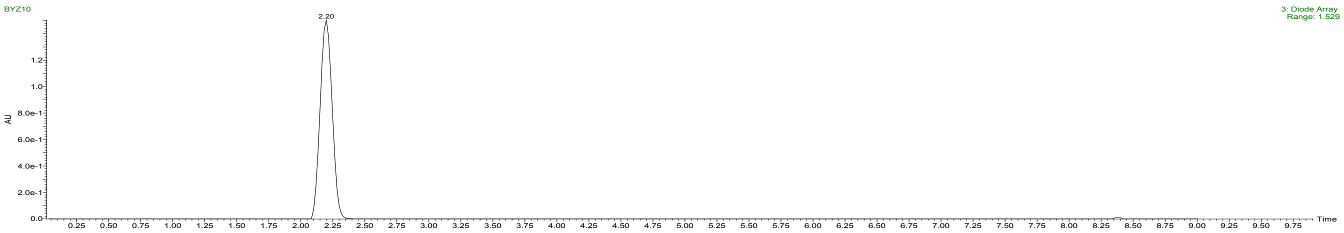

Single Mass Analysis
Tolerance = 5.0 PPM / DBE: min = -1.5, max = 50.0
Element prediction: Off
Number of isotope peaks used for i-FIT = 3
Monoisotopic Mass, Even Electron Ions
19 formula(e) evaluated with 1 results within limits (up to 50 closest results for each mass)
Elements Used:

Mass	Calc. Mass	mDa	PPM	DBE	Formula	i-FIT	i-FIT (Norm)	C	H	N	O	F
302.0928	302.0941	-1.3	-4.3	10.5	C15 H13 N3 O3 F	77.4	0.0	15	13	3	3	1

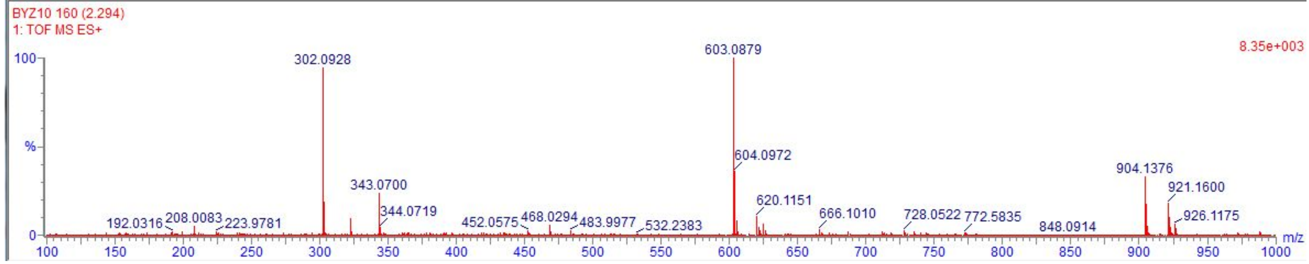

Figure S37. ^{1}H-NMR Spectra of 36

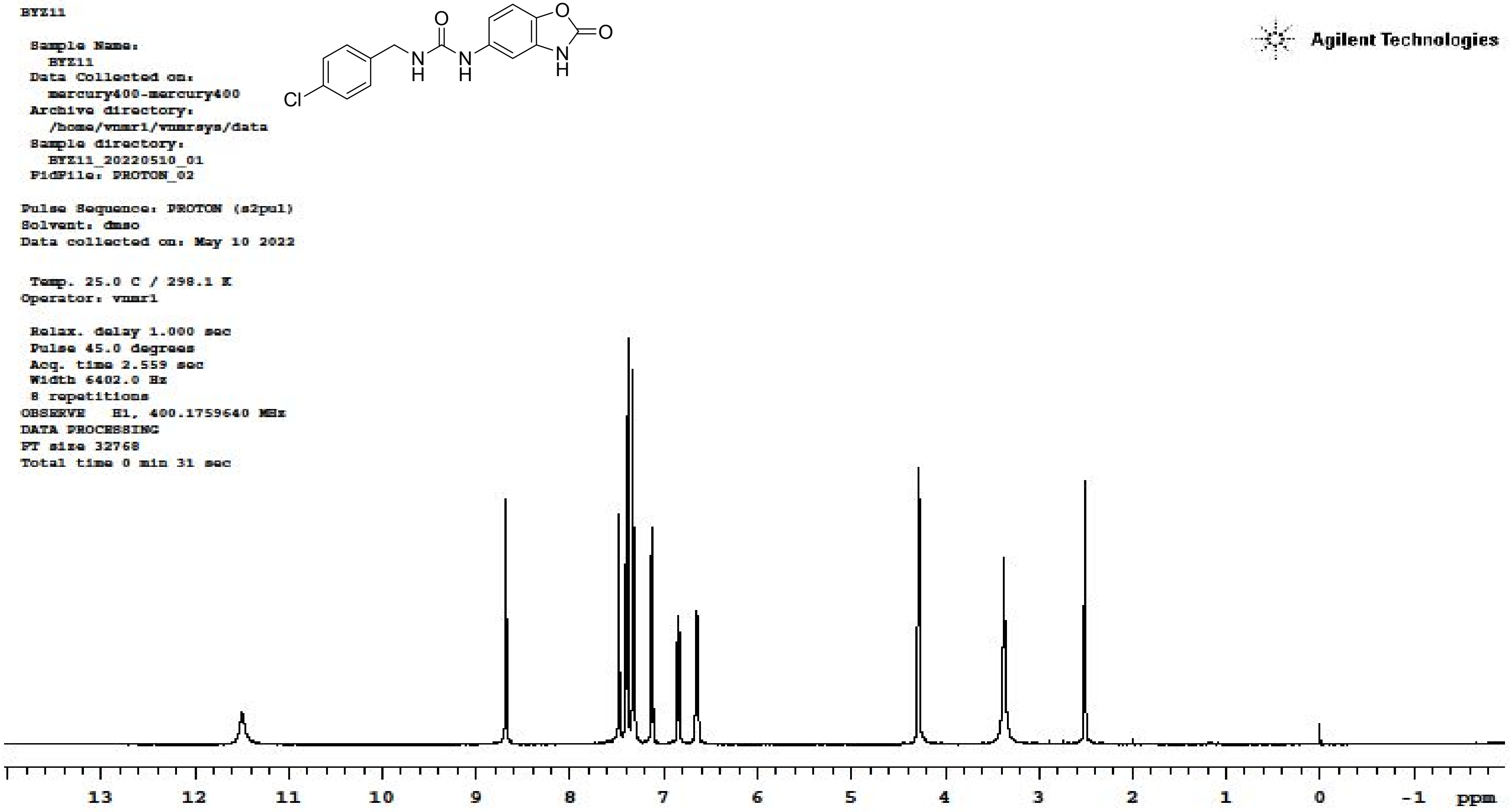

Figure S38. ^{13}C-NMR Spectra of 36

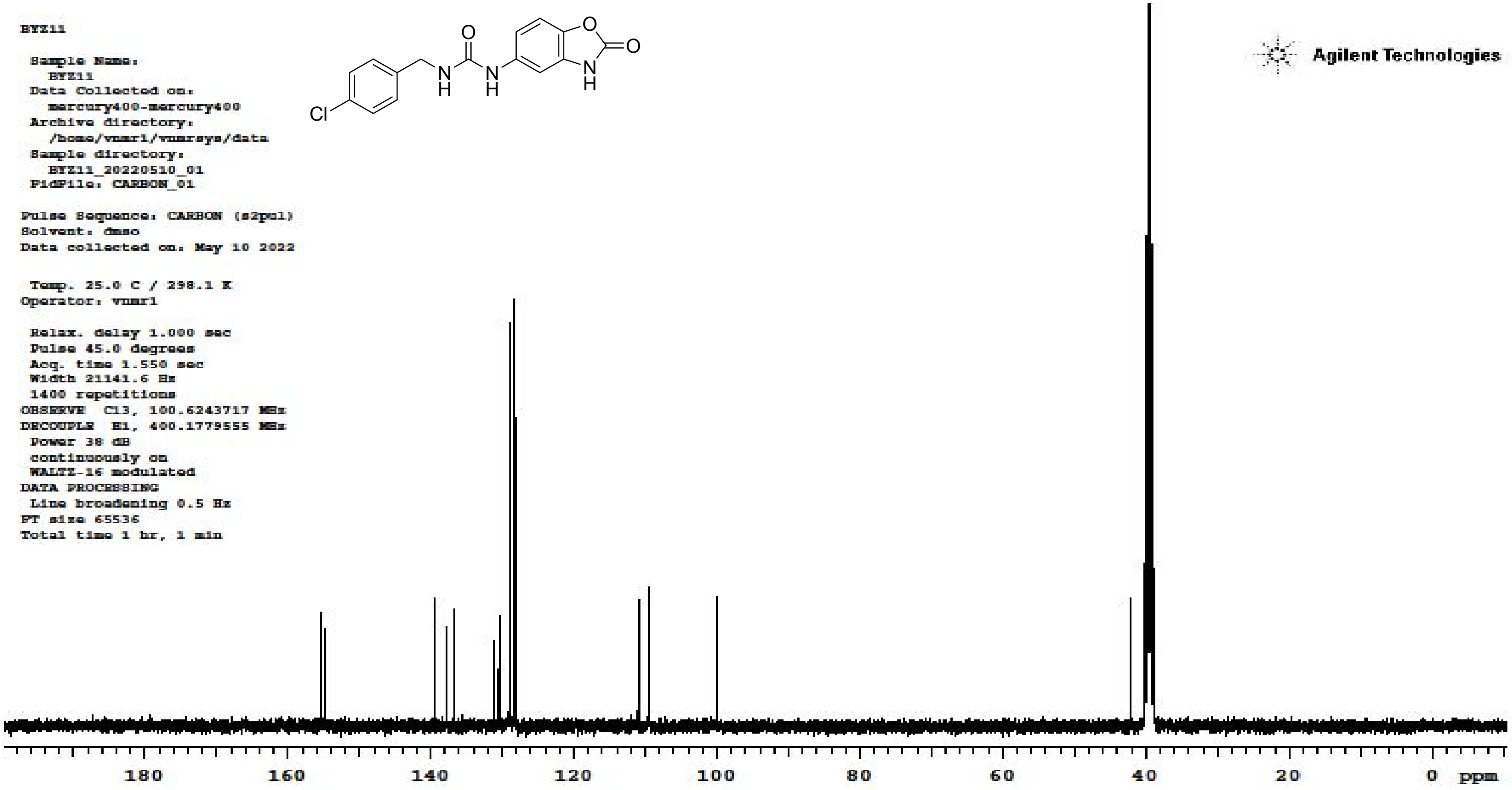

Figure S39. HRMS Spectra of 36

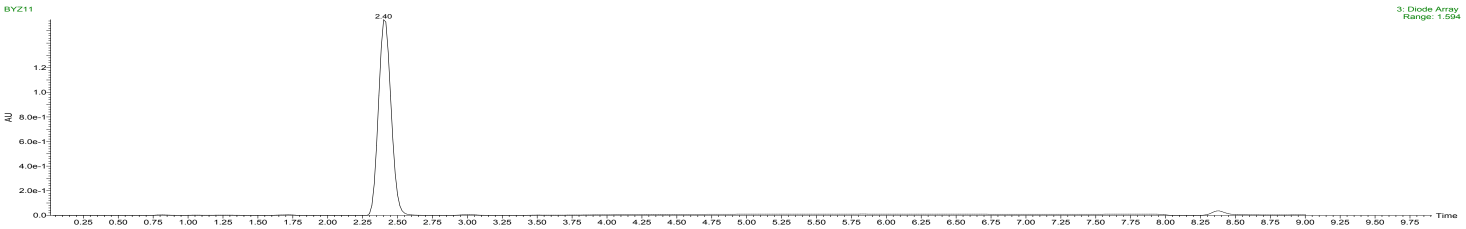

Single Mass Analysis
Tolerance = 5.0 PPM / DBE: min = -1.5, max = 50.0
Element prediction: Off
Number of isotope peaks used for i-FIT = 3
Monoisotopic Mass, Even Electron Ions
21 formula(e) evaluated with 1 results within limits (up to 50 closest results for each mass)
Elements Used:

Mass	Calc. Mass	mDa	PPM	DBE	Formula	i-FIT	i-FIT (Norm)	C	H	N	O	Cl
318.0641	318.0645	-0.4	-1.3	10.5	C15 H13 N3 O3 Cl	60.0	0.0	15	13	3	3	1

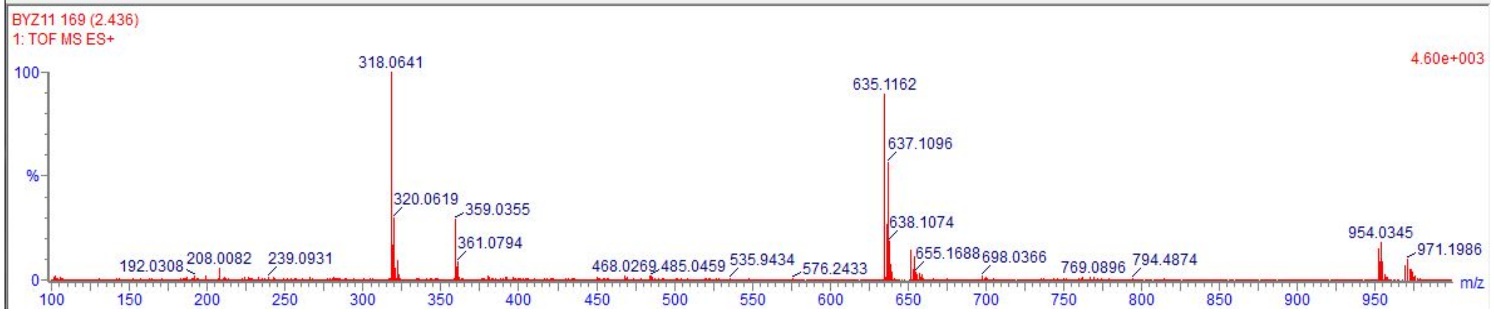

Figure S40. ^{1}H-NMR Spectra of 37

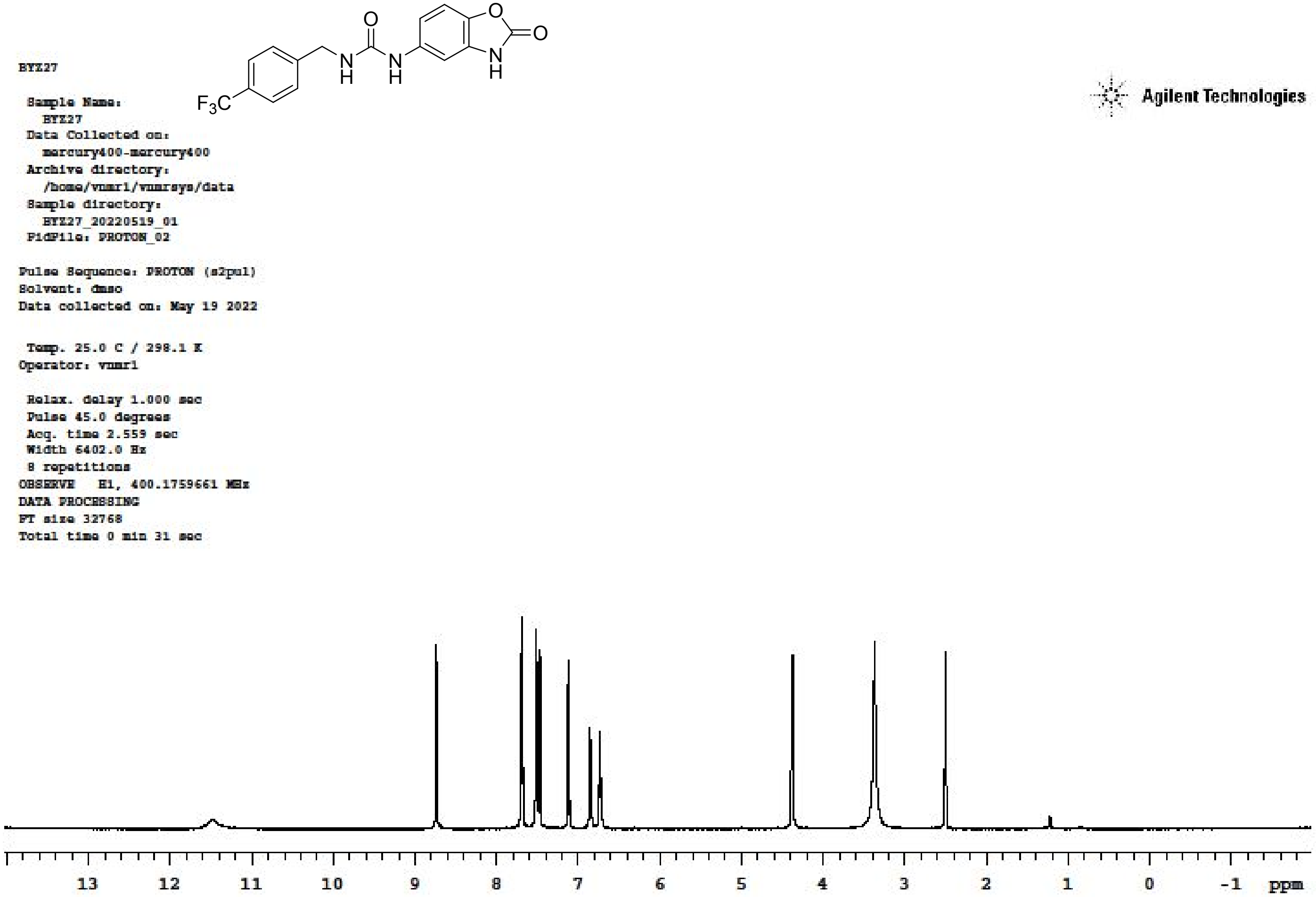

Figure S41. ^{13}C-NMR Spectra of 37

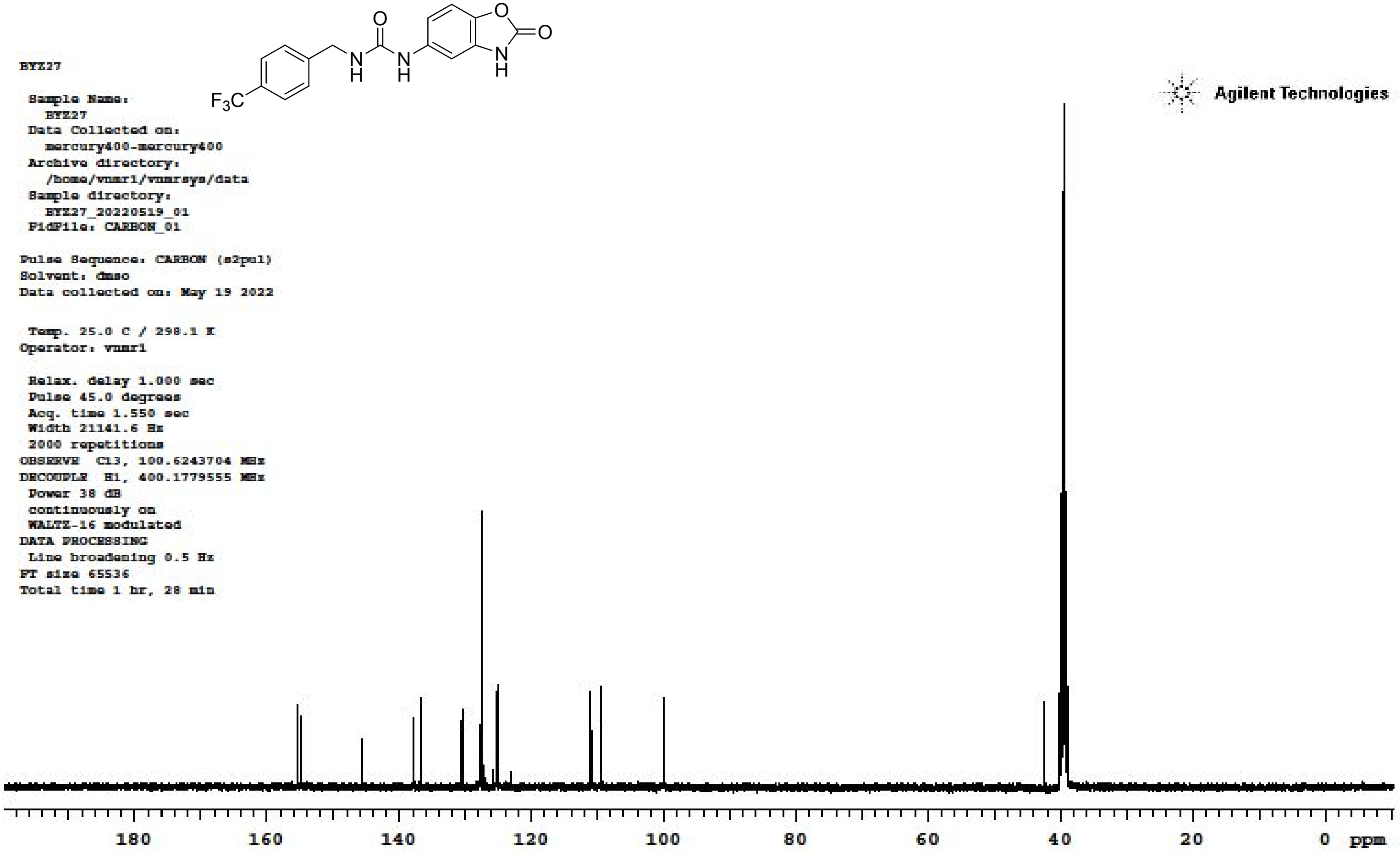

Figure S42. HRMS Spectra of 37

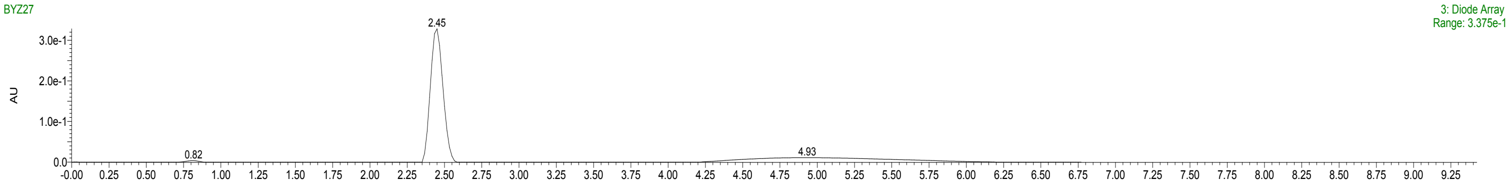

Single Mass Analysis
Tolerance = 5.0 PPM / DBE: min = -1.5, max = 50.0
Element prediction: Off
Number of isotope peaks used for i-FIT = 3
Monoisotopic Mass, Even Electron Ions
47 formula(e) evaluated with 1 results within limits (up to 50 closest results for each mass)
Elements Used:

Mass	Calc. Mass	mDa	PPM	DBE	Formula	i-FIT	i-FIT (Norm)	C	H	N	O	F
352.0917	352.0909	0.8	2.3	10.5	C16 H13 N3 O3 F3	337.7	0.0	16	13	3	3	3

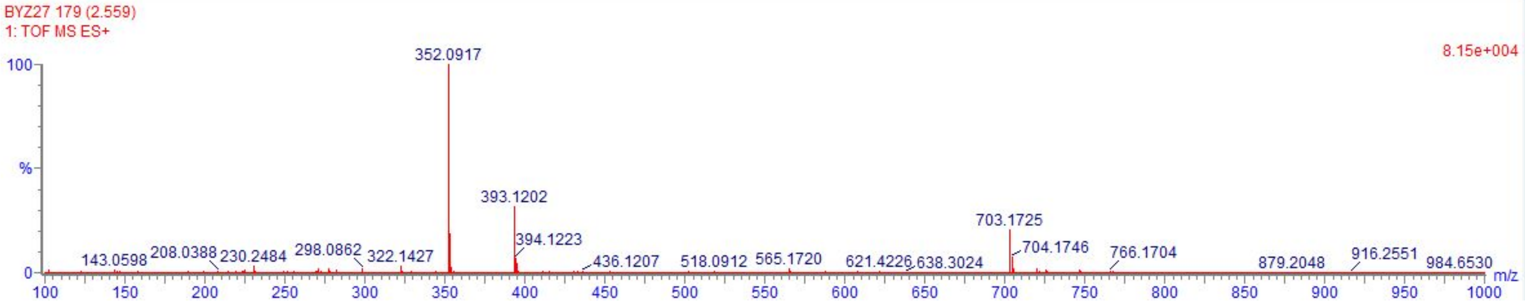

Figure S43. ^{1}H-NMR Spectra of 38

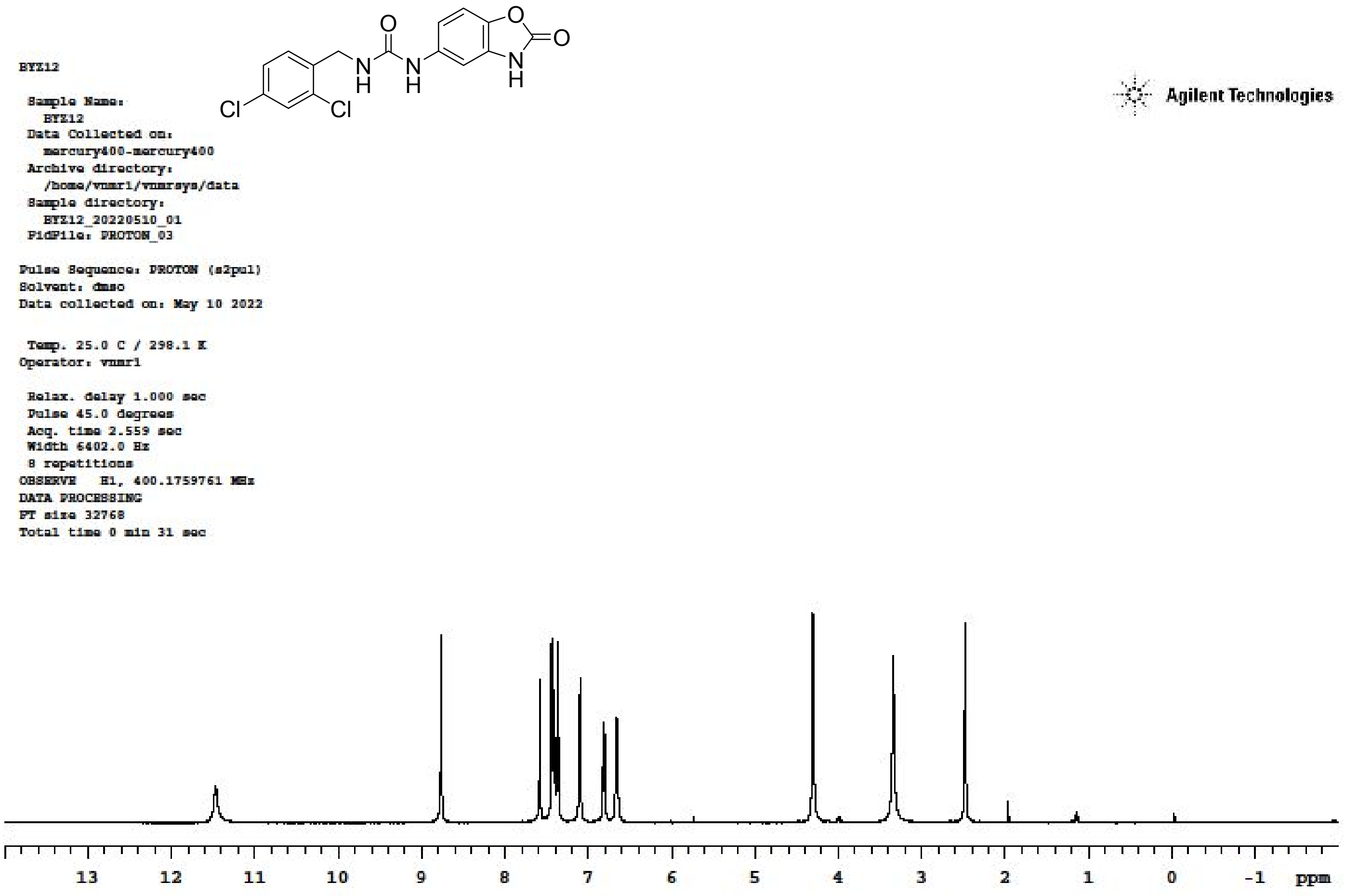

Figure S44. ^{13}C-NMR Spectra of 38

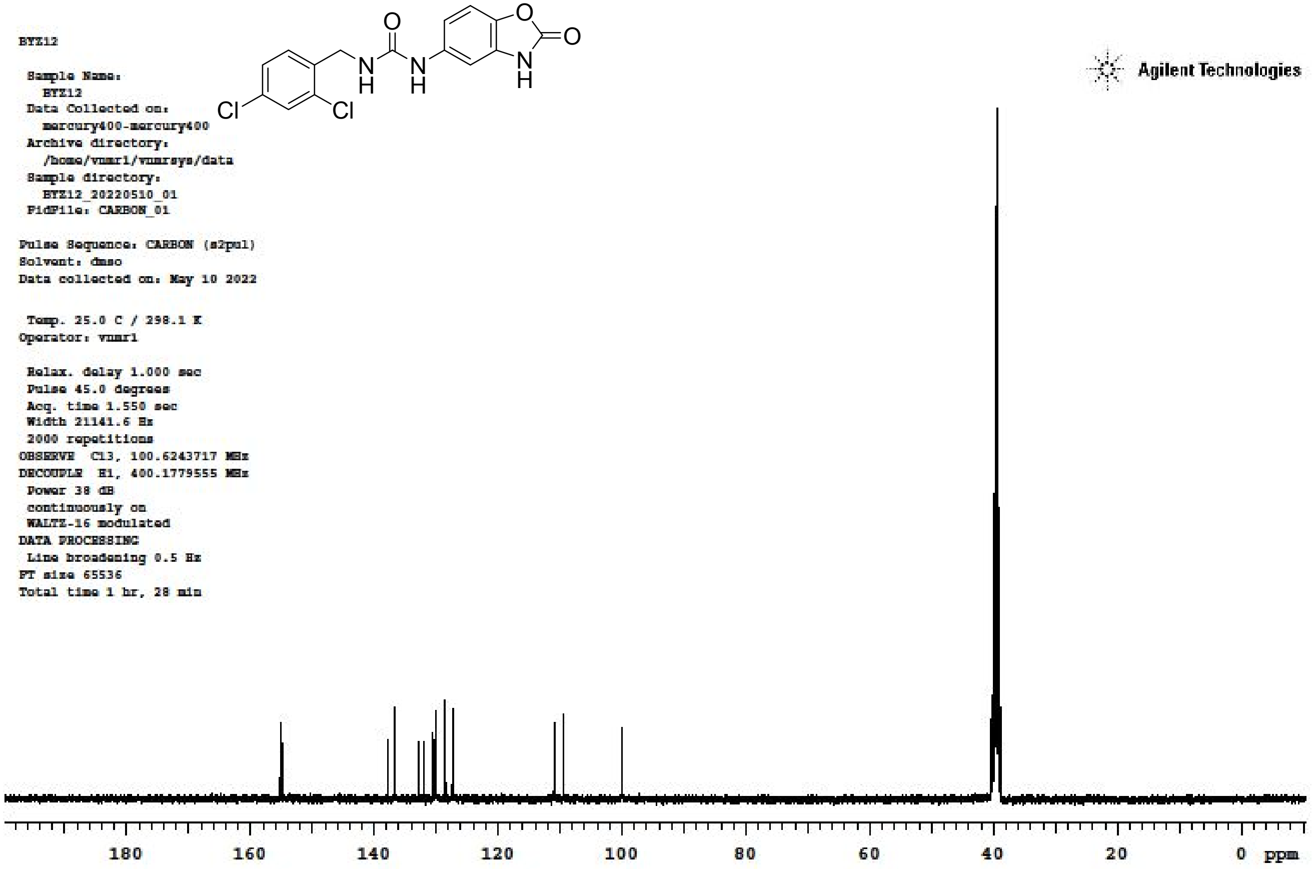

Figure S45. HRMS Spectra of 38

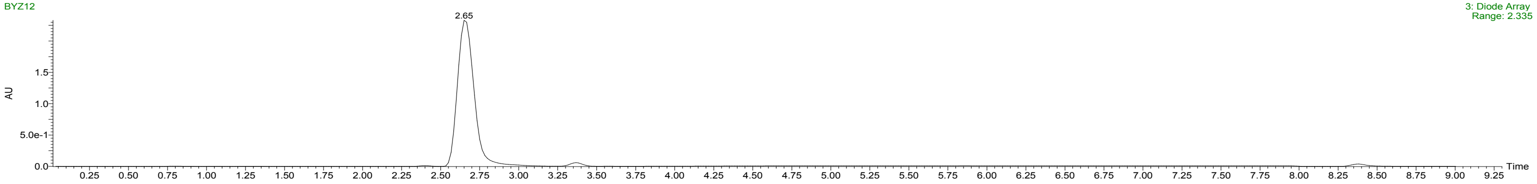

Single Mass Analysis

Tolerance = 5.0 PPM / DBE: min = -1.5, max = 50.0

Element prediction: Off

Number of isotope peaks used for i-FIT = 3

Monoisotopic Mass, Even Electron Ions

16 formula(e) evaluated with 1 results within limits (up to 50 closest results for each mass)

Elements Used:

Mass	Calc. Mass	mDa	PPM	DBE	Formula	i-FIT	i-FIT (Norm)	C	H	N	O	Cl
352.0259	352.0256	0.3	0.9	10.5	C15 H12 N3 O3 Cl2	67.1	0.0	15	12	3	3	2

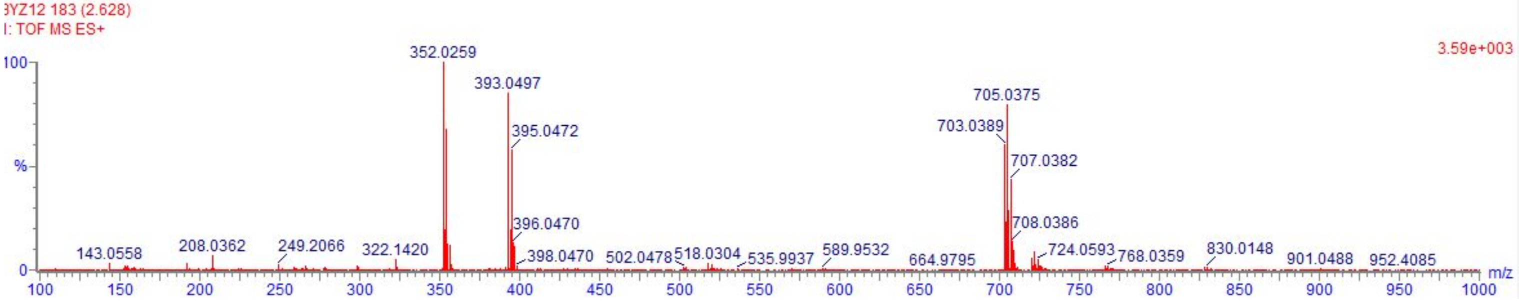

Figure S46. ^{1}H-NMR Spectra of 39

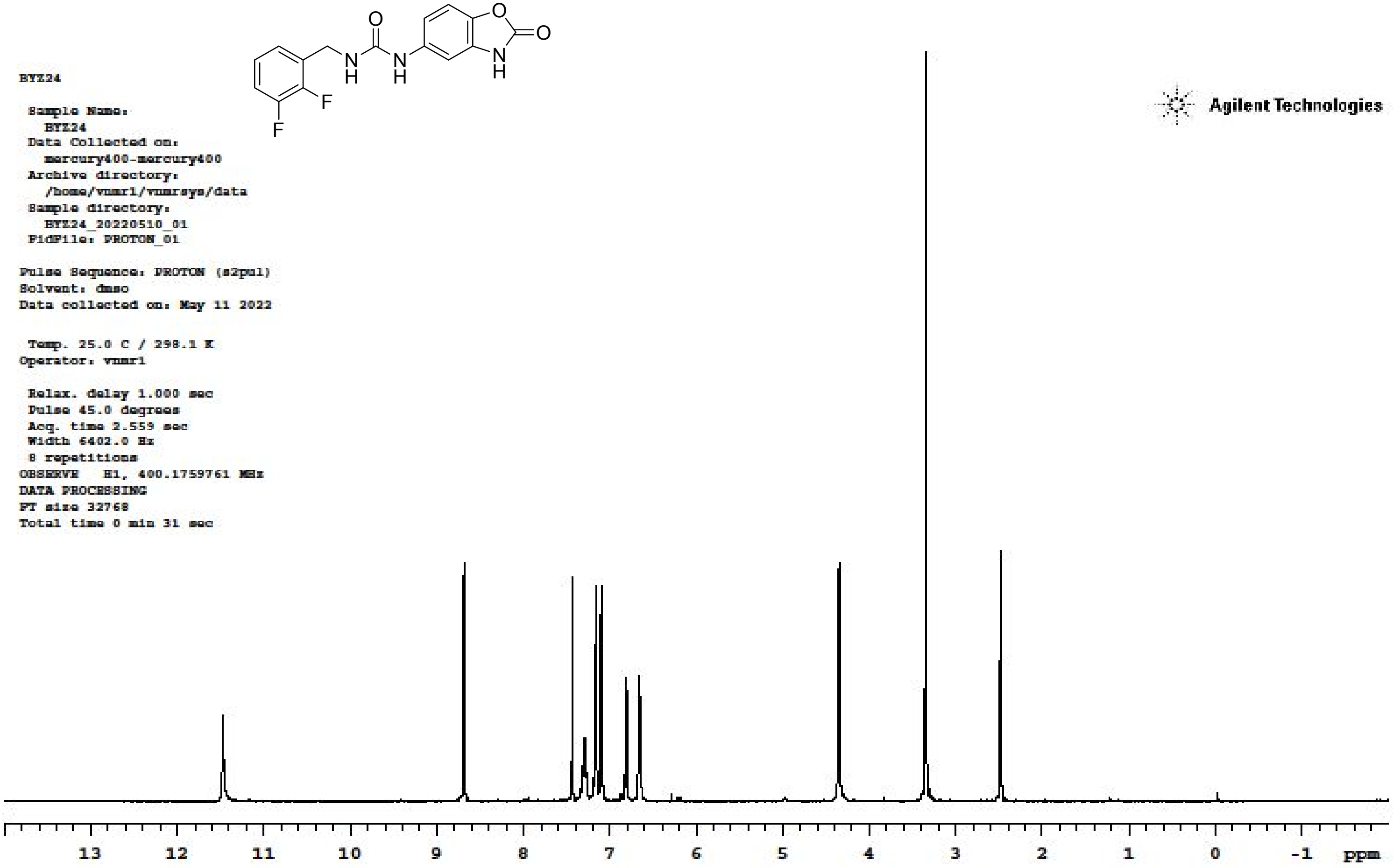

Figure S47. ^{13}C-NMR Spectra of 39

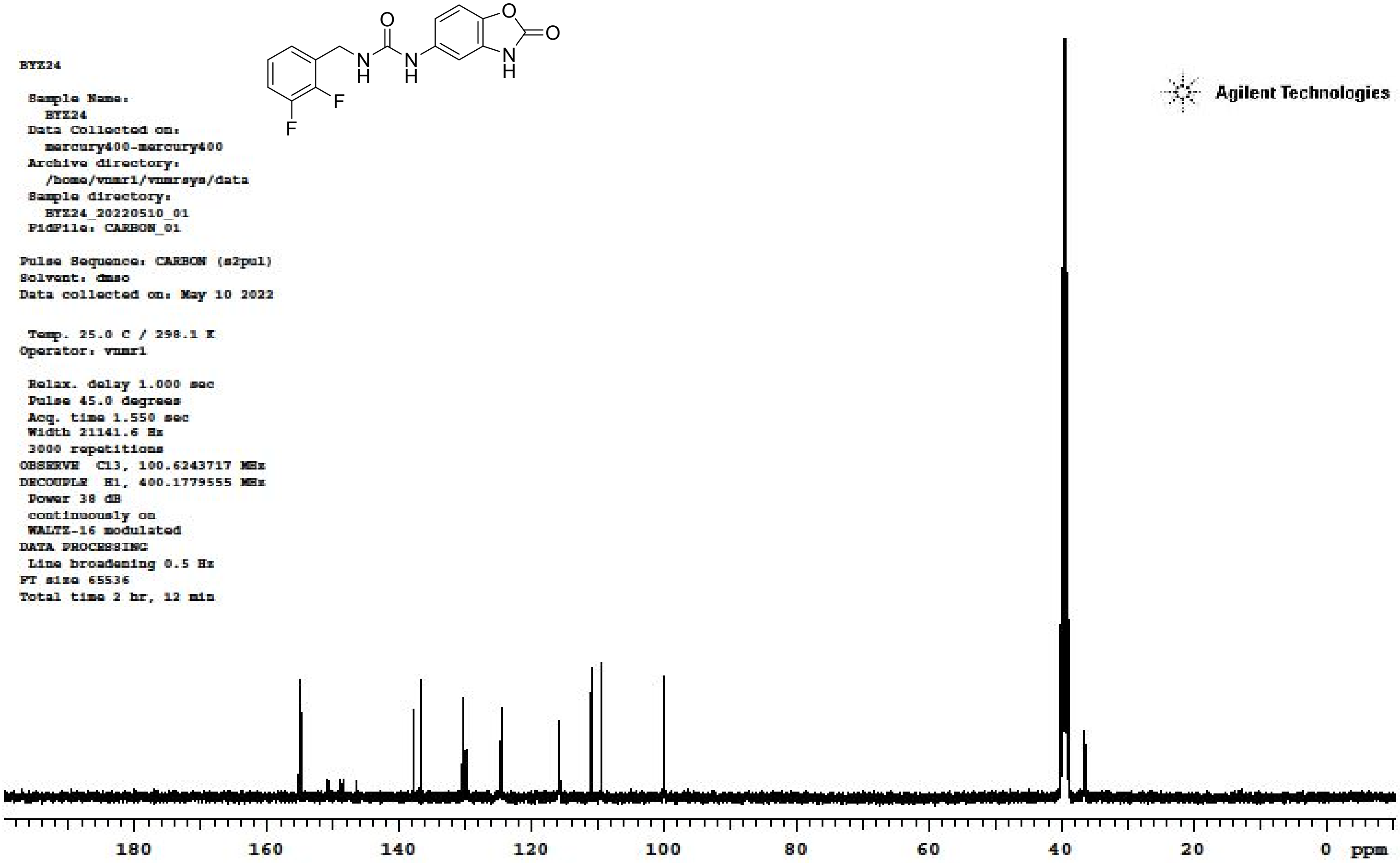

Figure S48. HRMS Spectra of 39

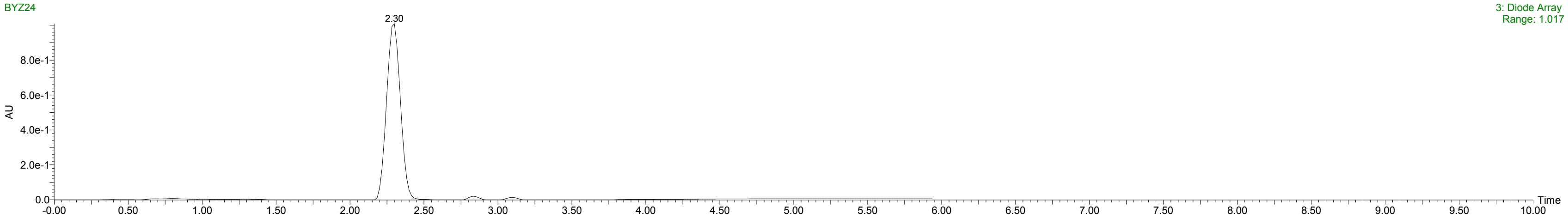

Single Mass Analysis

Tolerance = 5.0 PPM / DBE: min = -1.5, max = 50.0
Element prediction: Off
Number of isotope peaks used for i-FIT = 3
Monoisotopic Mass, Even Electron Ions
27 formula(e) evaluated with 1 results within limits (up to 50 closest results for each mass)
Elements Used:

Mass	Calc. Mass	mDa	PPM	DBE	Formula	i-FIT	i-FIT (Norm)	C	H	N	O	F
320.0847	320.0847	0.0	0.0	10.5	C15 H12 N3 O3 F2	196.0	0.0	15	12	3	3	2

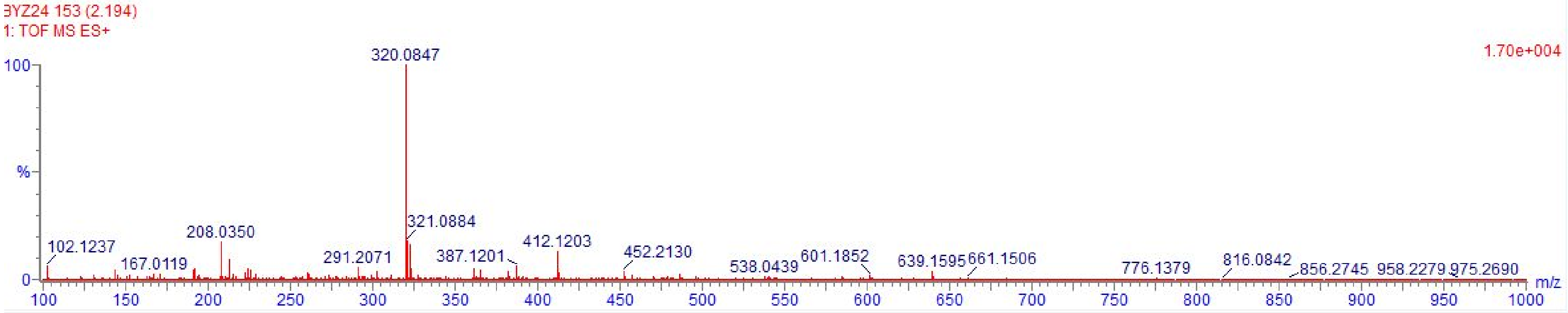

Figure S49: The interactions observed between compound **33** and protein throughout the simulation are summarized below, categorized by type. Over the course of the trajectory, the stacked bar charts are normalized; for instance, a value of 0.7 indicates that a particular contact is maintained 70% of the time during simulation. As some protein residues may make several interactions of the same subtype with the ligand, values higher than 1.0 are feasible.

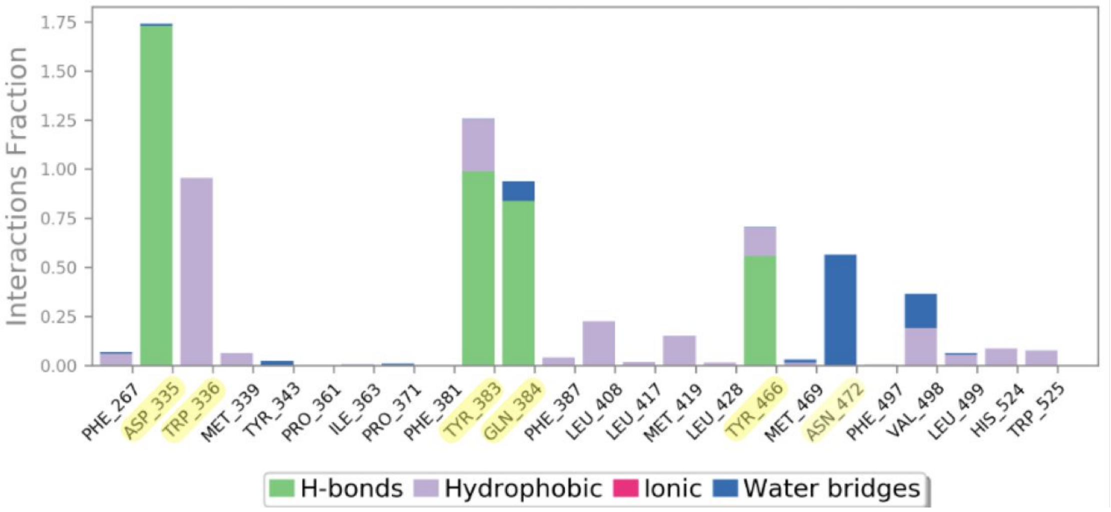

Figure S50: The 2D schematic representation of compound **33** with color-coded rotatable bonds is displayed below. A dial plot and bar plot of the same color accompany each rotatable bond torsion. The conformational evolution of each rotatable bond in the ligand during the simulation trajectory is summarized by the ligand torsions figure (0-200 ns). Dial (or radial) charts show how the torsion has changed over the course of the simulation. The time evolution is represented radially outwards from the simulation's starting point in the center of the radial map. By displaying the probability density of the torsion, the bar plots provide a summary of the data on the dial plots.

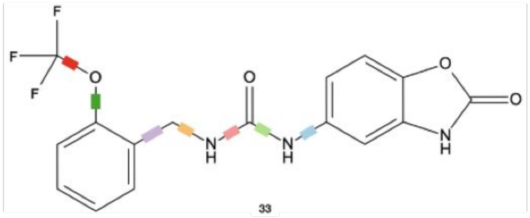

33

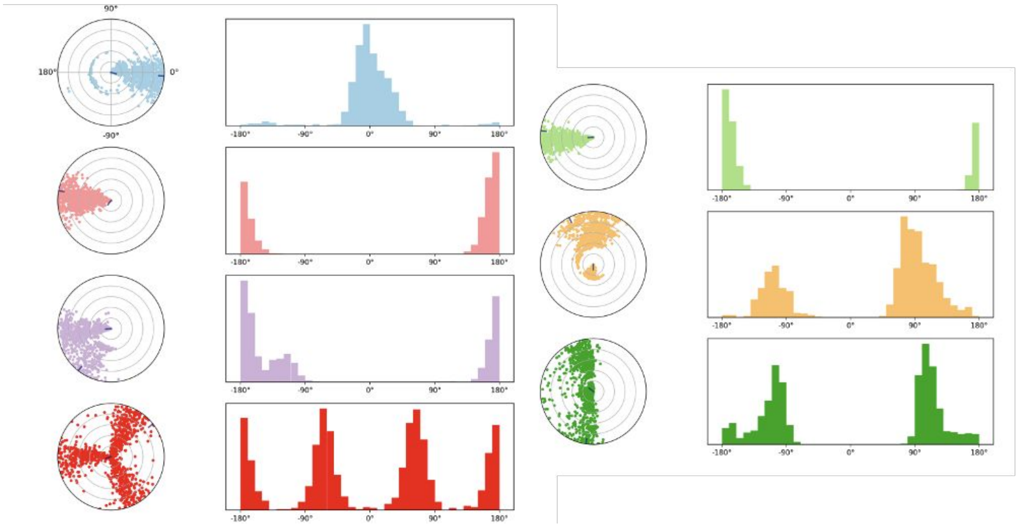

Figure S51: RMSD plots of compound 33 simulated with sEH enzyme (PDB ID:4OCZ) for four MD simulations. The simulation time of 200 ns showing the formation of stable complex without any significant conformational changes in protein structure.

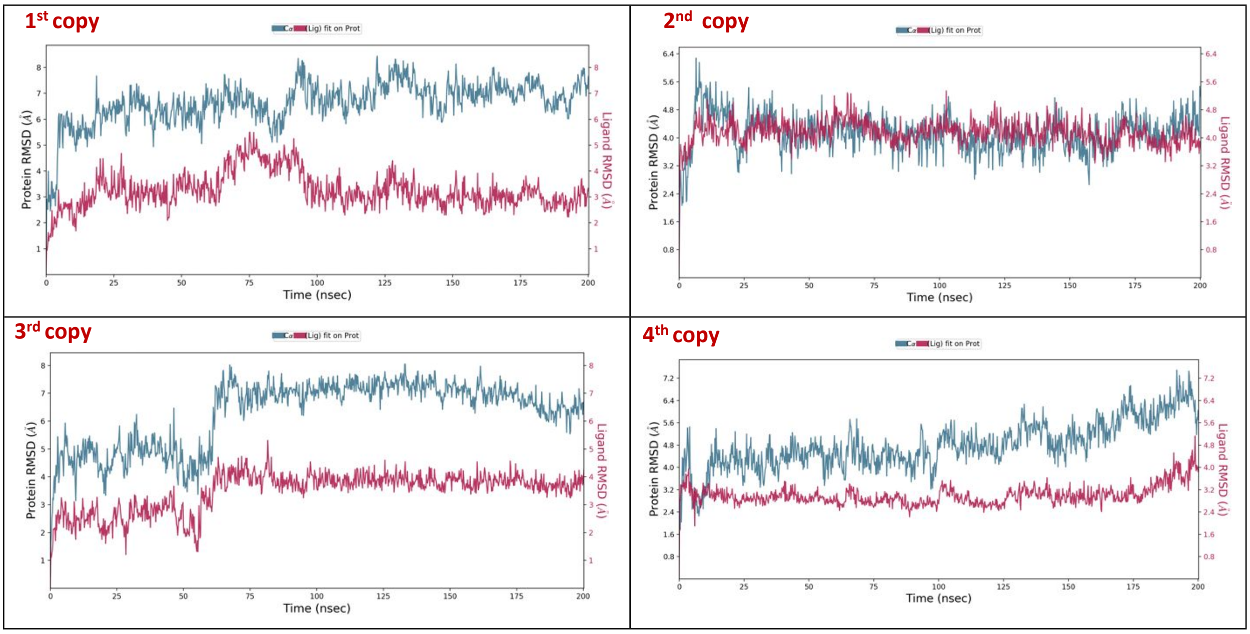

Figure S52: Effects of compound 33 and 38 on cell viability of human monocytes. 2 x 10^5 monocytes were incubated with different concentrations of compound **33** and **38**, triton (0.1%) as positive control or vehicle (0.1% DMSO) for 24 h at 37°C (5% CO_2). After addition of MTT solution, cells were incubated for 2 h at 37°C and lysed in an SDS-containing buffer (10% w/v). Cell viability is represented by reduction of MTT and shown as percentage of vehicle control, n = 3 separate donors.

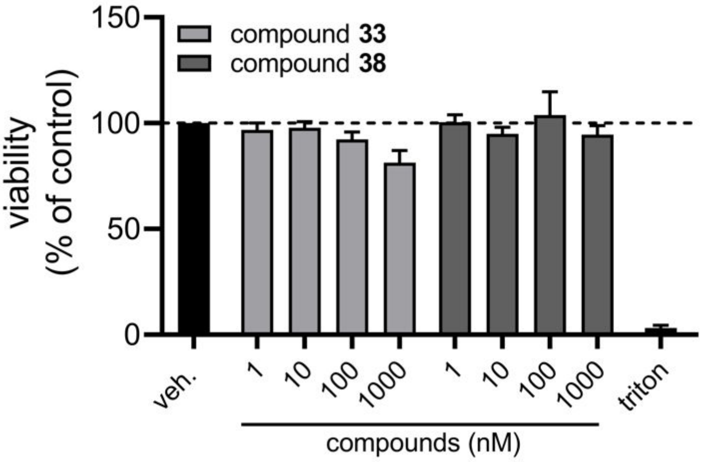

REFERENCES

(1) Varun, B. V.; Gadde, K.; Prabhu, K. R. Sulfenylation of β-Diketones Using C–H Functionalization Strategy. *Organic Letters* **2015**, *17* (12), 2944-2947.

(2) Abdelaal, S. M.; Kong, S.-B.; Bauer, L. Synthesis of 1-[3-methyl-2(3H)-benzazolon-5- or 6-yl]-4-{4-[cis-2-(2,4-dichlorophenyl)-2-(1H-imidazol-1-yl-methyl)-1,3-dioxolan-4-yl]methyleneoxyphenyl}piperazines. *Journal of Heterocyclic Chemistry* **1992**, *29* (5), 1069-1076.

(3) Thomas, R., C.; Poel, T.-J.; Barbachyn, M., R.; Gordeev, M., F.; Luehr, G., W.; Renslo, A.; Singh, U. N-Aryl-2-oxazolidinone-5-carboxamides and their derivatives and their use as antibacterials. WO2003072553A1, 2003.

(4) Venter, J.; Perez, C.; van Otterlo, W. A. L.; Martínez, A.; Blackie, M. A. L. 1-Aryl-3-(4-methoxybenzyl)ureas as potentially irreversible glycogen synthase kinase 3 inhibitors: Synthesis and biological evaluation. *Bioorganic & Medicinal Chemistry Letters* **2019**, *29* (13), 1597-1600.
